# Improving CAR T cell therapy against malignancies through gene knock-down/out strategies: a systematic review

**DOI:** 10.1186/s12935-025-04090-5

**Published:** 2025-12-07

**Authors:** Amirali Karimi, Sayedeh-Zahra Kazemi-Harikandei, Sanam Alilou, Dorsa Salabat, Seyed Morteza Pourfaraji, Fatemeh Ojaghi Shirmard, Niloofar Seighali, Saba Maleki, Behnia Akbari, Farshid Noorbakhsh, Jamshid Hadjati, Hamid Reza Mirzaei

**Affiliations:** 1 School of Medicine, Tehran University of Medical Sciences, Tehran, Iran; 2 Non-Communicable Disease Research Center, Alborz University of Medical Sciences, Karaj, Iran; 3 Tehran Heart Center, Cardiovascular Disease Research Institute, Tehran University of Medical Sciences, Tehran, Iran; 4 School of Medicine, Guilan University of Medical Sciences, Rasht, Iran; 5 Cardiac Primary Prevention Research Center, Cardiovascular Diseases Research Institute, Tehran University of Medical Sciences, Tehran, Iran; 6 Vilcek Institute of Graduate Biomedical Sciences, New York University Grossman School of Medicine, New York 10016 New York, USA; 7 Department of Medical Immunology, School of Medicine, Tehran University of Medical Sciences, Tehran, Iran; 8 Department of Nuclear Medicine, The University of Texas MD Anderson Cancer Center, Texas 77054 Houston, USA

**Keywords:** CAR T cell, Cancer immunology, Gene editing, Knock-out, Knock-down

## Abstract

**Background:**

CAR T cells still face numerous obstacles in treating hematologic and solid malignancies. Although gene editing technologies have improved CAR T cell therapy, there are currently no systematic reviews to broadly address preclinical and clinical outcomes of gene-edited CAR T cells. Therefore, we aimed to systematically review the preclinical and clinical studies that evaluate the outcomes of knocked-out/knocked-down (KO/KD) CAR T cells.

**Methods:**

This study was submitted to international Prospective Register of Systematic Reviews (PROSPERO) with the ID CRD42022320541 and follows the Preferred Reporting Items for Systematic Reviews and Meta-Analyses (PRISMA) 2020 guidelines. We searched Five databases (PubMed, EMBASE, Cochrane Library, Web of Science, and Clinicaltrials.gov) up to March 19th, 2022 for the keywords of “CAR T cell” and “knock-out/knock-down”. The retrieved records then underwent a two-step screening process based on the inclusion criteria, first title/abstract and then full-text screenings, and their data were used for qualitative synthesis.

**Results:**

Our search results yielded 3780 records. Finally, a total of 241 records, including 193 animal and 52 human studies (four concurrent in both groups) that reported KO/KD genes for 105 proteins were included. The positive effects of these 105 KO/KD were categorized into five groups: (1) enabling allogeneic CAR production while limiting GVHD, (2) increasing the efficacy of CAR T cells, (3) Decreasing their side effects, (4) limiting CAR T cell fratricide, and (5) enabling the use of concurrent therapies. In the human section, solid tumors had fewer studies with less favorable outcomes compared to hematologic malignancies.

**Conclusions:**

This systematic review emphasized the various mechanisms by which CAR T cell effects could be boosted. Future researchers can choose their desired genes out of the 105 mentioned candidates. We also encourage the researchers to increase their efforts on solid tumors to compensate for the lack of increased efficacy in this group.

**Supplementary Information:**

The online version contains supplementary material available at 10.1186/s12935-025-04090-5.

## Introduction

Chimeric antigen receptor (CAR) T cell therapy produced unprecedented outcomes in patients with hematologic malignancies. Kymriah (tisagenlecleucel) became the first approved CAR T cell in August 2017 for treatment of B-cell precursor acute lymphoblastic leukemia (ALL) in patients below 25 years [1]. These successes continued by approval of seven CAR T cell therapies for malignancies as of 2025, all of which were directed against CD19 or B-cell maturation antigen (BCMA) [2, 3]. The list includes five anti-CD19 CAR T cells Tisagenlecleucel, Axicabtagene ciloleucel, Brexucabtagene autoleucel, Lisocabtagene maraleucel, and Obecabtagene autoleucel, and two anti-BCMA CAR T cells Idecabtagene vicleucel and Ciltacabtagene autoleucel [4].

A recent retrospective cohort on the efficacy of axicabtagene ciloleucel, tisagenlecleucel, and lisocabtagene maraleucel for relapsed/refractory large B cell lymphoma (LBCL) reported a range of 63–93% and 50–72% for the objective response rates (ORR) and complete response rates (CRR), respectively. Estimated 2-year progression-free survival (PFS) and overall survival (OS) ranged from 30–46 and 45–63% [5]. For anti-BCMA, phase II KarMMa‑1 on idecabtagene vicleucel and phase Ib/II CARTITUDE‑1 trial on ciltacabtagene autoleucel reported ORR of 73% and 98%, respectively [6]. Nevertheless, disease progression/recurrence is the usual eventual outcome in the majority of patients, reflected in the aforementioned PFS results of anti-CD19 CAR T cells, the median PFS of 8.8 months for idecabtagene vicleucel [7], and the 5-year PFS of 33% in the CARTITUDE‑1 trial [8].

Despite these advances, CAR T cell have yet to provide groundbreaking outcomes in solid tumors [9]. Several trials in solid tumors targeting different antigens, including Claudin 18.2, Mesothelin, PSMA, EGFR/HER2, and CEA, usually reported ORR of around 30 to 60% and median PFS of around 6 to 12 months, depending on the dosage, tumor type, and target [10–13]. Challenges to name include poor penetration and persistence, weak proliferation, immunosuppressive tumor microenvironment hindering CAR T cell’s effectiveness, adverse outcomes related to treatment [14, 15]. Therefore, researchers have resorted to various solutions to increase the safety and efficacy of CAR T cells. Knocking-down/out (KO/KD) genes are among the approaches to achieve this goal [14, 16].

CAR T cells can trigger a broad range of adverse events in various organs related to immune-related and off-target effects [17]. Two of the most important CAR T cell-related toxicities are cytokine release syndrome (CRS) and immune-effector cell–associated neurotoxicity syndrome (ICANS). In hematologic and solid malignancies, most patients usually developed CRS of any grade, while these toxicities were usually manageable and fewer patients experienced (G3) or higher CRS and ICANS [6, 10–13]. A recent meta-analysis on non-Hodgkin lymphoma estimated the pooled incidence for CRS and ICANS of any grade at 90% and 51%, with G3 or above CRS and ICANS being 7% and 19%, respectively [18].

Beyond oncology, CAR T cell therapies are gaining ground in treating other conditions, such as autoimmune diseases and HIV [19–21]. One example is CD19-directed CAR T cell therapy used for the treatment of 15 patients with systemic lupus erythematosus, idiopathic inflammatory myositis, or systemic sclerosis that produced considerable response in all patients [22]. Understanding CAR T cell therapy challenges and solutions in malignancies can also guide the current and future efforts in treating these diseases.

Gene therapy, including KO/KD strategies, can provide several advantages to overcome the shortcomings associated with CAR T cell therapy, including adding the possibility to produce off-the-shelf allogeneic CAR T cells, increasing CAR T cell efficacy through enhancing its expansion, tissue penetration, persistence, or tumor cell lysis, decreasing the related adverse events, such as CRS and ICANS, and providing the opportunity to target new antigens that are also expressed on T cells without inducing CAR T cell fratricide [14, 23]. Despite the potential benefits of this approach, no comprehensive systematic review exists on this matter to address the advantages of gene KO/KD in CAR T cell therapy in malignancies and show that silencing which targets can achieve these goals. Therefore, we aimed to systematically review the animal or human studies that utilized gene KO/KD in CAR T cells and discover the impacts of these gene-editing-based strategies on the therapeutic efficacy of CAR T cells in hematologic and solid tumors.

## Methods

We registered this study in International prospective register of systematic reviews (PROSPERO) with the ID of CRD42022320541. This systematic review adhered to the Preferred Reporting Items for Systematic Reviews (PRISMA) 2020 guidelines (PRISMA 2020 checklist available in the supplementary material).

### PICO

P (Population/participants): All the patients with malignancies of any kind.

I (intervention/exposure): Receiving gene KO/KD CAR T cells.

C (Comparator/control): Receiving CAR T cells without gene KO/KD (not necessary).

Outcomes: Any outcomes studied by the articles, including safety measures, efficacy endpoints, etc.

### Search strategy

Keywords were chosen using the previous published studies, as well as Medical Subject Headings (MeSH) and Embase’s Emtree. We searched five databases of PubMed, Embase, Cochrane, Web of Science, and Clinicaltrials.gov on July 20th, 2025, for the keywords related to “Chimeric antigen receptor T cells” and “gene knock-out/knock-down”. Search strategies for all databases are presented in the supplementary material.

### Inclusion/exclusion criteria

We included the original studies on humans or animals that investigated the effects CAR T cells bearing gene KO/KD in any type of malignancies. We also included abstracts/conference abstracts that presented a novel original data related to the aim of this study, as the field expands rapidly and many novel studies are first presented in the conferences. Therefore, the exclusion criteria were the following:

1. Non-original studies, including reviews, meta-analyses, and opinions
2. Studies not on humans or animals, including laboratory in vitro studies
3. Studies not on CAR T cells, e.g. those on non-CAR T cells, those on other CAR cells such as CAR NK cells, etc.
4. Studies on diseases other than malignancies, e.g. autoimmune diseases
5. Studies not utilizing KO/KD techniques, including classic CAR T cells without gene editing or those using gene overexpression techniques
6. Duplicate studies, meaning that the same data were reported in more than one study; in case the duplication occurred in an abstract and the corresponding full-text original study, the abstract was excluded. If two original studies utilized the same data and settings, the older was excluded.

### Study selection

The records identified from database searching were downloaded into an EndNote 20 library. The duplicate records were first removed using the EndNote application and then manually. Then, two researchers began the screening process. The screening process consisted of two steps; first, the title and abstracts of the articles were examined if they adhered to the inclusion criteria. The suitable articles entered the second part of the process; their full texts were inspected and the eligible studies were included in this systematic review.

During the process, the two researchers mentioned their reasons on why each record was excluded. In case of discrepancy, the other researcher would read the former researcher’s comment and they would reconsider the opinions to solve the conflict. If the issue remained unresolved, they contacted each other and tried to resolve the conflict. If the disagreement still remained, they would contact another independent researcher for the final decision.

### Data extraction

Two different word tables, one for animal and one for human studies, were designed before the data extraction process. The summary of the variables we sought data for are presented in Supplementary Table 1 (for animal studies) and Table 1 (for human studies).

**Table 1:** Summary of the findings in clinical studies

Study	Year of publication and country	Target cancer	Target antigen	Disrupted gene(s)	Population and design	Main findings
NCT03545815 [53]	2021, China	Mesothelin-positive solid tumors	Mesothelin	PD-1, TRAC	Phase I: 15 patients previously failed ≥ 1 chemotherapy regimen and 25 total doses of MPTK-CAR-T cells: Median age: 59 6 pancreatic cancer, 3 biliary tract cancer, 6 others with gastric, cervical, tubal, ovarian, esophageal, and triple-negative breast cancers No prior lymphodepletion	1. Median OS: 3 months RECIST 1.1: Stable disease in 7 in 3–4 weeks (median PFS: 7.1 weeks, median OS: 4.9 months), only in 2 in 8–12 weeks (best objective response); 9 progressive disease 2. No dose-limiting toxicity, grade 3 or higher AEs, autoimmunity, CRS, or neurotoxicity 3. TCR- cells were the minority in patients’ blood (TCR knockdown caused disadvantages in engraftment and/or proliferation)
NCT03747965 [254] Abstract	2020, China	Mesothelin-positive Solid Tumors	Mesothelin	PD-1	Phase I, 9 patients (6 pancreatic cancers, 2 ovarian cancers, 1 colorectal cancer) received three different cohorts (cells/kg): 1) 3 patients (1–2 × 10^6^), 2) 4 (0.5–1 × 10^7^), 3) 2 (2.5–5 × 10^7^) 8 had prior lymphodepletion and 4 received repeated infusion	1. Best response (evaluated in 7): Stable disease in 4 patients (median PFS: 80 days), partial response in 2 (median PFS: 160 days, dosed ≥ 1 × 10^7^) 2. Only 2 G1 CRS in cohort 3 3. Peak expansion of CAR T cells in days 7–14, undetectable by 1 month (demonstrating no significant improvement by PD-1 disruption)
NCT04213469 [242]	2023, China	Relapsed/refractory B-cell NHL	CD19	PD1	Phase I clinical trial −3 + 3 dose escalation & expansion- autologous CAR-T cells, LD with fludarabine and cyclophosphamide, followed by 2 × 10^6^/kg, 4 × 10^6^/kg, 6 × 10^6^/kg (14 patients) for dose escalation, and dose expansion cohort receiving optimal dose 2 × 10^6^/kg (7 patients)	1. 17/21 (81%) had CR, 21/21 (100%) had an objective response (ORR = 100%), all patients had response at the 1 month follow-up except one showing response after 2 months. 16 patients remained on CR after 3 months From 17 patients with DLBCL, 13 had CR (76%) Total of 11 patients had relapse, 1 with CD19 + disease Treatment induced CR in 3/4 (75%) patients with PD-L1 positivity, with persistent response 2. Median PFS 19.5 months, the estimated OS not reached (76% OS rate at 12 months). PFS was not associated with prolonged B cell aplasia. No significant differences across DL regarding PFS DLBCL patients had a median PFS of 18.2 months 3. CAR-T cells showed expansion with a peak between days 9 and 28. The persistence of B cell aplasia was 70% and 40% at 6- and 12-month follow-up, respectively. Eight patients had increased B cell counts during follow-up, 4 of which showed relapse The expansion and persistence of CAR-T cells were not associated with DL 4. G1-2 CRS: 14/21 (67%) G1-2 ICANS in 4/21 (19%), all resolved without subsequent neurotoxicity. Other toxicities: > G2 cytopenias which were manageable. Two cases had infections, with one death attributable to B cell aplasia No DLT observed
NCT05741359, NCT04213469 [243] Abstract	2024, China	Relapsed/refractory B-cell NHL	CD19	PD1	NCT04213469: Phase I clinical trial −3 + 3 dose escalation & expansion- autologous CAR-T cells, LD with fludarabine and cyclophosphamide, followed by 2 × 10^6^/kg, 4 × 10^6^/kg, 6 × 10^6^/kg (14 patients) for dose escalation, and dose expansion cohort receiving optimal dose 2 × 10^6^/kg NCT05741359: An ongoing phase I/II study	Reports from the NCT04213469 as reported above. Within the additional follow-up time extending to 39 months, 18/21 (85%) had CR, and 7 maintained CR at the data cutoff In the ongoing clinical trial (NCT05741359), 9 patients were administered BRL-301, with no DLT at 28 days
[217] Abstract	2017, China	Relapsed/refractory B-cell ALL	CD19	IL-6	Total of 13 participants with similar tumor burden, 6 received regular and 7 received shRNA-induced IL-6-knockdown anti-CD19 CAR T cell: 5–10 × 10^6^ CAR-T cells/kg in three consecutive days (10%−30%−60%)	1. All patients achieved complete response 2. Any grade CRS observed in 6/6 & 6/7 patients. However, grade 3 or higher CRS occurred in 5/6 (83.3%) and 3/7 (42.8%) of regular and IL-6-knockdown groups, respectively. Tocilizumab was given to 4/6 patients in regular CAR T group, and 2 required more than one dose No CAR-related deaths 3. Significantly lower serum IL-6 levels in IL-6 knockdown group (p = 0.0217)
NCT04150497 [218] Abstract	2021, USA	Relapsed/refractory CD22 + B-Cell ALL	CD22	TRAC & CD52	Phase I, dose-escalation trial with blast expression of 70% or more 9 patients either received lymphodepletion with fludarabine and cyclophosphamide (FC) or FC + atezolizumab (FCA), and either 1 × 10^6^ or 2.5 × 10^6^ cells/kg CAR T cells	1. No patient had G3 ≤ AEs or infections except a patient with G3 hyperbilirubinemia and febrile neutropenia. No GVHD or ICANS 2. Patients who received FCA and higher dose of CAR T cells had lymphodepletion during 28 days observation period. 2 patients had significant blast reduction. CAR T cell detection was associated with inflammatory cytokine response
NCT04150497 [225] Abstract	2023, multi-nationality	Relapsed/refractory B-cell ALL	CD22	TRAC & CD52	Phase I clinical trial 3 + 3 dose escalation-updated results from second batch Three patients receiving fludarabine, cyclophosphamide and alemtuzumab, followed by 1 × 10^6^ UCART22 cells/kg (FCA-DL2)	1. 2/3 of patients showed response, one with CR lasting 84 days. One patient had morphologic leukemic-free status and MRD negativity until day 40 2. No DLT or ICANS observed. CRS G1-2 occurred in 2/3 (66%) that was either self-limited or resolved by tocilizumab. G5 sepsis in one patient on day 40-attributable to UCART22 & LD therapy UCART22 cell expansion peaked at day 11, and correlated with inflammatory response
NCT04416984 [238] Abstract	2021, USA	relapsed/refractory large B-cell lymphoma	CD19	TRAC & CD52	Phase I/II in patients ≥ 2 prior lines of therapy: Lymphodepletion with FC + ALLO-647 (anti-CD52), and either 40 × 10^6^ (DL1) or 120 × 10^6^ (DL2) ALLO-501A CAR T cells, plus consolidation ALLO-647 + ALLO-501A dose at 28 d in patients with ≥ stable disease 12 evaluable patients (6 in DL1 & 6 in DL2), 9 received consolidation	1. Response rate Overall: ORR = CR = 50% Response rate in consolidation cohort: ORR = CR = 66.7%, with higher CAR T cell expansion in responders Patients continued to have CAR expansion after the consolidation dose 2. No CRS, ICANS, GVHD, dose-limiting toxicity, or G3 ≤ AEs. Most common AE: cytopenia 72%
NCT03939026 [239] Abstract	2021, USA	relapsed/refractory: large B-cell or follicular lymphoma	CD19	TRAC & CD52	Phase I in patients ≥ 2 prior lines of therapy: Similar to the above study, but 3 doses of ALLO-501 (also 360 × 10^6^ cells (DL3)) 46 patients; Single-dose cohort (n = 39) and consolidation cohort (n = 7) 36 patients received no prior autologous CAR therapy (auto CAR T naïve); Follicular (n = 23) and large B-cell lymphoma (n = 13)	1. Response rates among auto CAR T naïve patients: ORR: 75%, CR: 50% Follicular lymphomas: ORR: 82.6%, CR: 52.2% Large B-cell: ORR: 61.5%, CR: 46.2% 4 followed patients after consolidation (all follicular): ORR: 100%, CR: 75% 29/36 followed 6 ≤ m: 27.8% CR in follicular (n = 18) & 36.4% CR in large B-cell (n = 11), longest ongoing CR: 15 + months 2. Only one manageable CRS. No GVHD, dose-limiting toxicity, or G2 ≤ ICANS. G3 ≤ infections in 23.9%. Of 5 patients who died, one seemed ALLO-501-related (Aspergillus pneumonia 263 d post CAR)
NCT03525782 [255] Abstract	2018, China	NSCLC	MUC-1	PD-1	6 patients with stage IIIb to IV NSCLC received 2.5 × 10^6^ cells/kg	1. 2/6 patients had significantly reduced tumor size after 4 weeks. Effects on metastases were limited 2. Serum CYFRA21 decreased after treatment but then increased at 4 weeks 3. No CRS or other AEs in patients, despite increased cytokines in 3/6
[240]	2019, USA	CLL	CD19	TET-2	Case report, a 78-year-old patient with CLL	94% of CAR T cells originated from disrupted TET-2 gene (c.5635C mutation) during lentiviral transduction The patient had no circulating disease or marrow infiltration, with the presence of CAR T cells in the peripheral blood after 4.2 years
[219]	2021, China	CNS B-ALL	CD19	IL-6	Prospective observational study of 12 patients receiving ssCART-19s (IL-6 KO anti-CD19): 3 consecutive 5 × 10^6^ cells/kg CAR injections, then 1 month monitoring for CRS	1. Response rate: 11/12 (91.67%), of which 10 was CR and 1 was PR 2. CRS rate: 100%, but no stage 3 ≤ CRS (3 (25%) stage I, 9 (75%) stage II) 3. Inflammatory cytokines increased 2–3 days after treatment and returned to normal at 10 days 4. Temperature increased in stage II CRS patients and returned to normal after 14 days
NCT02746952 [226]	2022, multi-nationality	Relapsed/refractory B-cell ALL	CD19	TRAC & CD52	Phase I study of 25 patients with lymphodepletion by cyclophosphamide and fludarabine ± alemtuzumab, dose escalations of 6 × 10^6^, 6–8 × 10^7^, 1.8–2.4 × 10^8^ UCART19 cells ± HSCT (9/12 patients with CR or CRi)	1. 12 of 25 patients achieved CR/CRi, 9/12 MRD-neg. Highest percentage of response in DL1 (4/6 (67%) at DL1, 5/12 (42%) at DL2, 3/7 (43%) at DL3). All patients with CR/CRi received alemtuzumab. Median duration of response: 7.4 months 2. Median OS: 13.4 months, median PFS: 2.1 months, at database lock 11 were alive, 6 had ongoing response, none had CD19-neg disease 3. 14 patients had UCART19 expansion, no UCART19 cell expansion in patients not receiving alemtuzumab, persistence beyond 42 days observed in 4 patients (5 by qPCR), no relation between DL and expansion 4. 3 treatment-related deaths (1 multiorgan dysfunction + neutropenic sepsis, 1 pulmonary hemorrhage, 1 sepsis + multiorgan dysfunction) CRS: 20 (80%): 6 (24%) G3-4 CRS 1 G4 neurotoxicity, 6 (24%) G1-2 neurotoxicity, G1 GVHD: 2 (8%). G4 prolonged cytopenia: 4 (16%). Similar safety profiles across the DLs
NCT02746952 NCT02808442 [220]	2020, multi-nationality	Relapsed/refractory B-cell ALL	CD19	TRAC & CD52	Two phase I studies in 21 patients: Lymphodepletion with cyclophosphamide and fludarabine ± alemtuzumab 7 children received 1.1–2.3 × 10^6^ cells/kg UCART19, 14 adults received dose escalations of 6 × 10^6^, 6–8 × 10^7^, 1.8–2.4 × 10^8^ cells/kg	1. 14 (67%) had CR or CR with incomplete hematological recovery at 28 days; Median duration of response: 4.1 months 10/14 underwent allogeneic SCT No antileukemic activity or UCART19 expansion in patients w/o alemtuzumab (n = 4) 6-months: PFS: 27%, OS: 55% 2. Adverse events: 2 treatment-related deaths (1 neutropenic sepsis + CRS, 1 pulmonary hemorrhage + persistent cytopenia) CRS: 19 (91%): 3 (14%) G3-4 CRS, G1-2 neurotoxicity: 8 (38%), G1 acute skin GVHD: 2 (10%), G4 prolonged cytopenia: 6 (32%)
Part of NCT02808442 (above study), included due to detailed patient information [221]	2017, UK	Relapsed/refractory CD19 + B-ALL	CD19	TRAC & CD52	Case report of two infants (11-months-old with high risk t(11;19) B-ALL & 16-months-old with MLL-rearranged B-ALL) who received preconditioning treatment with fludarabine, cyclophosphamide, and alemtuzumab plus TALEN®-mediated anti-CD-19 DKO CARs (UCART19) single-dose (4.6 × 10^6^ & 4.0 × 10^6^ cells/kg, respectively)	1. Both patients had molecular remissions on day 28 Patient 1: negative MRD, still well 18 months after therapy with normal lymphocyte profile and full donor chimerism Patient 2: underwent allo-SCT at 10 weeks and is well at 12 months 2. Patient 1: UCART19 reached 27% in bone marrow of patient 1 by 6 weeks Patient 2: UCART19 in peripheral blood and bone marrow in 2 & 4 weeks, respectively 3. Patient 1: G2 skin GVHD managed with topical and then systemic steroids Patient 2: transient erythematous rash managed with topical corticosteroids, and irritability at 3 weeks with normal CSF tests and imaging
[216]	2022, China	Relapsed/refractory AML	CLL-1	PD-1	Case report of two 28-year-old male patients who failed on multiline salvage therapy, including anti-CD38 CAR T cells, post-transplant failure	1. Both patients demonstrated complete molecular recovery at day 28, with incomplete hematological recovery Both are currently at remission on 8 & 3 months 2. Both had CRS, with grades 1 and 2
[40] Abstract	2020, China	Hematologic malignancies	CD19 or BCMA	TCR & GM-CSF (DKO)	11 patients who received a 2nd generation 41BBζ CAR against CD19 or BCMA with anti-IL6 scFv and IL1RA domains 3 received DKO CARs: 2 with MM & 1 with NHL 8 received no-KO CARs: 5 with ALL, 1 with each of CLL, MM, & DLBCL	1. All patients had complete response except the patient with DLBCL (no response) 2. No patient had neurotoxicity and none required tocilizumab 3. CRS happened in 7 patients: 3 DKO CARs: 2 no CRS, 1 had G2 8 no-KO CARs: 2 no CRS, 1 G1, 2 G2, 3 G3
NCT01082926 [214]	2022, USA	Progressive/recurrent grade III or IV glioblastoma	IL13Rα2	Glucocorticoid receptor	March 2011 to March 2013: Phase I trial of six patients who received intratumoral 10^8^ cells per perfusion of GRm13Z40-2 CARs (days 1, 3, 8, and 10) with glucocorticoid receptor-KO via ZFNs plus aldesleukin (days 2, 3, 4, 5, 8, 9, 10, 11, and 12) and systemic dexamethasone (4–12 mg/day) Patient characteristics: 4 men, all Caucasian, mean (+ SD) age of 56.2 + 10.7 years, mean duration of histological diagnosis: 24 + 27.3 months, KFS: 60–80, with prior temozolomide and radiotherapy	1. MRI or PET: two patients had enhanced tumor necrosis at 4 weeks No objective clinical response or significant survival benefit: median survival after therapy: 2.9 months, longest: 11.9 months (no imaging response in this patient) 2. No dose-limiting or possibly treatment-related > G3 AEs. Only two G1 definitely treatment-related AEs: fever and injection site reaction Other possibly treatment-related AEs: headache (n = 5), fatigue (n = 4), sinus tachycardia (n = 3), and confusion (n = 3), all manageable A patient developed a stroke probably related to dehydration 3. 1/4 patients had Abs against the surface-expressed CAR transgene, suggesting immune rejection of CARs in some patients that may be prevent by higher doses of glucocorticoids
NCT03064269 [222]	2020, China	CD19 + CNS B-ALL	CD19	IL-6	4 patients (two 48-yo females, and two 36 & 65-yo males) conditioned with cyclophosphamide and fludarabine who received 5 × 10^6^ ssCART-19s cells/kg given for 3 consecutive days and monitored for a median follow-up of 2.5 years	1. Dramatic decrease in CD19 expression in all patients. Patients had some improvements in consciousness and CNS impairments (seizure, mass size, brain edema) Patient 1: 3 months to relapse disappeared blasts (relapsed on three months) Patient 2: CR in 12 months Disappeared BCR/ABL fusion gene (relapsed 65 days, again gone after salvage intrathecal 1 × 10^7^ dose) In good health a year after treatment (+ imatinib), died of pulmonary infections duo to leukopenia Patient 3: CR in 10 months Significantly decreased minimal residual disease Died of lung GVHD and infection 10 months after therapy Patient 4: CR un 2. Headaches, generalized fatigue, and fevers within first 10 days of treatment No CRS > G2, no CRES. A G1 ICE & ICANS in all patients No GVHD or severe infections during treatment
NCT04167696 [123] Abstract	2020, USA	Relapsed/refractory AML/MDS	NKG2D	MICA & MICB	6 patients received single-dose infusion of two doses (1 × 10^8^ and 3 × 10^8^ cells) following cyclophosphamide and fludarabine (CYAD-02 had MICA & MICB downregulated by ShRNA compared with CYAD-01)	CYAD-02 was safe and tolerable with no dose-limiting toxicity No data on efficacy presented in the abstract
[241]	2022, China	Relapsed/refractory DLBCL	CD19	β2M & TRAC	Case report of 2 patients (63 & 64-yo males) who received fludarabine and cyclophosphamide conditioning plus total doses of 1.5 × 10^6^/kg and 1.21 × 10^6^/kg cells, respectively	1. P1: low B cell count at ∼7 days post-treatment, patient declined all treatments at day 21 P2: rapid increase in universal CARs after injection, declined at 1 month. Decreased B cell recovered after a month, disease progression by 1 month 2. P1: developed G4 CRS (pulmonary edema & low BP) in the first 21 h not effectively controlled by tocilizumab, etanercept, and methylprednisolone; plus fever, skin damage, swelling and exudate at the site of tumor, which swelling improved with methylprednisolone; plus anemia, thrombocytopenia, and leukocytopenia possibly related to conditioning regimen ameliorated by intensive intervention P2: hyperpyrexia and increased CRP (G1 CRS) improved without intervention
NCT04227015 [223]	2021, China	CD19 + or CD22 + Relapsed/refractory B-ALL	CD19/CD22	TRAC & CD52 Efficacy of editing: 88% & 62%, respectively	6 patients (2 males, 4 females) with median age of 49, median 5 prior lines of therapy, median marrow blast of 52%: preconditioning with fludarabine, cyclophosphamide, and alemtuzumab	1. Day 28: 5/6 CR/CRi (2 CR, 3 CRi) & MRD-, 1/6 NR 5 responders with median 85 [53–202] days follow-up: 4 MRD-, 1 MRD + 2. Peak CAR expansion: day 10–14, lowest peak in the NR patient. Median duration of persistence: 42 days 3. No DLT, ICANS, GVHD, or > G3 AEs All patients had CRS (1 G3, 2 G2, 3 G1), with G3 patient recovered with tocilizumab and glucocorticoid 3 G3 cytopenia, viral reactivation/infections (2 G3 & 1 G2 CMV, 1 G1 adenovirus), 1 G3 bacterial pneumonia, and 1 G3 fungal sepsis
NCT03919240 [227] Abstract	2023, China	Relapsed/refractory B-ALL	CD19	IL-6	Phase I/II clinical trial, LD by fludarabine and cyclophosphamide, followed by 5 × 10^6^/kg IL-6 knocking down CAR19 cells (ssCAR-T-19) or conventional CAR-19 (cCAR-19) T cells (n = 47, 40, respectively)	At baseline, the two group had same percentage of BM blasts (median 4.0) On day 28, 91.5%CR/CRi in ssCAR-19 vs 85% in cCAR-19 group Median PFS 14.17 in the former, and 15.33 months in the latter group (p = 0.339). 3-months PFS significantly longer in ssCAR-19 group (82.3% vs 66.9%, p = 0.045) No significant difference between the two groups regarding peak value, start and duration Higher percentage of high-grade AE in cCAR-19 compared to ssCAR-19: Comparing ssCAR-T and cCAR-T: CRS G3-4: 7/47 (15%) versus 15/40 (37.5%), G1-2: 25/47 (53.2%) versus 19/40 (47.5%) ICANS G1-2: 2/47 (4.26%) versus 4/40 (10%) plus ICANS G3 in 2/40 5%)
Part of NCT03919240 [224]	2020, China	Relapsed/refractory B-ALL	CD19	IL-6	A 29-yo patients with B-ALL extramedullary relapse in skin and testicles who received conditioning with fludarabine and cyclophosphamide plus a total of 5 × 10^6^ cells/kg in three consecutive days	1. Skin nodules disappeared and testicles shirked at 1 week, with skin biopsy showing few lymphocytes with no blasts Patient underwent all-HSCT at day 46, and is in complete remission at 2 years and 1 months 2. Only AEs: G1 CRS (fever), granulocytopenia recovering on day 8
NCT03275493, NCT03919240 [228]	2024, China	Relapsed/refractory B-cell ALL	CD19	IL-6	Post hoc analysis of two phase I clinical trials, LD with fludarabine and cyclophosphamide, followed by 5 × 10^6^/kg IL-6 knocking down CAR19 cells (ssCAR-T-19) or CAR-19 (cCAR-19) T cells (n = 47, 40, respectively) (detailed results mentioned above)	1. 34/47 (72%) from ssCAR-T group and 26/40 (65%) from cCAR-T group became MRD negative. After CAR-T, 20/47 of ssCAR-T and 18/40 of cCAR-T group undergone allo-HSCT therapy 2. 1-year and 2-year survival rates significantly higher in ssCAR vs cCAR group, but not 3-year PFS rates
NCT03275493 (Phase I reported above) [229]	2022, China	Relapsed or refractory B-ALL	CD19	IL-6	A phase II clinical trial in 61 patients with r/r B-ALL. LD by fludarabine and cyclophosphamide, followed by 5 × 10^6^/kg IL-6 knocking down CAR19 cells (ssCAR-T-19)	ssCAR-T-19 induced high CR rate (85.25%) with manageable CRS (81%, including 54% G1-2 and 28% G3-4). More severe CRS associated with higher CAR T expansion. Severe cases resolved by tocilizumab, corticosteroids, ruxolitinib Among patients reaching 36 months, DOR was 56% and OS was rate was 54%
NCT04093596 [236] Abstract	2021, USA	Relapsed/refractory MM	BCMA	TRAC & CD52	Phase I trial of 42 patients with ≥ 3 prior lines of therapy (an immunomodulator, a proteasome inhibitor, and anti-CD38 Ab) with fludarabine and cyclophosphamide lymphodepletion plus 39, 60, or 90 mg TALEN®-mediated DKO anti-BCMA CARs over 3 days	1. 26 patients who received DL3 (320 × 10^6^) or DL4 (480 × 10^6^): ORR: 16 (61.5%), 13/20 (65%), 3/6 (50%) DL4 Very good PR or more: 10 (38.5%, 8 MRD-) Median time to first response: 16 days, median duration of response: 8.3 months 2. 2 deaths (one fungal pneumonia and 1 adenoviral hepatitis) Cytopenia were the most common ≥ G3 AEs 22/42 (52.4%) CRS, no > G3 (and only 1 G3). CRS controlled with tocilizumab (21.3%) and corticosteroids (12.8%) Only 1 G1 neurotoxicity 12.8% ≥ G3 infections
NCT04035434 [249]	2025, multi-nationality	Relapsed/refractory B-cell malignancies	CD19	TRAC & β2M	Phase I/II study of 227 patients in four cohorts (terminated early in phase II, 90 reported) lymphodepletion by cyclophosphamide and fludarabine ± daratumumab, Cohort A-B: 64 NHL patients receiving LD Cohort C: 10 NHL patients receiving LD + daratumumab Cohort D: 16 B-cell ALL patients receiving LD ± daratumumab All followed by CTX110, dose escalated as 3 × 10^7^, 1 × 10^8^, 3–4.5 × 10^8^, 6 × 10^8^ CTX110 cells	1. ORR: 33/56 in cohorts A-B, 5/10 in cohort C, 9/16 in cohort D, Highest duration of response in 1 patient from cohort A at DL2 (9.20 months), Duration of response in cohort C: 2.20 months, Cohort D: 1.56 months 2. Median PFS in cohorts A-B: from 0.92 to 2.83 months (Lowest for DL1, Highest in cohort A at DL4), Median PFS in cohort C: 2.92 months, In cohort D: 1.94 months Median OS in cohorts A-B: ranging from 5.03 to 37.16 (Lowest for DL1). Cohort C: Median OS 7.39 months, Cohort D: 7.62 months 3. Adverse events: almost all patients (except one) experienced AE. Serious AE in 20/64 in cohorts A-B, 5/10 cohorts C, 7/16 cohort D DLT: 1 in cohorts A-B (DL4) CRS: 9/64 in cohorts A-B (Highest in DL4), 2/10 in cohort C at DL4, 1/16 in cohort D (DL3) Neurotoxicity: 6/64 in cohorts A-B (Highest at DL4), 1/10 in cohort C at DL4, 1/16 in cohort D at DL3
NCT04035434 [247] Abstract	2022, multi-nationality	Relapsed/refractory DLBCL	CD19	TRAC & β2M	A phase I clinical trial -interim results, LD by fludarabine and cyclophosphamide, followed by CTX110, dose escalated as DL1: 3 × 10^7^, DL2: 1 × 10^8^, DL3: 3–4.5 × 10^8^ and 6 × 10^8^ CTX110 cells, a second dose in patient who had SD on day 28	1. CR 11/27 (41%) among patients at DL3, ORR 18/27 (66%) 8 patients received a second dose, all showed CAR-T cell expansion, 5 of these patients had deep clinical response 2. No GVHD. CRS G1-2 in 18/32 patients. ICANS in 3 patients, 2 > G2 Overall, 7 patients had serious AE attributable to CAR-T cells, none increased related to second infusion
[230] Abstract	2023, Brazil	Relapsed/refractory B-cell ALL	CD19/CD22	TRAC & CD52	Phase I dose escalation on 6 patients with B-cell ALL, dose escalation of 1 × 10^6^ or 3 × 10^6^ CTA101 cells per kg	1. 5/6 (83.3%) CR/CRi at 28 days 2. 3/5 (60%) of responders were MRD negative at 4.3 months follow-up 3. CRS observed in 6/6 (100%). No neurotoxicity, GVHD, or DLT
ChiCTR1800020306 [250]	2022, China	Relapsed/refractory B-cell malignancies	CD19	PD1 (KD)	Phase II single arm clinical study in patients with relapsed or refractory B-cell malignancies (8 with DLBCL, 4 with B-ALL, 2 with FL, one with CLL and one with HGBL). Conditioning regimen included fludarabine and cyclophosphamide, followed by 1–2 × 10^6^cells/kg PD1-KD anti-CD19 CAR-T cells	1. 6/16 (37.5%) achieved CR, 3/4 (75%) of patients with B-ALL had CR, 2/8 (25%) of cases with DLBCL had CR, 1/2 (50%) of FL cases had CR. 3/6 (50%) of responders did not relapse during the follow-up (2 patients with DLBCL, one with FL), the 3 B-ALL responders relapsed 2. No CAR-T-related deaths, CRS in 11 patients (68%). G1 CRS: 7/16 (43.7%), G2 CRS: 4/16 (25%)
NCT03229876 [71] (also preclinical)	2024, China	Relapsed/refractory B-cell malignancies	CD19	UCAR-T: β2M & TRAC n-UCAR-T: TRAC & HLA-A & HLA-B	Phase I clinical trial in patients with relapsed or refractory B-cell malignancies (6 patients with B-ALL, 3 patients with DLBCL), lymphodepletion with fludarabine plus cyclophosphamide ± etoposide, total body irradiation, 1–6 × 10^6^cells/kg n/-UCART cells in two doses (3 patients with B-ALL received nUCAR-T cells, the others UCAR-T cells)	1. 0/6 responders in patients receiving UCAR-T cells. No expansion and antitumor response in the patients after UCAR-T cells Three patients receiving n-UCAR-T cells showed MRD negativity within 14 days (3/3 (100%) CRi), higher expansion compared to patients receiving UCAR-T 2. Patient 7 with B-ALL resistant after multiple lines, showed CRi after n-UCAR-T cell therapy. She achieved peak CAR-T cells at 9th day, but relapsed with CD19 positive disease after 56 days. She responded once again after second batch with LD followed by n-UCAR-T cells, showing minimal toxicity, and finally received allo-HSCT 3. AE: 2/3 of patients G2 CRS responsive to IL-6 receptor blockade. No GVHD/neurotoxicity
NCT03706326 [256] Abstract	2023, China	Advanced esophageal cancer (stage IIIb/IV)	MUC1	PD1	A phase I clinical trial in 9 patients with advanced esophageal cancer, dose escalation of 5 × 10^7^, 5 × 10^9^ CAR-T cells (4 patients single dose, 5 multiple doses)	1. 6/9 (66%) of patients had stable disease and 3/9 (33%) had progressive disease, all had symptom improvement after CAR-T cell infusion, patients receiving multiple cycles had more stable disease, two of the patients receiving multiple doses had OS of 24 months 2. No G3-5 AE was seen. Most common AE were fever (8/9), chills (5/9) and skin rash (4/9)
ISRCTN15323014 [233]	2023, UK	Relapsed childhood T-cell ALL	CD7	TRBC1 & TRBC2 & CD7 & CD52	Phase I clinical trial in children with refractory or relapsed CD7 + T-ALL, ahead of allo-HSCT, lymphodepletion with fludarabine and cyclophosphamide and alemtuzumab, followed by 0.2 × 10^6^ to 2.0 × 10^6^/kg BE-CAR7 cells. Allo-HSCT in patients with molecular remission after 28 days -after second conditioning chemotherapy	1. Patient 1: a 13-year-old girl, previously treated with multiple lines including chemo-radiation and allo-HSCT and relapsed with CD7 + T-ALL prior to trial. The patient received 0.7 × 10^6^/kg BE-CAR7 after LD, with no immediate infusion-related AE. The patient developed early G2 CRS & G1 neurotoxicity. No GVHD observed. G3-4 cytopenia from day 0 onward, finally resolved The patient was in remission, a second conditioning chemotherapy followed by allo-HSCT (remained on remission 56 days afterward and discharged) 2. Patient 2: a 13-year-old boy with relapsed disease on prior treatment and refractory cytopenia, was included in the study. The patient received 1 × 10^6^/kg BE-CAR7 cells. The patient developed G4 CRS, G1 rash, and G2 upper respiratory infection. G4 hypoxia and pleural effusion BM investigation showed MRD on days 19 and 27 The patient died on day 33 3. Patient 3: a 15-year-old boy with primary mixed-type T-ALL, and relapse on prior two allo-HSCT. The patient received 0.9 × 10^6^/kg BE-CAR7 cells after LD. Transient G2 CRS, G3 rash, and G4 cytopenia developed after treatment. BE-CAR7 cells were detectable on follow-up and the patient was on CR on 28 day, undergone allo-HSCT
NCT04960579 [237] Abstract	2023, USA	Relapsed/refractory MM	BCMA	β2M & TRBC1	Phase I/Ib 3 + 3 dose escalation clinical trial, lymphodepletion by cyclophosphamide and fludarabine at varying dosages, followed by 0.0625 × 10 ^6^ to 15 × 10 ^6^ cells/kg P-BCMA-ALLO1 24 patients at 4 dose levels (22 in arm S, 1 in arm P1 and 1 in arm P2)	Of the 22 patients from arm S completing DLT study, none showed DLT G1 CRS in 3/22 (14%), G1 ICANS in 1/22 (4%). No GVHD observed
NCT04637763 [245] Abstract	2023, USA	A 68-year-old man with stage III DLBCL	CD19	TRAC & PD1	A phase I clinical trial −3 + 3 dose escalation part- case report. Lymphodepletion with cyclophosphamide and fludarabine, followed by 40 × 10^6^ CB-010 cells	A 68-year-old man with stage III DLBCL had received four prior lines. After LD, the patient received 40 × 10^6^ CAR-T cells. The patient showed CR at 28th day No ICANS, GVHD, prolonged cytopenia, or CRS observed The patient remained in CR on 21 months of follow up
NCT04637763 [246] Abstract	2022, USA	A 66-year-old man with follicular lymphoma	CD19	TRAC & PD1	A phase I clinical trial −3 + 3 dose escalation part- case report. Lymphodepletion with cyclophosphamide and fludarabine, followed by 40 × 10^6^ CB-010 cells	A 66-year-old man with FL had received eight prior lines, and CD19 + disease was included. After LD, the patient received 40 × 10^6^ CAR-T cells. The patient showed CR at 28th day No neurotoxicity, GVHD, DLT, or CRS were observed The patient remained in CR on 12 months of follow up
NCT04637763 [152] Abstract	2024, USA	Relapsed/refractory B-cell NHL	CD19	TRAC & PD1	A phase I clinical trial −3 + 3 dose escalation part. Lymphodepletion with cyclophosphamide and fludarabine, followed by 40 × 10^6^, 80 × 10^6^, 120 × 10^6^ CB-010 cells (n = 8, 5, 3, respectively)	1. 11/16 (69%) CR, 15/16 (94%) had an overall response, median time to achieve CR was 28 days. 7 patients achieved CR after 6 months 2. Peak expansion of CAR-T cells occurred at 7–10 days, and B cells were specifically low after 3 months, while T and NK cells recovered 2. No GVHD. G1-2 CRS in 7/16 (44%), G1-2 ICANS in 2/16 (13%), G3-4 ICANS in 2/16 (13%)
NCT03166878 [258] Abstract	2022, China	Relapsed/refractory DLBCL	CD19	β2M & TCR	Phase I clinical trial on patients with relapsed or refractory DLBCL -two case reports. Lymphodepletion with cyclophosphamide and fludarabine, followed by 1.5 × 10^6^/kg and 1.21 × 10^6^/kg UCART19 cells	1. Patient 1: a 63-year-old man with primary progressive DLBCL and several prior lines of therapy and a bulky tumor was included. After CAR-T cell infusion, the patient developed G4 CRS not responsive to therapies. The patient declined all treatments after 21 days 2. Patient 2: a 64-year-old man with DLBCL who had received several prior lines was included. The patient had CRS limited by 1 week. No GVHD or other toxicities observed CAR-T cell expansion occurred rapidly and cells persisted for 1 month, so as B cell depletion The patient had disease progression, 1 month after infusion
NCT04502446 [248]	2025, multi-nationality	Relapsed/refractory peripheral or cutaneous T-cell lymphoma	CD70	TRAC & β2M &CD70	A phase I dose escalation 3 + 3 clinical trial in 22 patients with PTCL and 17 patients with CTCL, LD as fludarabine and cyclophosphamide, followed by 3 × 10^7^, 1 × 10^8^, 3 × 10^8^, 9 × 10^8^ CTX130 cells (n = 4,4,5,26, respectively)	1. 18/39 patients had ORR (46.2%) At DL3-4, DCR was 100% (12/12) in patients with CTCL and 63% in patients with PTCL (12/19), 10/19 (52.6%) had CR or PR in PTCL, 6/12 (50%) had CR or PR in CTCL at DL3-4 2. Median PFS for CTCL was 5.4 months, and 2.5 months for PTCL (DL3-4), Median OS were 10.5 and 7.4 months, respectively 3. In most of the patients CTX130 rapidly increased and were undetectable by 28 days (not different across DL) 4. 14/39 patients had G4 AE (35.9%), including cytopenias in 13/39 (33%) and G4 CRS in 1/39 (2.6%). G3 AE were observed in 18/39 (46%), mostly comprising cytopenias. CRS included G1-2 in 25/39 (64%), G3 in 3/39 (7.7%) and G4 in 1/39 (2.6%). 2 patients had > G2 heart failure (5.2%) 3 patients had both CRS and another serious AE related to CTX130 (G1 neurotoxicity, G2 atrial fibrillation, G3 haemophagocytic lymphohistiocytosis). Notably, CRS incidence did not associate with subsequent infusions Three other patients showing serious AE but no CRS had G2 neurotoxicity, G3 pneumonia and G2 influenza, and G4 neutropenia No GVHD occurred One patient with PTCL on DL4 had G3 haemophagocytic lymphohistiocytosis and G4 CRS after CTX130 infusion and finally led to death (dose-limiting toxicity = 1) Secondary malignancy occurred in 4 patients all in CTCL cases following CTX130 but assessed as unrelated to CAR-T cells
NCT05619861 [234] Case report	2024, China	Relapsed/refractory T-cell ALL	CD7	CD7	LD by fludarabine and cyclophosphamide, followed by first CAR-T cell infusion (5 × 10^5^/kg), second LD by cyclophosphamide with subsequent CAR-T cell injection (1.5 × 10^6^/kg)	A 17-year-old boy, receiving several lines of chemotherapy and one allo-HSCT, participated after the third relapse Following the first infusion, no febrile reaction occurred. MRD was detected as early as day 4 after first CAR-T infusion with an increasing values until second infusion Following the second dose, the patient experienced low-grade CRS & cytopenias. MRD was not detectable on BM on day 110 follow-up. The patient was disease-free until 42 months with no signs of relapse (CR)
NCT05812326 [257] Abstract	2024, China	Advanced breast cancer	MUC1	PD1	Phase I dose escalation −3 + 3- study in 12 patients with recurrent or metastatic MUC1 + BC, CAR-T cells starting at 1–2 × 10^6^/kg up to 0.9–1.8 × 10^7^/kg (1–3 cycles each)	1. All patients received at least 1 cycle of CAR-T cells 5/12 (42%) showed SD 2. CAR-T cell expansion was detected on day 7 3. No serious AE or DLT occurred. Moreover, no AE resulting in drug discontinuation were identified No G3 or higher CRS and no neurotoxicity occurred. G4 lymphopenia occurred in one patient lasting only 1 day. Other AE included changes in liver function tests (in 5 and 7 patients), lymphopenia (4/12), skin rash (5/12) and chills (4/12)
NCT05454241 [251] Abstract	2023, China	Relapsed/refractory T-cell ALL or lymphoblastic lymphoma (LBL)	CD7	CD7 & TCR	A phase II clinical trial of pediatric patients with r/r T-ALL or T-LBL, LD by fludarabine and cyclophosphamide, followed by a single dose of 2 × 10^6^/kg, with subsequent allo-HSCT allowed if preferred by the physician and patient	Five patients, including 4 with T-ALL and a median of 47% blasts, and 1 with T-LBL without any BM blasts but having a large mediastinal mass 1. All 4 T-ALL patients had CR/CRi (4/5), while the patient with the large mediastinal mass had PR. On the median follow-up time of 109 days, 1 patient was MRD negative and on CR, one showed relapse after 49 days. The other three undergone allo-UCBT and were on CR. All children were alive on the follow-up 2. G1-2 CRS detected in 4/5 (80%) of the patients. No GVHD detected
NCT06014073 [244] Abstract	2024, China	Relapsed/refractory B-cell NHL	CD19	TRAC & Power3 (SPPL3)	Phase I dose escalation 3 + 3 study, LD by fludarabine and cyclophosphamide, followed by 1 × 10^6^/kg, 3 × 10^6^/kg, and 10 × 10^6^/kg ET-901 cells (6 patients including 3 at DL1 and 3 at DL2 at the interim analysis)	Four patients with DLBCL and 2 with FL 1. 4/6 of patients had CR (66%) including 1 in DL1 and 3 in DL2, ORR was 6/6 (100%) 2. The peak level of CAR cells were identified at a median of 7 days. All patients showed a second expansion of CAR-T cells along with B cell recovery 3. No DLT were detected. Most common AE of any grade included cytopenias in 6/6 (100%), CRS in 6/6 (100%), infection in 2/6 (33%), ICANS in 2/6 (33%), and GVHD in 1/6 (16%)
NCT04613557 [85] (also in preclinical)	2025, Multi-nationality	Relapsed/refractory MM	BCMA	TCR	A phase I clinical trial −3 + 3 dose escalation. non-myeloablative LD by cyclophosphamide and fludarabine, followed by one dose of 3 × 10^7^, 1 × 10^8^ and 3 × 10^8^ cells (n = 3,3,6, respectively)	1. 3/12 had PR (25%), one from each DL, 9 others had SD 2. CYD-211 expansion occurred at all DL for 3–4 weeks. CAR-T cell persistence negatively correlated with leukocyte re-expansion CAR-T cells at their peak values had significant suppressive effects on cytokines 3. No GVHD, DLT, and CAR-related encephalopathy syndrome was observed 10/12 (83%) patients experienced AE related to CAR-T, including, 5/12 with G3 or more (41.6%) mostly related to abnormal lab results and cytopenias CRS: 1/12 and occurred at DL1, CRS G1 which required hospitalization and resolved
NCT04557436 [231]	2022, UK	Relapsed/refractory B-cell ALL	CD19	CD52 & TRAC	A phase I clinical trial in six children with r/r B-ALL. LD with fludarabine, cyclophosphamide, and alemtuzumab, followed by 0.8 × 10^6^ to 2.0 × 10^6^ CAR19/kg, and allo-HSCT if feasible -if molecular remission occurred at 28 days	All six children had > 99% CD19 + blasts 1. 4 of 6 patients had CR/CRi. While the other two had progressive disease, these four patients remained MRD negative by day 28 and were eligible for allo-HSCT 2. Two patients had G1-2 GVHD, one indistinguishable whether due to allo-HSCT or CAR-T, whereas the other was attributed to allo-HSCT All patients had G1-2 CRS, mostly self-resolved. No G3 or more CRS was identified 3. Allo-HSCT was done in 4/6 of patients. No CAR-T cells were detected post-HSCT Two patients remained on remission and were followed more than 12 and 3 months
ChiCTR1900028573 [176] (also preclinical)	2024, China	Relapsed/refractory MM	BCMA	PD1	A phase I clinical trial, LD by fludarabine and cyclophosphamide, followed by three dose schedule on days 0, 2, and 6 at 0.1, 0.3, and 0.6 × 10^7^ cells/kg	1. 5/7 (71%) of the patients had CR, ORR was 6/7 (85.7%) one patient having PR, the remaining had SD Disease burden decreased early in the 6 responding patient within the first 2 months, concurrent to CAR T cell rise, also correlating with serum BCMA level The six patients were MRD negative at first post-infusion follow-up (day 28) Prolonged response was seen in four patients 2. CRS G1-2 in 4/7 (57%), one patient had G3 CRS No ICANS seen
NCT04438083 [82] (also preclinical)	2024, Multi-nationality	Relapsed RCC	CD70	TRAC & β2M & CD70	A phase I clinical trial −3 + 3 dose escalation- in 16 patients with r/r ccRCC, LD with fludarabine and cyclophosphamide, followed by 3 × 10^7^, 1 × 10^8^, 3 × 10^8^, 9 × 10^8^ CAR-T cells	1. DCR was 13/16 (81%) including 12 SD and 1 CR The patient having CR was disease-free at 3 years 2. Median PFS 2.9 months and median OS was 20.5 months 2. No G3 or higher CRS was observed. 4/16 (25%) had serious AE all being CRS. No ICANS or GVHD. G1-2 CRS seen in 8/16 (50%) No DLT observed
NCT05032599 [235]	2025, China	Relapsed/refractory T-cell ALL	CD5	CD5	A phase I clinical trial in patients with r/r T-ALL: patients with either previous allo-HSCT and receiving donor-derived CAR (cohort A, n = 11), patients without prior allogenic SCT and receiving new CAR (cohort B, n = 5). Some had also received prior CD7 CAR Lymphodepletion by fludarabine and cyclophosphamide, followed by 0.5–1 × 10^6^ (desired dose no DLT and ORR 100%), and 2 × 10^6^ cells/kg (DL2, not reported in this study)	1. All patients had CR or CRi (100%) by day 30. Four patient (all from cohort B, due to risk of second SCT in cohort A not performed) received post-CAR HSCT, and at 14.3 months, 3 were on remission and one died from infection 2. Median OS 4.6 months in cohort A, and not achieved in cohort B IRB decided to halt the trial due to multiple instances of fatal infections in cohort A and OS not reached in cohort B. However, the dose attained was presumed as the desired dose for modified further trials 3. CRS G1-2 was observed in 12/16 (75%) all resolved without tocilizumab CRS 7/11 of cohort A, and 5/5 of cohort B patients ICANS G1-2 observed in 4/16 (25%) of patients including 2/11 of cohort A and 2/5 of cohort B patients 11/16 (69%) experienced G1 GVHD, including 8/11 from cohort A and 3/5 from cohort B
NCT04106076 [215] Abstract	2022, USA	Relapsed/refractory AML	CD123	TRAC	A phase I clinical trial -dose escalation- in patients with CD123 + r/r AML, LD with fludarabine & cyclophosphamide (FC) or FC + atezolizumab (FCA), followed by either of 2.5 × 10^5^, 6.25–15 × 10^5^, or 3.03 × 10^6^ cells/kg (8 patients FC + DL1-3, 8 patients FCA + DL2)	1. ORR was 4/16, with 2 in FC and 2 in FCA arm and all at DL2 FC arm had 1 patient with SD FCA arm had 1 SD and 1 CRi. The patient with SD had more than 90% blast reduction in BM. The one with CRi was identified at day 28, full count recovery after 56 days, and remained MRD negative until 8 months of follow-up 2. Adequate LD was not observed in FC group, and 3/8 had CAR-T cell expansion. FCA group patients had robust LD in all patients for more than 28 days, and 6/8 had CAR-T expansion, correlating with cytokine levels and CRS 3. 4 patients experienced DLT; including, G4 CRS and G3 ICANS in 3 DL2 + and FC, and G5 CRS in 1 patient from FCA arm CRS was observed in 15/16, including 3 with G3 or higher
NCT04825496 [232]	2025, China	Relapsed/refractory B-cell ALL	CD19	IL-6	A phase I clinical trial -dose escalation and expansion- in 17 patients with r/r B-ALL, LD by fludarabine and cyclophosphamide, followed by 1 × 10^6^, 5 × 10^6^, 10 × 10^6^ ssCAR-T cells/kg at dose escalation (n = 3, 6, 1, respectively), and 1 × 10^6^ at dose expansion (n = 7)	1. 2 patients died at day 12 and 41 (prolonged neutropenia) and response evaluation were not applicable. 11/15 of patients had CR/CRi (73%), all 11 were MRD negative at 3 months. Responders included 7/8 at DL1 (88%) and 4/6 (67%) at DL2 ORR at day 28 for 16 evaluable patients was 14/16 (88%) 6 patients had progressive disease 2. Median PFS was 22.2 months for all patients, 11.6 for DL1. Median OS was not reached 6-months PFS was 70%, 67%, and 0%, respectively 3 patients at DL1 remained on remission for more than 18 months. 4 responding patients from DL2 received allo-HSCT 3. No DLT was detected, pharmacokinetics were comparable across the three dose levels. Due to higher ORR at 3 months in DL1 compared to DL2 and DL3 (100% vs 67% and 0%), DL1 was chosen 4. All patients had at least one AE related to CAR-T. No patient died of AE related to CAR-T The most common G3 or more AE were cytopenias CRS in 13/17 (76%): G3 CRS in 3/17 (18%) including 1 from DL1 and 2 from DL2, G1-2 in 10/17 (58%)
NCT04938115 NCT04916860 [252] Abstract	2022, China	Relapsed/refractory T-cell malignancies	CD7	CD7	Report from two clinical trials on patients with T-cell hematologic malignancies, lymphodepletion by fludarabine and cyclophosphamide, followed by single dose from 1.5–5 × 10^5^, 1 × 10^6^, 1 × 10^6^ cells/kg	Patients included T-ALL (n = 10), T-LBL (n = 3), and mixed phenotype leukemia (n = 2) On day 28, all patients achieved CR (100%) with MRD negativity Within a median 253 days, 12 patients received allo-HSCT and were progression-free except one who relapsed and then died One patient left the study on day 35 after relapsing. The other two not receiving allo-HSCT -prior history of HSCT- died from infection (n = 1) and GVHD (n = 1) All patients experienced CRS, including 5/15 with CRS G3, and 10/15 having G1-2 ICANS occurred in 3 patients including 1 with G1 and 2 with G3-4 These complications were controlled with corticosteroid with or without tocilizumab
NCT04823091 [253] Abstract	2022, China	Relapsed/refractory T-cell malignancies	CD7	CD7	A phase I clinical trial in adult patients with r/r T-cell malignancies, receiving anti-CD7 CD7KOCAR-T cells from HLA-matched siblings (allogeneic) or autologous cells (n = 5, 5) Lymphodepletion by fludarabine and cyclophosphamide, followed by CAR-T cells either 1 × 10^6^ or 2 × 10^6^ per kg	10 patients entered including 5 with PTCL, 3 with angioimmunoblastic T-cell lymphoma, and 4 with other T-cell malignancies CR seen in 5/10 (50%) 6 of which reached MRD negativity Of 5 patients with extramedullary disease, 4 had partial removal (80%) G1-2 CRS: 7/10 (70%), G3-4: 1/10 (10%). GVHD G1-2 in 2/10 (20%). No ICANS was seen G4 cytopenia: 10/10 (100%) Comparing the two groups, patients from the allogeneic group had a higher likelihood of developing mild CRS and leukopenia, not associated with greater GVHD nor infection risks Patients from the two group had same ORR but higher CR was seen in allogeneic group (3 vs 2)

TRAC: TCRα subunit constant, RECIST: Response Evaluation Criteria in Solid Tumors, PFS: Progression-free survival, OS: Overall survival, AE: Adverse events, CRS: Cytokine release syndrome, TCR: T-cell receptor, ALL: Acute lymphoblastic leukemia, y/o: Years old, GPC3: Glypican 3, TGFβ: Transforming growth factor beta, DAP10: DNAX-activating protein 10, HPK1: Hematopoietic progenitor kinase 1, scFv: Single-chain fragment variable, PSCA: Prostate stem-cell antigen, PSMA: Prostate-specific membrane antigen, MUC1: Mucin 1, HER2: Human epidermal growth factor receptor 2, EGFR: Epidermal growth factor receptor, TIGIT: T cell immunoreceptor with Ig and ITIM domains, shRNA: Short-hairpin RNA, CLL: Chronic lymphocytic leukemia, ICANS: Immune effector cell-associated neurotoxicity syndrome, ORR: Objective response rate, CR: Complete response, CRi: Complete response with incomplete count recovery, PR: Partial response, NR: No response, NSCLC: Non-small cell lung cancer, SCT: Stem-cell transplant, AML: Acute myeloid leukemia, CLL-1: C-type lectin-like molecule-1, DLBCL: Diffuse large B-cell lymphoma, GBM: Glioblastoma multiforme, ZFN: Zinc-finger nucleases, KFS: Karnofsky performance score, CNS: Central nervous system, CRES: CAR-T-related encephalopathy, ICE: immune effector cell-associated encephalopathy, MRD: Minimal residual disease, MLL: Mixed lineage leukemia, LD: lymphodepletion, FL: follicular lymphoma, HGBL: high-grade B-cell lymphoma, NHL: non-Hodgkin lymphoma, PTCL: peripheral T-cell lymphoma, CTCL: cutaneous T-cell lymphoma, BC: breast cancer, LBL: lymphoblastic lymphoma, r/r: relapsed/refractory, ccRCC: clear cell renal cell carcinoma

Two independent researchers extracted the data of the included studies in these word tables. In case of disagreement between the extracted data, the researchers reviewed the original data again to see if any mistakes were made. If the conflict persisted, they contacted each other to solve the issue. Finally, if they still disagreed, another independent researcher tried to resolve the matter.

### Qualitative synthesis and data presentation

We did not aim to perform a meta-analysis for this study, as we predicted numerous studies containing vast and heterogeneous results on various genes. After data extraction, we planned to better visualize and present the extracted data using the following categorization:

We first divided the studies into animal and human studies. The animal studies were categorized based on the purpose of their gene editing into five overall sections: (1) Produce allogeneic CAR-T cells while avoiding graft-versus host disease (GVHD), (2) increase CAR T cell efficacy, (3) decrease CAR T cell toxicity, (4) limit CAR T cell fratricide, and (5) enabling concurrent therapies. Then, as the reader could now grasp an overview of CAR T cell gene KO/KD utility, we divided the human studies into two categories of hematologic and solid tumors.

### Risk of bias/quality assessment

We utilized the CYRCLE checklist for animal studies [24]. For human studies, we utilized the National Heart, Lung, and Blood Institute (NHLBI)’s study quality assessment tool, which encompasses a large range of study designs, suitable for this systematic review [25].

## Results

**Fig. 1:**
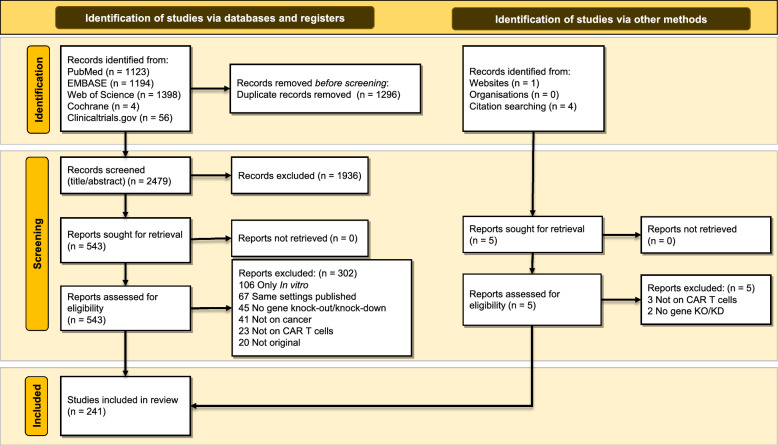
PRISMA flow diagram of the study screening process

The overall search yielded 3780 results, of which 241 records were finally included in this systematic review (Fig. 1). These 241 articles comprised 193 animal and 52 human studies, as four studies had both human and animal subjects.

Genes for an overall 105 proteins were KO/KD in these studies: TCR, HLA-I, TRAC (T cell receptor α constant chain), TRBC (T cell receptor β chain), B2M (β2 microglobulin), CTLA-4, PD-1, TGFBR2 (TGF-β receptor type 2), CD45 (PTPRC), IL-6, IL-6R, LAG3, TIM3, TIGIT, CD70, CD5, CD7, CCR7, MICA, MICB, FOXO1, ITK (IL-2-inducible T cell kinase), HPK1, glucocorticoid receptor, CD58, SPPL3 (Power3), CD52, CD3, PD-1H (VISTA), IL-2, IL2Rα, IL2Rβ, CUL5, B7-H3, ADRB2, PIK3CD (PI3Kδ), CD2, IFN-γ, IFN-γR, CD99, BLIMP-1 (PRDM1), Adenosine 2a receptor (A2AR), CD47, CISH, FAS, PTPN2, RASA2, CBLB, NR4A1 (Nur77), NR4A2 (Nurr1), NR4A3 (Nor1), RGS16, LCK, TNFAIP3 (A20), CSF2 (GM-CSF), EP2 (Prostaglandin E2 receptor 2), EP4 (Prostaglandin E2 receptor 4), CIITA, PVR, DGKα (diacylglycerol kinase alpha), DGKζ (diacylglycerol kinase zeta), MED12, CCNC, SLAMF7 (CSI), PTGR2, PTGR4, BTLA, FKBP12, BCL11B, EGR2, CD247, AAVS1, ZFP36, PSGL-1, SOCS3, TNF, CD83, SHP-1 (PTPN6), SHP-2 (PTPN11), CD38, RFX5, DNMT3A, IL-10R, IDO1, CD97 (ADGRE5), TRIM21, IL-4, IL-4R, ACAT1, CD200R, SNX9, LFA-1α (ITGAL), VLA-4α (ITGA4), MCJ (DnaJC15), HDAC11, NKGD2, BATF, IKZF3, TLE4, IKZF2, TMEM184, EIF5A, GATA3, TET2, and ZC3H12A (Reg-1, Regnase-1).

### Pre-clinical (animal) studies

A total of 193 studies were included for this section. A detailed summary of all the studies is represented in Supplementary Table 1. Genes for an overall 101 proteins were KO/KD in these studies: TCR, HLA-I, TRAC, TRBC, B2M, CTLA-4, PD-1, TGFBR2, CD45, IL-6, IL-6R, LAG3, TIM3, TIGIT, CD70, CD5, CD7, CCR7, MICA, MICB, FOXO1, ITK, CD52, CD3, PD-1H, IL-2, IL2Rα, IL2Rβ, CUL5, B7-H3, ADRB2, PIK3CD, CD2, IFN-γ, IFN-γR, CD99, BLIMP-1, Adenosine 2a receptor, CD47, CISH, FAS, PTPN2, RASA2, CBLB, NR4A1, NR4A2, NR4A3, RGS16, LCK, TNFAIP3, CSF2, EP2, EP4, CIITA, PVR, DGKα, DGKζ, MED12, CCNC, SLAMF7, PTGR2, PTGR4, BTLA, FKBP12, BCL11B, EGR2, CD247, AAVS1, ZFP36, PSGL-1, SOCS3, TNF, CD83, SHP-1, SHP-2, CD38, RFX5, DNMT3A, IL-10R, IDO1, CD97, TRIM21, IL-4, IL-4R, ACAT1, CD200R, SNX9, LFA-1α, VLA-4α, MCJ, HDAC11, NKGD2, BATF, IKZF3, TLE4, IKZF2, TMEM184, EIF5A, GATA3, TET2, and ZC3H12A.

Based on the purpose for gene editing, we categorized these genetic mutations in five sections presented below.

#### Production of allogeneic CAR-T cells while avoiding GVHD

Historically, CAR T cells were produced using the patient’ own T cells (autologous) to avoid precipitating the risk of GVHD when stranger donor T cells attack the recipient’s cells [26]. This approach results in disadvantages in the time and cost of CAR T cell therapy [27], where the patients could not receive readily available “off-the-shelf” CAR T cells.

There were 68 studies mentioning producing allogeneic CAR-T cells with gene KD/KO to limit GVHD [26–92]. TRAC was the most KO gene [28, 29, 31–34, 36, 38–45, 47, 49, 51, 52, 54, 58–83], followed by β2M [26, 30, 31, 35, 41, 42, 45, 49, 52, 58, 71–83, 87, 91, 92]. Only two studies KO TRBC [27, 87], three KO TCRαβ [48, 88, 89], and four studies mentioned T cell receptor KO without mentioning it specifically [50, 55–57]. Moreover, four studies mentioned KO HLA-I gene [55–58]. The studies that stated gene disruption efficiency reported a KO efficiency of 80 to 100%. Many of these studies also used concurrent gene KO/KD to achieve other purposes for their allogeneic CAR T cells, such as improving efficacy or safety.

Β2M was KO concurrently with TRAC in all the studies, but two [91, 92]. In the study by Grauwet et al., KO/KD of Β2M and CIITA on CD19-directed CAR T cells successfully reduced anti-CAR responses with preserved antitumor function, even in cases with prior CAR T cell therapies [91]. Moreover, Menegatti et al. performed gene screen to explore targets for KO to produce persistent allogenic CAR T cells. In their study, they identified superiority of FAS over Β2M due to resistance to natural killer cells while maintaining the anti-tumor efficacy [92].

LCK has been shown by two studies as a target for KO reducing alloreactivity -by reducing T cell activation via T cell receptor. In these studies, LCK KO allogeneic CAR T cells showed higher persistence and tumor control compared to TCR KO cells, indicating their possible roles for further investigation [90, 92].

Two of the studies in this section brought up the concern on the possibility of decreasing CAR T cell efficacy after these gene KOs. The study be Stenger et al., which was the only one that utilized TRBC KO, reported CAR T cells without TRBC KO had better anti-leukemic activity compared to TRBC KO CARs and the mice treated with them had longer survival [27]. Another study by Wang et al. on PD-1 plus TRAC KO CAR T cells for pancreatic cancer mouse models mentioned a possible negative effect of TRAC KO on CAR T cell infiltration in tumoral tissue, leading to tumor growth [53]. However, most of the studies on allogeneic CAR T cells either reported a non-inferior efficacy of these CARs compared to autologous controls [38, 39, 54], or did not report this matter. On the other hand, two studies reported an increased efficacy of TRAC KO anti-CD19 CAR T cells, one a non-significant increased survival in acute lymphoblastic leukemia (ALL) mouse model [29] and another demonstrating less tumor burden and CAR T cell exhaustion in Burkitt’s lymphoma [34].

Therefore, TRAC KO probably will not decrease the CAR T cell efficacy, and the studies on the lower efficacy of allogeneic CAR T cells might be incidental or dependent on the context (e.g. pancreatic cancer in Wang et al. [53]). However, further studies are required to further elucidate this matter.

#### Increasing CAR T cell efficacy

This section entails the highest number of studies, with 127 studies investigating 82 genes [29–31, 41, 46, 49, 51, 53, 55, 59, 64, 68, 72–74, 79, 82, 83, 85, 91, 93–199]. The most common KO/KD gene was PD-1 with 23 studies [30, 31, 49, 53, 64, 100, 104, 105, 108, 109, 111, 112, 116–119, 121, 127, 130, 139, 142, 144, 176, 179]. The studies on PD-1 KO/KD found an increased CAR T cell infiltration into tumor tissue [100, 108], augmented survival and expansion of CAR T cells in the tissue [100, 105, 111, 118, 142], enhanced cytokine production against the tumors (such as IL-2) [100, 108, 111], better CAR T cell cytotoxicity [49, 53, 99, 100, 108, 111, 118, 119, 127, 139, 144, 176], and increased mice survival [31, 64, 99, 100, 104, 108, 109, 116, 117, 119, 139, 142] with PD-1 KO/KD mice compared to the controls.

KO/KD of other immune checkpoints were also reported with promising results, including CTLA-4 [122, 126, 127], PD-1H (VISTA) [99], TIGIT [117, 139, 179], and LAG-3 [96, 139]. CTLA-4 KO [126, 127]/KD [122] resulted in increased CAR T cell cytotoxicity and decreased tumor growth. PD-1H KD [99] & PD-1/TIGIT KO [117] anti-CD19 CAR T cells increased the survival of mice with lymphoma [99, 117] or acute lymphoblastic leukemia (ALL) [117]. Moreover, PD-1 KO together with LAG-3, TIM-3, TIGIT, and TGFbR2 KO showed improved anti-tumor activity and survival compared to PD-1 alone on a colorectal cancer model [139]. LAG-3 KO anti-CD19 CAR T cells also demonstrated a significantly decreased tumor size compared to CAR T cells with no gene KO [96].

A2aR KO/KD resulted in decreased tumor size and enhanced anti-tumor killing abilities of CAR T cells compared to controls in five studies in subjects with breast, colon, pancreatic, cervical, and ovarian carcinoma [102, 107, 114, 120, 184]. Beavis et al. found that combination therapy with anti-PD1 antibodies further increased breast cancer mice survival and A2aR-KD anti-HER CAR T’s anti-tumor ability [120]. Giuffrida et al. reported an increased efficacy of A2aR-KO CAR T cells compared to pharmacological A2aR blockade or A2aR-KD, yet raised the concern on decreased persistence of A2aR-KO CARs in the tumor microenvironment [102]. Soltantoyeh et al. mentioned an increased cytotoxicity and survival outcomes in dual KD TIM-3 and A2aR in mesothelin-directed CAR-T cells in cervical cancer, while single KD cells showed survival concerns on the models [184].

Besides the aforementioned genes, other genes were also KO/KD in the studies to achieve increased efficacy: CD5, CD7, CD70, TCR, HLA-I, DGKα, DGKζ, EIF5A, CIITA, TGFBR2, CUL5, B7-H3, ADRB2, BLIMP1, PTPN2, RASA2, CBLB, Nr4a1 (Nur77), Nr4a2 (Nurr1), Nr4a3 (Nor1), LCK, TNFAIP3, TIM-3, MICA, MICB, FOXO1, PVR, EP2, EP4, MED12, CCNC, ITK, FAS, BTLA, CCR7, FKBP12, TRBC, EGR2, TET2, CD47, BATF, MED12, ZFP36, PSGL-1, SOCS3, SHP-1, SHP-2, DNMT3A, IL-10R, IL-6R, CD38, CD52, IDO1, CD97, TRIM21, IL-4, IL-4R, ACAT1, CD200R, SNX9, ITGAL, ITGA4, TLE4, IKZF2, EIF5A, TMEM184B, Reg-1, CD45, DNAJC15, HDAC11, NKG2D, IL-2, IL-2Rb, IKZF3. Almost all of these KO/KDs yielded some form of increased anti-tumor activity in different settings, with details of each study presented in Supplementary Table 1.

#### Decreasing CAR T cell toxicity

A total of nine studies investigated the effects of several genes, including, IL-6 [93], GM-CSF [40, 94], and IFN-γ [200–202] KO/KD on reducing the rates of CRS, GVHD, neurotoxicity, and immunotoxicity of CAR T cells. Other genes that were KO/KD for this purpose were PI3Kδ [203], TCR [204], RGS16, PD-1 and NR4A2 [205]. Notably, An et al. did not find any improvements in CRS by KD of PI3Kδ, while the other studies showed significant benefits.

IL-6 KD in anti-CD19 CAR T cells against Burkitt’s Lymphoma resulted in no objective neurotoxicity, immunotoxicity, or tumorigenicity, while retaining the anti-tumor cytotoxicity and production of other cytokines. IL-6 KD even decreased renal, hepatic, and splenic involvements and increased mice survival [93].

In the study by Benton et al., neoantigenic mutant KRAS was targeted by CAR-T cells in models of metastatic lung, pancreatic, and renal cell cancer, showing high relapses. Adding inducible IL-12 resulted in significantly higher antitumor activity and maintained toxicity profiles when combined with the deletion of TCR [204].

GM-CSF KO/KD resulted in no diminished efficacy [40, 94], and in fact, decreased tumor volume and increased survival [94]. However, both studies had insufficient data on safety. Sterner et al. reported their safety data using anti-GM-CSF antibody Lenzilumab but not GM-CSF KD, which showed decreased CRS and neuroinflammation [94]. The abstract by Hu et al. also sufficed to conclude that GM-CSF plus TRAC anti-CD19 CAR T cell population gradually decreased after tumor eradication, possibly showing that they will not remain to cause significant side effects [40].

IFN-γ KO/KD also did not decrease the efficacy of anti-CD19 CAR Ts against leukemia/lymphoma models, while decreased the levels of some inflammatory cytokines (MCP-1, MIP-1β, IL-6, TNF-α, and IP-10) and macrophage activity markers (CD80 and CD86) [200–202].

#### Limiting CAR T cell fratricide

Fratricide occurs when CAR T cells attack and destroy each other instead. The prototype of this challenge arises while treating T cell malignancies. However, this phenomenon may also happen in other cancers if the target antigen is partially present on CAR T cells.

Twenty-eight studies tried to overcome this barrier [32, 35, 36, 44, 46, 48, 63, 81, 87, 134, 138, 150, 156, 177, 178, 180, 181, 192, 198, 206–213]. Twelve studies were on T cell malignancies; they KO CD2 [208], CD3 [35, 48], CD5 [138, 150, 177, 178, 198, 206], or CD7 [32, 35, 138, 156, 207] and simultaneously targeted these antigens on tumor cells. Other studies in this section were designed against non-T cell malignancies, of which three utilized SLAMF7 (CSI) plus TRAC KO anti-SLAMF7 CAR T cells against multiple myeloma (MM) [36, 44, 63]. Interestingly, one of the studies used CD70 KO anti-CD70 CAR Ts against renal cell carcinoma (RCC) [46] (Supplementary Table 1).

#### Enabling concurrent therapies

Six studies in this section all utilized CD52 KO to enable the administration of anti-CD52 antibody alemtuzumab for lymphodepletion plus the conventional fludarabine and cyclophosphamide (FC). These studies achieved their goals without reporting significant harm from alemtuzumab to the CAR T cells [35, 37, 47, 51, 52, 62].

It would be appropriate to mention a study in the human section that knocked out glucocorticoid receptor gene in anti-IL13Rα2 CAR T cells against glioblastoma, to enable the use of high-dose dexamethasone without fearing of its detrimental effects on CAR Ts; although the overall treatment design did not produce promising results [214].

### Clinical human studies

A total of 52 studies were eligible for this section and their detailed summary is provided in Table 1. A total of 15 genes were KO/KD in clinical studies: TRAC, β2M, HLA I, PD-1, CD52, IL-6, GM-CSF, CD5, CD7, CD70, MICA, MICB, TET, SPPL3 (Power3), and glucocorticoid receptor. Most of the studies were phase I or I/II clinical trials, and we had no available data for phase III clinical trials.

As the overall categorization was discussed in pre-clinical studies and the same also applies to the studies on human subjects, we decided to divide the clinical studies based on the type of malignancy to hematologic malignancies and solid tumors.

#### Hematologic malignancies

A total of 45 studies were conducted on patients with hematologic malignancies [40, 71, 85, 123, 152, 176, 215–253], divided into three groups: (1) studies on leukemia or MM, (2) studies on lymphoma, or 3) Mixed studies.

(1) **Leukemia**: A total of 22 studies were related to leukemia [123, 215–235].

**Myeloid leukemia**: Three studies reported patients with myeloid leukemia [123, 215, 216].

A phase I trial of relapsed/refractory AML used CD123 targeted CAR T cells with TRAC KO with either fludarabine and cyclophosphamide (FC) or FC plus atezolizumab (FCA) for lymphodepletion. FC group had inadequate lymphodepletion, while FCA group had robust lymphodepletion. Overall, ORR was 4/16, 2 in FC and 2 in FCA arms, four patients had dose-limiting toxicity, and three patients had G3 or above CRS [215].

Ma et al. reported two 28 years-old males with acute myelogenous leukemia (AML) relapse after anti-CD38 CAR T cells and hematopoietic stem cell transplantation (HP-SCT). In both patients, PD-1 KO anti-CLL-1 CAR T cells led to complete molecular response with incomplete hematologic recovery (CRi) at day 28 and are at complete remission on 8 & 3 months. No grade 3 or higher adverse events (≥ G3 AEs) were seen in the patients [216].

The other study was a phase I clinical trial of MICA & MICB KO anti-NKG2D CAR T cells in six patients with relapsed/refractory myelodysplastic syndrome (MDS)/AML. The authors reported no “dose-limiting toxicity”, but no efficacy data were present at the time [123].

**Lymphoid leukemia**: This section comprised 16 studies on B-cell ALL [217–232], two of them having CNS B-ALL [219, 222], and three studies on T-cell ALL [233–235]. The B-ALL studies either applied a TRAC plus CD52 [218, 220, 221, 223, 225, 226, 231] or an IL-6 KO/KD [217, 219, 222, 224, 227–230, 232] strategy, using anti-CD19 [217, 219–224, 226–232] or anti-CD22 [218, 223, 225, 230], and all reached promising results. T-ALL studies were either CD7 KO anti-CD7 [233, 234] or CD5 KO anti-CD5 CAR T cells [235].

Benjamin et al. reported 12/25 CR/CRi rate for relapsed/refractory B-ALL patients receiving TRAC + CD52 KO anti-CD19 (9/12 MRD negative), all from the patients in the group that received alemtuzumab [226]. Median duration of response and OS were 7.4 and 13.4 months. This study reported considerable toxicity leading to three treatment-related deaths, six G3-4 CRS, two GVHD, and one G4 neurotoxicity. Two other articles on phase I clinical trials on TRAC + CD52 KO anti-CD19 [220, 223] or anti-CD22 [223] CAR T cells had 14/21 (67%) [220] and 5/6 [223] of the patients achieving CR, with 10 patients proceeding to HP-SCT in the former [220]. Notably, only four patients in the former study did not receive alemtuzumab, and none achieved CR. Three (14%) of the study’s patients had G3-4 CRS, and two patients died of treatment complications (CRS + sepsis, and persistent cytopenia + pulmonary hemorrhage) [220]. BALLI-01 phase I trial included 11 patients with CD22 + B-ALL and 70% or more blast count in the bone marrow. Preliminary results from TRAC + CD52 KO anti-CD22 CAR Ts led to blast reduction in two patients, and only one patient suffered from G3 AEs [218]. Updated results from BALLI-01 reported three patients, from which two responded and a CD22-low patient was resistant since the treatment began. A patient died from treatment-related sepsis [225]. Baptista et al. reported 5/6 CR/CRi in patients receiving TRAC + CD52 KO anti-CD19 or anti-CD22 CAR T cells, with no GVHD, dose-limiting toxicity, or neurotoxicity [230]. In a cohort of six pediatric patients with CD19 + blast > 99% relapsed/refractory B-ALL, CD19-targeting TRAC + CD52 KO CAR Ts yielded 4/6 CR/CRi, two of them in remission by the time of report at 3 and 12 months. The only patients who required intensive care unit was a case of G4 ICANS [231]. The last study [221] that utilized TRAC + CD52 was a detailed case report of two patients from the same clinical trial as the study from Benjamin et al. [220] (patients’ details presented in Table 1).

The two studies on CNS B-ALLs both utilized IL-6 KD [222]/KO [219] anti-CD19 CAR T cells. Mao et al. study reported an ORR of 11/12 (91.7%), CR = 10 (83.3%), and no G3 or higher AEs [219]. Chen et al. also reported a CR in three of four patients with no significant AEs during treatment, yet a patients died 10 months after CAR T treatment with GVHD and pneumonia [222].

The last studies on relapsed/refractory B-ALL investigated IL-6 KD anti-CD19. A study of 47 IL-6 KD (ssCAR-19) vs 40 IL-6 wildtype (cCAR-19) anti-CD19 CAR T cell therapy reported similar PFS and response rates in the groups (CR/CRi of 91.5% vs 85%, median PFS of 14.17 vs 15.33). However, ssCAR-19 had a better safety profile with 7/47 (15%) G3-4 CRS compared with 15/40 (37.5%) G3-4 CRS and ICANS G3 in 2/40 (5%) in cCAR-19 group [227]. Similar results were reported in a post-hoc analysis comparing the above CAR Ts [228]. In a phase II trial of 61 patients, ssCAR-19 had medically manageable CRS profile (81%, including 28% G3-4) and more severe CRS correlated with better CAR T expansion [229]. At 36 months, OS rate was 54%. Another study reported a 11/15 CR at 3 months, with median PFS and OS of 22.2 months and not reached. The most common G3 or above AEs were cytopenias, all of which resolved [232]. In Kang et al., all 13 patients treated with CAR T cells with or w/o IL-6 KD achieved CR, but G3 or higher CRS was lower in IL-6 KD CARs (3/7 (42.8%) vs. 5/6 (83.3%) [217]. The other was a case report of a 29 years-old male with testicular and skin relapse of B-ALL, who is still in remission 2 years and one months after treatment with IL-6 KD anti-CD19 CAR T [224].

Finally, three studies evaluated relapsed/refractory T-ALL. Three cases of childhood T-ALL were treated with anti-CD7 CAR T cells with KO for CD7, TRBC1, TRBC2, and CD52. Two patients went into remission and received allo-HSCT, while the third patient had G4 hypoxia and pleural effusion positive for aspergillus niger and eventually died at day 33 [233]. In another case report, a 17-year-old patient who relapsed after several prior lines of therapy, including one allo-HSCT, received CD7 KO anti-CD7 CAR T cell therapy, responded completely, and is now in remission 42 months after treatment [234]. In the last study, 16/16 CR/CRi was observed in patients receiving CD5 KO anti-CD5 CARs, but the trial was halted due to multiple fatal infections in the cohort of patients previously treated with allo-HSCT [235].

(2) **MM:** Four studies were conducted in the setting of relapsed/refractory MM, all targeting BCMA [85, 176, 236, 237].

A phase I clinical trial on 42 patients with relapsed/refractory MM who received at least three prior lines of therapy administered TRAC + CD52 KO anti-BCMA (B cell maturation antigen) to enable allogeneic CAR Ts who are able to tolerate alemtuzumab plus FC lymphodepletion. ORR was 61.5% with 38.5% having very good partial response or higher. ≥ G3 CRS occurred in one patient and ≥ G3 infection was reported in 12.8% of the patients, with two dying of severe infection [236]. Another trial of 12 patients receiving anti-BCMA CAR Ts with TCR KO demonstrated nine SD and three PR, and no patients developed GVHD, dose-limiting toxicities, CAR-related encephalopathy, or G3 + CRS [85]. Ouyang et al. also showed 5/7 (71%) CR and 6/7 ORR in their PD-1 KO anti-BCMA CAR T therapy. Four patients had prolonged response and a patient relapsed at 19.2 months. No ICANS was seen, and a patient had G3 CRS managed with tocilizumab and dexamethasone [176].

Finally, β2M and TRCBC1 KO anti-BCMA CAR T cells demonstrated no dose-limiting toxicities in a phase I/Ib dose-escalating trial [237].

(3) **Lymphoma**: A total of 12 studies investigated B-cell lymphoma [152, 238–248]; four on non-Hodgkin lymphoma (NHL) in general [152, 242–244], five on diffuse large B-cell lymphoma (DLBCL) [238, 239, 241, 245, 247], two on follicular lymphoma [239, 246], one on chronic lymphocytic lymphoma (CLL) [240], and one on T cell lymphomas [peripheral T cell lymphoma (PTCL) and cutaneous T cells lymphoma (CTCL)] [248].

All the studies on relapsed/refractory NHL used CD19-targeted CAR T cell therapy. Three of these CD19 CAR T cell studies [152, 242, 243] utilized PD-1 KO designs with [152] or without [242, 243] TRAC KO, the other one used SPPL3 plus TRAC KO [244]. Hu et al. reported 21/21 ORR, 17/21 CR, and median PFS of 19.5 months (13/17 DLBCL CR, median PFS = 18.2 months). G3 or above AEs were manageable cytopenias, no dose-limiting toxicity was seen, and two patients had infections with one attributable to B cell aplasia [242]. Later follow-up of this study showed 7/21 maintaining CR at 39 months [243]. The PD-1 plus TRAC KO anti-CD19 CAR T study demonstrated 15/16 overall response, 11/16 CR, no GVHD, 2/16 G3-4 ICANS, and prevalent G3 or above cytopenia (G3 or above: 9/16 neutropenia, 11/16 thrombocytopeia, and 8/16 anemia) [152]. The SPP3 plus TRAC KO study also yielded promising results, with an ORR of 6/6 and CR of 4/6 with no dose-limiting toxicities [244].

Two clinical trials on DLBCL used CD52 + TRAC KO anti-CD19 CAR T cells [238, 239]. The studies reported promising ORRs. Lekakis et al. reported an ORR of 50% (CR = 50%) that could be increased to 66.7% with consolidation therapy [238]. Neelapu et al. also reported an ORR = 61.5% with CR = 45.2% [239]. Concerning both trials combined, only one patient (either DLBCL or follicular lymphoma) died of aspergillus pneumonia [239], and no dose-limiting toxicity or other G3 or higher GVHD or ICANS were reported. Two studies used β2M + TRAC KO anti-CD19 CAR T cells for DLBCL [241, 247]. A phase I trial reported ORR of 18/27 (66%), CR of 11/27 (41%), CR at 6 months of 5/27 (19%), and two CR at one year. Seven treatment-related AEs were observed, two had G3 or higher ICANS, and no patient had GVHD [247]. A case report of two patients saw one of them refusing further treatment on day 21 due to G4 CRS, pancytopenia and skin reactions, while the other saw disease progression one month after treatment initiation [241]. Finally, in a case report, a 68-year-old DLBCL patient treated with four previous lines of therapy remain in CR at 21 months follow-up with no ICANS, GVHD, or CRS when treated with CD19-targeting CAR T cells bearing TRAC and PD-1 KO [245].

Response rates seems to be higher for follicular lymphoma compared to DLBCL, as the aforementioned trial reported an ORR = 82.6% and CR = 52.2% for patients with follicular lymphoma [239]. A 66-year-old man previously treated with eight prior line for follicular lymphoma received an anti-CD19 TRAC/PD-1 DKO CAR T cells that remained in CR on 12 months of follow-up without dose-limiting toxicity, GVHD, CRS, or neurotoxicity [246].

In a case report of a 78-year-old patient treated with anti-CD19 CAR T cell for CLL, the patient had an incidental TET-2 (c.5635C mutation) and shows disease control and CAR T cell detection 4.2 years after treatment [240].

Finally, a phase I trial recruited 39 patients with CTCL and PTCL receiving anti-CD70 CAR T cells with CD70, TRAC, and β2M KO. Disease control rates were 12/12 for CTCL (6/12 CR or PR) and 12/19 for PTCL (10/19 CR or PR). Median PFS and OS were 5.4 and 10.5 months for CTCL and 2.5 and 7.4 months for PTCL. No GVHD was observed. G4 AEs were observed in 14/39 (13 cytopenia, one CRS) and G3 AEs were observed in 18/39 (46%).

(4) **Mixed studies:** Seven studies evaluated patient populations comprising mixed hematologic malignancies [40, 71, 249–253].

A large study recruited patients from multiple nations in four cohorts receiving CD19 targeted CAR Ts with TRAC and β2M KO for relapsed/refractory B cell malignancies (A-C: NHL, D: B-ALL) and was terminated early in phase II [249]. ORR was observed in 47/82 patients with the highest median PFS seen in cohort C with only 2.92 months. Serious AEs were reported in 32/90 patients.

Another study on PD-1 KD CD19 targeted CAR T cells against relapsed/refractory B cell malignancies reported a 6/16 (37.5%) CR (3/4 in B-ALL, 2/8 in DLBCL, 1/2 in follicular lymphoma), and the three lymphoma responders did not relapse in the follow-up period [250]. The study reported no G3 or above CRS or treatment-related deaths.

An interesting study compared UCAR-T (TRAC and β2M KO) with n-UCAR-T (TRAC HLA-A, and HLA-B KO) CD19-targeting CAR T cells for B-cell malignancies [71]. Despite maintaining an expansion and antitumor response in in vitro and in vivo settings, UCAR-T cells did not expand in the patient settings and yielded no antitumor response (0/6 responders). On the other hand, 3/3 n-UCAR-T cell receivers had CRi with no GVHD or neurotoxicity (2/3 had G2 CRS). This study highlighted an important concept that HLA A-B KO could outperform a B2M KO CAR T in the clinical settings.

The study by Hu et al. also analyzed 11 patients who received anti-CD19 or anti-BCMA CAR T cells [40]. Three patients (two MM and one non-Hodgkin lymphoma (NHL)) received TCR + GM-CSF KO CARs and eight (five ALL, one CLL, one DLBCL, one MM) received CARs without gene KO/KD. Ten patients had CR except the NHL patient, and only three patients in non-gene-edited group had G3 CRS.

Finally, three studies specifically focused on patients with T cell leukemia and lymphoma with promising results [251–253]. One study was on pediatric patients [251], one adults [253], and one mixed [252]. All these studies used CD7 KO CD7-targeted CAR T cells, with TCR KO when autologous cells were attempted. Most of the patients in these studies developed CR/CRi at some point in the study (4/5 [251], 15/15 [252], and 5/10 [253]) with several others also responding to the treatments. AEs reported by Liu et al. were mostly mild and manageable and resulted in no serious AEs [251]. Yang et al. reported 5/15 G3 CRS and 2 G3-4 ICANS controlled with medical management [252]. In the final study, G4 cytopenia were observed in 10/10, alongside G3-4 CRS in 1/10, no ICANS, multiple infections in 6/10, and one death due to fungal pneumonia [253].

#### Solid tumors

Seven studies were present in this Sect. [53, 82, 214, 254–257]. These studies were conducted on mesothelin-positive solid tumors [53, 254], lung cancer [255], glioblastoma [214], esophageal cancer [256], clear cell renal cell carcinoma (ccRCC) [82], and breast cancer [257].

**Mesothelin-positive solid tumors:** Wang et al. conducted both phase I clinical trials in this group [53, 254]. They studied PD-1 KO anti-mesothelin CAR T cells in nine patients (six pancreatic, two ovarian, and one colorectal carcinomas) [53], as well as TRAC + PD-1 KO anti-mesothelin CARs in 15 (six pancreatic cancer, three biliary carcinoma, and six other) [254]. None of the patients developed G3 AEs or dose-limiting toxicity. However, the efficacy results were not as promising. Best response was partial response in two of seven evaluable patients in the first study with PFS = 160 days [53], and stable disease in the second study (first 3–4 weeks: 7 patients, with PFS = 7.1 weeks, OS = 4.9 months; 8–12 weeks: 2 patients) [254].

**Lung cancer:** A phase I trial exists that studies six patients with stage IIIB-IV non-small-cell lung cancer (NSCLC) treated with PD-1 KO anti-MUC-1 CAR T cells [255]. Two patients had decreased tumor size at 4 weeks with limited effects on metastases. No CRS was observed.

**Glioblastoma:** This study was mentioned above in the “enabling concurrent therapy” section. The researchers administered intratumoral glucocorticoid receptor KO anti-IL13Rα2 CAR T cells plus systemic glucocorticoids and aldesleukin in six patients with progressive/recurrent grade III or IV glioblastoma [214]. The efficacy data was not promising. Only two patients had evidence of tumor necrosis at 4 weeks in MRI/PET. The median OS was 2.9 months, with the highest being 11.9 months (in a patient without evidence of tumor necrosis at 4 weeks after treatment). The patients did not experience G3 or higher AEs or dose-limiting toxicity.

**Esophageal cancer**: This trial was completed on nine patients with advanced (stage IIIB/IV) esophageal cancer, four patients receiving a single and five receiving multiple doses of CAR T cells targeting MUC1 with PD-1 KO [256]. Six patients had stable disease, three had progressive disease, all the patients had symptom improvement after CAR T cell infusion, and two patients reached an OS of 24 months. The study reported no G3 or higher AEs in the patients.

**ccRCC**: The study reported a disease control rate of 81.3% in patients with ccRCC receiving CD70-directed CAR T cells with disrupted TRAC, β2M, and CD70 genes. PFS and OS were 2.9 and 20.5 months. One exceptional responder remained in a durable complete response at three years. No dose-limiting toxicity or G3 or higher AEs were observed.

**Breast cancer**: Twelve patients received PD-1 KO anti-MUC1 CAR T cells for MUC1-positive advanced breast cancer [257]. Five of the patients achieved stable disease and no G3 or higher CRS or neurotoxicity or dose-limiting toxicities were observed. The only observed G3 or above AEs was a case of G4 lymphopenia lasting a day.

### Risk of bias/quality assessment

In a few studies, an important encountered issue was the inability to examine the quality in several categories due to the unavailability of adequate explanations. These not reported (NR) status was mostly due to the fact that several of the studies were abstracts and some lacked adequate explanations. Furthermore, a considerable number of issues arose from the low number of sample size, including the lack of adequate sample size for a confident result and the inability of the researchers to report adequate statistical analyses including p-values, confidence intervals, etc. Furthermore, all the trials were phase I or II, with most of them having a single arm. Therefore, almost all the trials had no randomized control group, did not have blinding, or did not report data on randomization/blinding (Supplementary Table 3).For the in vivo section, most of the studies did not report adequate information to detect the biases in the checklist (Supplementary Table 2). This might be because the studies were mostly either abstracts or were reported alongside several in vitro experiments, so the authors could not go in the details of the in vivo experiments comprehensively. Therefore, a detailed quality assessment for the in vivo experiments could not be made.

## Discussion

This is the first systematic review that widely reviews the effects of gene KO/KD on the outcomes of CAR T cell therapy in human and animal studies. We categorized the positive effects of gene KO/KD into five categories of (1) Producing allogeneic CAR-T cells and avoid GVHD, (2) increasing CAR T cell efficacy, (3) decreasing CAR T cell toxicity, (4) limiting CAR T cell fratricide, and (5) enabling concurrent therapies. We also reviewed the human studies in two categories of hematological malignancies and solid tumors, and understood that hematological malignancies were much more frequently studied than solid tumors, and produced promising results and favorable outcomes compared to solid tumors. Only four solid tumor studies in human subjects were completed, all were phase I or I/II clinical trials with small sample sizes, and none of them produced significant response rates or increased survival.

Only few comprehensive reviews exist in this field. Maganti et al.’s meta-analysis only included 11 preclinical studies that utilized CRISPR/Cas9. Similar to this study, they reported the ability to increase the anti-tumor efficacy of CAR T cells and produce allogeneic CAR T cells without increasing the rate of side effects [259]. Another review article discussed the approach of creating universal/allogeneic CAR T cells. They concluded that allogeneic CAR T cells could be successfully produced using TCR and HLA type I gene KO [260], the same to this article using TRAC, TRBC, and β2M genes KO.

Shadbad et al. systematically reviewed studies on PD-1 gene editing or PD-1 antibody blockade in CAR T cell therapy against glioblastoma. Similar to this study, they found that PD-1 KO/KD or blockade increased CAR T cell efficacy [16]. Zhang et al. systematically reviewed the effects of gene-editing techniques on interleukin genes in CAR T cells [14]. Most of the included articles in this systematic review were related to interleukin gene overexpression techniques, and unlike this systematic review, they reported no studies on KO/KD of cytokine genes, such as IL-6 and GM-CSF.

Bonini et al. reviewed the effects of gene editing strategies in engineered T cells. They categorized the benefits of these gene editing as overcoming therapeutic resistance, decreasing undesired AEs profile, increasing persistence and anti-tumor activity of T cells, and resisting immunosuppressive tumor phenotype [261]. The most relevant review to this article is a narrative review from our group. Mirzaei et al. reported several genes that could help produce allogeneic CAR T cells, increase CAR T cell efficacy, decrease AEs, and enable concurrent use of other therapies, such as TRAC, β2M, PD-1, TIGIT, TIM-3, CTLA-4, CD52, glucocorticoid receptor, etc. Despite its comprehensive nature, the mentioned study was not a systematic review and was conducted in 2018, therefore did not comprise numerous studies that we included in this systematic review [23].

Despite the considerable advances in the field of gene-edited CAR T cells, this study highlighted several remaining challenges in this area. The techniques are yet to be integrated into clinical practice, even for hematological malignancies. Although most of the early phase clinical trials were successful in the settings of hematological malignancies, there is a need to conduct phase III clinical trials and utilize the positive results in the routine clinical practice. One future obstacle for large-scale use of gene-edited CAR T cells will be their high costs, which novel strategies, including the development of TRAC/β2M KO CARs, should address this matter. Despite the great efforts made, the situation is currently less hopeful for the solid tumors. Many high-prevalence solid tumors lack the results of earlier phase clinical trials in humans, while the few published studies on the other tumors failed to demonstrate considerable benefits. The path for the solid tumors requires more extensive efforts, and researchers should conduct translational studies to find the optimal genes and settings for each solid tumor, perhaps considering the list of genes we discovered in this systematic review.

Besides these challenges in the clinical settings, there were lack of evidence in several preclinical aspects. For instance, most of the genes were examined by one or two studies, while their potentials could be investigated further. Several categories summoned few studies and may require further research, including decreasing the adverse events and enabling concurrent therapies. Last but not least, few studies utilized concurrent gene editing to achieve several goals togethers. These studies were mostly those that developed allogeneic CAR T cells by TRAC ± B2M KO and used another gene editing to achieve another positive outcome (i.e. increased efficacy, decreased AEs, etc.). However, few studies utilized gene editings that enjoy the benefits from more than one category, and we encourage such approach in the future studies.

Finally, while our study did not aim to address this issue, it is important to note the technical challenges related to CAR T cell engineering to enhance the translational efforts bringing this therapy to the clinic. Gene editing efficiency is a challenge, as it remains inconsistent across studies that result in different KO rates depending on the CRISPR design, target locus, and variability of the donor. This difference can potentially induce a functional heterogeneity among the products [262]. Furthermore, while we have powerful genome editing tools such as the CRISPR/Cas9 system, they can still introduce off-target mutations potentially disturbing unwanted genes and pathways. Extensive genomic validation can help screen for these possible off-target events before clinical translation [4, 263]. Manufacturing and clinical scalability is another challenge, even for the off-the-shelf allogeneic CAR T cells. Process cost, complexity, and the added time required for multiplex editing as well as the duration required for regulatory approvals are just some of the issues faced in this setting. Ongoing efforts are aimed at reducing the burden of these challenges (264).

Although this study provides a comprehensive, systematic, and novel review of literature, it has several limitations. First, due to the extensive number of KO/KD genes, their different purposes, and significant heterogeneity of studies and their aims, we did not aim to conduct a meta-analysis. Second, we also included abstracts that obviously were not peer-reviewed and they could not report all the findings of a specific study. Nevertheless, we utilized this strategy to avoid missing numerous valuable and update data. Finally, some parts of the study lacked significant number of studies. This deficiency was most obvious in human studies on solid tumors.

## Conclusions

This systematic review demonstrated that genetic editing using KO/KD strategies could provide substantial benefits in at least five specific areas: (1) Producing allogeneic CAR-T cells and avoid GVHD, (2) increasing CAR T cell efficacy, (3) decreasing CAR T cell toxicity, (4) limiting CAR T cell fratricide, and (5) enabling concurrent therapies. We found numerous genes that their KO/KD produced any of the aforementioned advantages, and future researchers may use them to achieve their goals. Unfortunately, human studies on solid tumors were fewer and produced less promising results compared to hematologic malignancies, uncovering the complex nature of these tumors and their microenvironment and the need for more complex methods to overcome this barrier.

## Supplementary Information

Additional file 1.

## Data Availability

No original data were reproduced in this study to be declared. Summary of the extracted data for all the included studies are included in the study tables. For more detailed extracted data of each study, it is available upon request from the corresponding author.

## Abbreviations

CAR: Chimeric antigen receptor
KO: Knock-out
KD: Knock-down
PROSPERO: Prospective register of systematic reviews
PRISMA: Preferred reporting items for systematic reviews and meta-analyses
GVHD: Graft-versus-host disease
TRAC: TCRα subunit constant
RECIST: Response evaluation criteria in solid tumors
PFS: Progression-free survival
OS: Overall survival
AE: Adverse events
CRS: Cytokine release syndrome
TCR: T-cell receptor
ALL: Acute lymphoblastic leukemia
y/o: Years old
GPC3: Glypican 3
TGFβ: Transforming growth factor beta
DAP10: DNAX-activating protein 10
HPK1: Hematopoietic progenitor kinase 1
scFv: Single-chain fragment variable
PSCA: Prostate stem-cell antigen
PSMA: Prostate-specific membrane antigen
MUC1: Mucin 1
HER2: Human epidermal growth factor receptor 2
EGFR: Epidermal growth factor receptor
TIGIT: T cell immunoreceptor with Ig and ITIM domains
shRNA: Short-hairpin RNA
CLL: Chronic lymphocytic leukemia
ICANS: Immune effector cell-associated neurotoxicity syndrome
ORR: Objective response rate
CR: Complete response
PR: Partial response
NR: No response
NSCLC: Non-small cell lung cancer
SCT: Stem-cell transplant
AML: Acute myeloid leukemia
CLL-1: C-type lectin-like molecule-1
DLBCL: Diffuse large B-cell lymphoma
GBM: Glioblastoma multiforme
ZFN: Zinc-finger nucleases
KFS: Karnofsky performance score
CNS: Central nervous system
CRES: CAR-T-related encephalopathy
ICE: Immune effector cell-associated encephalopathy
MRD: Minimal residual disease
MLL: Mixed lineage leukemia
