# Supplementary material for: Improving CAR T cell therapy against malignancies through gene knock-down/out strategies: a systematic review
Source: Cancer Cell Int. 2025 Dec 7;26:8. doi: 10.1186/s12935-025-04090-5 (PMC12797449; doi:10.1186/s12935-025-04090-5)
Supplement: Supplementary file 1 — Additional file 1. [file 12935_2025_4090_MOESM1_ESM.docx]

Supplementary material

**The impacts of knocking-down/out CAR T cell genes to enhance their therapeutic efficacy against malignancies: A systematic review**

Search Strategy Page 2

Supplementary Table 1 Page 4

Supplementary Table 2 Page 28

Supplementary Table 3 Page 31

References Page 32

Date of search (for all databases): July 20, 2025

1. **Search Strategy**
   1. **MEDLINE (PubMed interface)**

| #1 | CAR T*[Title/Abstract]) OR (Chimeric antigen receptor*[Title/Abstract])) OR (CAR Transduced T[Title/Abstract] |
| --- | --- |
| #2 | knock-out[Title/Abstract]) OR (knockout[Title/Abstract])) OR (knock-down[Title/Abstract])) OR (knockdown[Title/Abstract])) OR (Gene edit*[Title/Abstract])) OR (Genome edit*[Title/Abstract])) OR (CRISPR*[Title/Abstract])) OR (Clustered Regularly Interspaced Short Palindromic Repeats*[Title/Abstract])) OR (TALEN*[Title/Abstract])) OR (Transcription activator-like effector nuclease*[Title/Abstract])) OR (ZFN[Title/Abstract])) OR (Zinc-finger nuclease*[Title/Abstract])) OR (shRNA*[Title/Abstract])) OR (Short hairpin RNA*[Title/Abstract])) OR (Short hairpin ribonucleic acid*[Title/Abstract])) OR (Homing endonuclease*[Title/Abstract])) OR (Meganuclease*[Title/Abstract])) |
| #3 | **#1 AND #2**  (((CAR T*[Title/Abstract]) OR (Chimeric antigen receptor*[Title/Abstract])) OR (CAR Transduced T[Title/Abstract])) AND (((((((((((((((((knock-out[Title/Abstract]) OR (knockout[Title/Abstract])) OR (knock-down[Title/Abstract])) OR (knockdown[Title/Abstract])) OR (Gene edit*[Title/Abstract])) OR (Genome edit*[Title/Abstract])) OR (CRISPR*[Title/Abstract])) OR (Clustered Regularly Interspaced Short Palindromic Repeats*[Title/Abstract])) OR (TALEN*[Title/Abstract])) OR (Transcription activator-like effector nuclease*[Title/Abstract])) OR (ZFN[Title/Abstract])) OR (Zinc-finger nuclease*[Title/Abstract])) OR (shRNA*[Title/Abstract])) OR (Short hairpin RNA*[Title/Abstract])) OR (Short hairpin ribonucleic acid*[Title/Abstract])) OR (Homing endonuclease*[Title/Abstract])) OR (Meganuclease*[Title/Abstract])) |

- 1. **EMBASE**

| #1 | 'chimeric antigen receptor t-cell':ti,ab,kw OR 'chimeric antigen receptor':ti,ab,kw OR 'CAR Transduced t':ti,ab,kw |
| --- | --- |
| #2 | 'knock out':ti,ab,kw OR knockout:ti,ab,kw OR 'knock down':ti,ab,kw OR knockdown:ti,ab,kw OR 'gene editing':ti,ab,kw OR 'genome edit':ti,ab,kw OR 'clustered regularly interspaced short palindromic repeat':ti,ab,kw OR talen:ti,ab,kw OR 'transcription activator like effector nuclease':ti,ab,kw OR 'zfn gene':ti,ab,kw OR 'zinc finger nuclease':ti,ab,kw OR 'short hairpin rna':ti,ab,kw OR 'homing endonuclease':ti,ab,kw OR meganuclease:ti,ab,kw |
| #3 | **#1 AND #2**  'chimeric antigen receptor t-cell':ti,ab,kw OR 'chimeric antigen receptor':ti,ab,kw OR 'CAR Transduced t':ti,ab,kw AND 'knock out':ti,ab,kw OR knockout:ti,ab,kw OR 'knock down':ti,ab,kw OR knockdown:ti,ab,kw OR 'gene editing':ti,ab,kw OR 'genome edit':ti,ab,kw OR 'clustered regularly interspaced short palindromic repeat':ti,ab,kw OR talen:ti,ab,kw OR 'transcription activator like effector nuclease':ti,ab,kw OR 'zfn gene':ti,ab,kw OR 'zinc finger nuclease':ti,ab,kw OR 'short hairpin rna':ti,ab,kw OR 'homing endonuclease':ti,ab,kw OR meganuclease:ti,ab,kw |

- 1. **Web of Science**

| #1 | TOPIC: ("CAR T*" OR "Chimeric antigen receptor*" OR "CAR Transduced T") |
| --- | --- |
| #2 | TOPIC: ("knock-out" OR "knockout" OR "knock-down" OR "knockdown" OR "Gene edit*" OR "Genome edit*" OR "CRISPR*" OR "Clustered Regularly Interspaced Short Palindromic Repeats*" OR "TALEN*" OR "Transcription activator-like effector nuclease*" OR "ZFN*" OR "Zinc-finger nuclease*" OR "shRNA*" OR "Short hairpin RNA*" OR "Short hairpin ribonucleic acid*" OR "Homing endonuclease*" OR "Meganuclease*") |
| #3 | **#1 AND #2**  TOPIC: ("CAR T*" OR "Chimeric antigen receptor*" OR "CAR Transduced T") AND TOPIC: ("knock-out" OR "knockout" OR "knock-down" OR "knockdown" OR "Gene edit*" OR "Genome edit*" OR "CRISPR*" OR "Clustered Regularly Interspaced Short Palindromic Repeats*" OR "TALEN*" OR "Transcription activator-like effector nuclease*" OR "ZFN*" OR "Zinc-finger nuclease*" OR "shRNA*" OR "Short hairpin RNA*" OR "Short hairpin ribonucleic acid*" OR "Homing endonuclease*" OR "Meganuclease*") Timespan: All years. Indexes: SCI-EXPANDED, SSCI, A&HCI, ESCI. |

- 1. **Cochrane**

| #1 | (CAR T):ti,ab,kw OR (Chimeric antigen receptor):ti,ab,kw OR (CAR Transduced T):ti,ab,kw |
| --- | --- |
| #2 | (Knock out):ti,ab,kw OR (Knock down):ti,ab,kw OR (Gene editing):ti,ab,kw OR (Genome editing):ti,ab,kw |
| #3 | **#1 AND #2** |

- 1. **Clinicaltrials.gov**

| #1 | *Intervention/treatment:* | CAR T OR "Chimeric antigen receptor*" OR "CAR Transduced T" |
| --- | --- | --- |
| #2 | ***Other terms:*** | "knock-out" OR "knockout" OR "knock-down" OR "knockdown" OR "Gene edit*" OR "Genome edit*" OR "CRISPR*" OR "TALEN*" OR "ZFN*" OR "ShRNA" OR "Homing endonuclease*" OR "Meganuclease*" |
| #3 | **#1 AND #2:**  *Intervention/treatment:* CAR T OR "Chimeric antigen receptor*" OR "CAR Transduced T"  *Other terms:* "knock-out" OR "knockout" OR "knock-down" OR "knockdown" OR "Gene edit*" OR "Genome edit*" OR "CRISPR*" OR "TALEN*" OR "ZFN*" OR "ShRNA" OR "Homing endonuclease*" OR "Meganuclease*" | |

**Table 1.** Summary of the findings in pre-clinical studies

| Reference & published year | Target cancer | Animal and cell line | | Route of CAR T cell administration | | | Target antigen | Disrupted gene(s) | | Purpose of gene editing | | Knockout/knockdown tool | Gene disruption efficacy | Concurrent treatments | | Main findings |
| --- | --- | --- | --- | --- | --- | --- | --- | --- | --- | --- | --- | --- | --- | --- | --- | --- |
| (Adachi, Terakura et al. 2024)  2024 | Lymphoma | NOD-Shi-SCID IL-2Rγ KO (NOG) mice)- Raji-ffluc cells | | Intravenous | | | CD19 | CUL5 | | Increase efficacy | | Genome-wide CRISPR-Cas9 | >95% | NA | | 1. CUL5 KO improves not only proliferation but also the effector roles of CAR T cell 2. CUL5 KO could improve the expansion potential of two types of CAR T cells in response to antigen stimulation 3. CUL5 KO elevates the proliferation of stimulated CD19 CAR T cells by delaying the degradation of target proteins or phosphoproteins |
| (Adeshakin, Zhou et al. 2023)  2023 Abstract | Osteosarcoma | Lung F331 OS metastasis mice model | | Intravenous | | | Reg-1 | B7-H3 | | Increase efficacy | | NA | NA | NA | | 1. Significant antitumor activity compared to CD8+ control CAR T cells 2. Significant survival advantage 3. Limited B7-H3-CAR T cell expansion and persistence, even though recurring tumors still expressed B7-H3 4. KO of Reg-1 in CD8+ B7-H3-CAR T cells improved their expansion post-infusion in spleen and lungs |
| (Agarwal 2020)  2020  Abstract | ALL | NALM6 | | | N/A | | CD19,  HER2 | CTLA-4 | | Increase efficacy | | CRISPR/Cas9 | N/A | No | | CTLA-4 KO CARs had significantly decreased tumor progression than controls. |
| (Agarwal, Aznar et al. 2023)  2023 | ALL | ALL-xenograft model. NOD-SCID-IL2rγ−/− (NSG) mice | | | Intravenous | | CD19 | PD-1 & CTLA-4 | | Increase efficacy | | CRISPR/Cas9 | NA | NA | | 1. CTLA-4-deficient CART19 cells have superior anti-tumor efficacy under stress-test conditions  2. Deletion of CTLA4 can bolster the fitness of human T cells  3. CTLA-4-deficiency permits unopposed CD28 signaling and prevents or delays CAR internalization |
| (Ajmal, Farooq et al. 2024)  2024 | Prostate cancer | 6- to 8-week old NSG mice -PC-3 cells, colorectal cancer model | | | Intravenous | | NKG2D | ADRB2 | | Increase efficacy | | shRNA | NA | NA | | 1. Mice treated with ADRB2-KD CAR-T cells showed significantly lower tumor activity and volume.  2. ADRB2 deficiency was associated with higher overall survival and no increased GVHD.  3. In a proof-of-concept experiment, injection of KD CAR-T cells in colorectal cancer model was also associated with smaller tumor volume and better survival outcomes.  4. Transcriptional analysis showed lower expression of exhaustion markers -PD-1 and TIM3- in mice receiving KD cells. |
| (Alizadeh, Nee et al. 2023)  2023 Abstract | GBM | Immunodeficient mice- GBM model | | | NA | | IL13Ra2 | TGFbR2 | | Increase efficacy | | CRISPR/Cas | NA | Pretreatment with a TGFbR1 inhibitor -LY3200882 | | Inducing TGFb resistance in vivo by knocking out TGFbR2 on CAR-T cells augmented the efficacy of CAR-T cells in a large immunosuppressive GBM tumor model in syngeneic mice. |
| (An, Pan et al. 2023)  2023 | AML | Female NCG (triple immunodeficient) mice- MV4−11 cells | | | Intravenous | | CD38 | PI3Kδ | | Decreasing CAR T cell toxicity (no observed effect) | | shRNA | NA | NA | | Treatment of human AML mouse models with both PI3Kδ-KD and WT CD38-directed CAR-T cells was associated with improved survival outcomes compared to control-treated mice -KD did not reduce antitumor activity. After 18 hours and 2 weeks of injection, neither the percentages of CD3+, CD4/CD8 ratio nor the levels of human cytokines -including, IL-2, IFNg, TNF, IL-6- differed significantly between the two group. Thus, KD of PI3Kδ did not improve CRS. |
| (Andreu-Saumell, Rodriguez-Garcia et al. 2024)  2024 | HER2+ malignancies | NGC mice-SKOV3, HCC1954,CAPAN2 cell lines | | | Intravenous | | LA HER2, HA HER2 | PD-1 | | Increase efficacy | | CRISPR/Cas9 | Both low-affinity and high-affinity PD-1 KO of 70%. | Durvalumab | | 1. PD-1/PD-L1 inhibition restores in vitro functionality of LA but does not impact HA CAR-T cells 2. PD-1 KO increases efficacy of LA but not HA HER2-28Z CART cells in vivo 3. PD-1 KO induces deeper changes in the transcriptome of LA as compared to HA HER2-28Z CAR-T cells 4. PD-1 KO increases the polyfunctionality of LA but not HA HER2-28Z CAR-T cells |
| (Angelos, Patel et al. 2023)  2023 Abstract | T-ALL and PTCL | T-ALL patient-derived xenograft mouse | | | NA | | CD2 | CD2 | | Limit fratricide | | NA | 81.2% ± 2.4 | NA | | 1. CART2 cells were highly effective in controlling tumor burden of PDX models.  2. Significant improvement was seen in overall survival compared to control mice (138 days vs. 30 days).  3. Long-term tumor control was proportional to CART2 cell persistence on day 28 and 100.  4. In one mouse model derived from a relapsed T-ALL, CART2 infusion was associated with tumor elimination and long-term CART2 cell persistence. |
| (Bachl, Talbot et al. 2023)  2023 Abstract | MM | NSG mice (NOD.Cg- Prkdc^scid^ Il2rg ^tm1Wjl^/SzJ)- OPM2 cell line | | | NA | | BCMA | CUL5 | | Increase efficacy | | CRISPR (using Cas9-RNP electroporation) | NA | No | | Mice treated with KO CAR-T cells had significantly lower tumor activity.  Significantly higher CAR-T cells were detected in BM but comparable in spleen, suggesting an antigen-specific advantage of KO models. |
| (Bailey, Vatsa et al. 2022)  2022 | CD19+ B-cell leukemia, lymphoma | 6-8-week-old NSG mice-  JeKo-1 or Nalm6 CBG-GFP+ cells | | | Intravenous | | CD19 | IFNγ | | Decreasing CAR T cell toxicity | | CRISPR-Cas9 | N/A | No | | 1. Reduced macrophage activation markers (CD69, CD86) and decreased pro-inflammatory cytokines (IL-6, MIP-1β, MCP-1, & IP-10)  2. IFNγ blockade did not reduce efficacy against CD19+ leukemias/lymphomas. |
| (Bailey, Takei et al. 2025)  2025 | Hematologic, breast, and pancreatic malignancies | NSG mice -JeKo1, Nalm6, AsPC-1, eO771 cell line | | | Intravenous | | CD28 OR Mesothelin OR EGFR | IFN-γ OR IFN-γR | | Increase persistence | | CRSIPR-Cas9 | NA | Cyclophosphamide in breast cancer mouse models. | | 1. In the mantle cell model (JeKo1), KO CAR-T cells showed similar antitumor activity plus increased CAR T cells. No significant differences in exhaustion profiles for KO and WT cells in vitro, but higher resistance to cell death in the former.  2. In pancreatic mouse models, mesothelin-directed CAR with IFN-γR KO had increased antitumor activity compared to both IFN-γ KO, and WT cells but increased CAR T cell expansion. Transcriptional analysis confirmed IFN-γR KO is associated with resistance to CD28 cell death.  IFN-γR KO had CR and rapid decline on tumor rechallenge.  Similar results also in breast cancer model. |
| (Balakrishnan, Leach et al. 2023)  2023  Abstract | DIPG xenografts | DMG mouse models | | | NA | | CD99 | CD99 | | Limit fratricide | | CRISPR-cas9 | NA | NA | | Systemic administration of KO cells enhanced in vivo persistence and complete clearance of tumor in mouse models, with long-term remission.  Regional administration into fourth ventricle showed increased survival and similar antitumor activities. |
| (Battram, Mane-Pujol et al. 2025)  2025 | MM | NOD-SCIDIL2gc−/− mice infused with ARP-1-GFP-ffLuc cells | | | NA | | BCMA | PRDM1 | | Increase efficacy | | CRISPR/Cas9 | >65% | NA | | 1. Blimp-1 KO CAR T cells effectively slowed and prevented disease progression 2. Tumor-exposed Blimp-1 KO CAR T cells confirmed the presence of a memory-like transcriptomic signature with repressed CAR T-cell dysfunction as mechanisms that could contribute to improved antitumor activity. |
| (Beavis, Henderson et al. 2017)  2017 | Breast carcinoma | 6-16-week-old C57BL/6 mice,  24JK-HER2,  E0771-HER2 | | | N/A | | HER2 | A2aR | | Increase efficacy | | shRNA | N/A | anti–PD-1,  A2AR antagonist | | 1. A2AR KO enhances antitumor function.  2. Treatment with A2AR KO CAR T cells and anti–PD-1 increased survival compared to treatment with WT CAR T cells and anti–PD-1 (40% vs 15%).  3. The efficacy of anti-HER2 CAR T cells can be increased by dual blockade of PD-1 and A2AR. |
| (Beckett, Chockley et al. 2023)  2023 | Osteosarcoma, lung adenocarcinoma | NSG mice - LM7, A549 cell line | | | Intravenous and intraperitoneal | | CD47 | CD47 | | Limit fratricide. | | CRISPR/Cas9 | NA | No | | 1. Unedited CAR Ts targeting CD47 downregulate CD47 surface expression to overcome fratricide. These cells are cytotoxic in vitro but lack antitumor activity in vivo. Importantly, they have limited persistence in vivo. 4. CD47 KO cells disappeared rapidly in mouse models. Thus, CD47 is required for CAR T-cell expansion and persistence in vivo. 5. Macrophage depletion enhances CD47-negative CAR T-cell persistence in vivo. |
| (Benton, Liu et al. 2025)  2025 Abstract | Solid tumors | NSG model - COR-L A11 cells | | | NA | | KRAS G12V (NeoCAR) | TCR | | Decreasing CAR T cell toxicity | | CRISPR/Cas9 | ~99% | No | | Mice injected with mKRAS NeoCAR showed significant improved survival and tumor reduction. Nonetheless, tumor relapse was seen in most cases.  Favorable safety profiles were seen over 90 days with edited CARs.  NeoCARs with inducible IL-12 were associated with higher antitumor activity but high toxicity. TCR KO in these cells showed limitation of IFNg response and lower weight loss, with retaining antitumor activity. |
| (Bolaños, Calviño et al. 2023)  2023 | AML | NSG mice- MOLM-13 cells | | | Intravenous | | CD33 | HLA-I & TCR | | 1. increase efficacy  2. Produce allogeneic CAR-T cells while avoiding GVHD | | CRISPR-based genome editing technologies with virus-free gene-transfer strategies using Sleeping Beauty transposon | More than 70% double negative cells | No | | Double KO CAR-T cells showed comparable antitumor activity to WT anti-CD33 CAR T cells, the structure did not suffer from instability resulting from CRISPR/Cas9 application, showing potential of this method for clinical applications. |
| (Braun, Pruene et al. 2023)  2023 | EBV+ BL | Nod.Rag.Gamma mice and Jiyoye/fLuc-GFP model- Daudi/fLuc-GFP, Jiyoye/fLuc-GFP cells | | | Intravenous | | CD19 OR EBV gp350 | TRAC | | Increase efficacy | | CRISPR/Cas9 | 20% of the total ~2 × 10(8) TCR-knocked-out (KO) generated cells | No | | 1. Both types of CAR-T cells showed cytotoxic effects against the BL lines in vitro.  2. mice challenged with BL and treated with CD19(KI)CAR-T cells showed delayed lymphoma dissemination with lower EBV DNA load 3. Administration of gp350(KI)CAR-T cells to mice after Jiyoye/GFP-fLuc challenge did not inhibit BL growth in vivo but reduced the EBV DNA load in the bone marrow and promoted gp350 antigen escape. |
| (Bridge, Johnson et al. 2024)  2024 | Solid tumors | NSG mice -Raji-Luc cells. | | | Intravenous | | CD19 | PD-1 & CISH & FAS | | Produce allogenic CAR-γδ T cells | | Multiplexed base editing | >90% at all three loci | NA | | Mice treated with triple base editing showed high antitumor activity and three times survival compared to unedited CAR-γδ T. Extensive expansion and penetration of CAR-γδ T cells was observed including in immune-privileged sites like brain.  The frequency of cell expansion was increased in gene-edited CAR T cells over control, but comparable to non-edited cells. |
| (Cai, Gouble et al. 2022)  2022 | Blastic plasmacytoid dendritic cell neoplasm | NSG mouse models-CAL-1 and Daudi cell lines | | | Intravenous | | CD123 | TRAC | | 1. Produce allogeneic CAR-T cells while avoiding GVHD  2. increase efficacy | | TALEN® | NA | No | | 1. UCART123 eradicate tumor and result in long-term disease free survival in a subset of primary patient-derived blastic plasmacytoid dendritic cell neoplasm mouse models.  2. Allogeneic UCART123 cells have potent anti-tumor activity in vivo. |
| (Calderon, Fleming et al. 2023)  2023 Abstract | B-ALL | Non-Human Primate Model -NALM6 cell | | | NA | | CD19 | PTPN2 | | 1. Increase efficacy, 2. limit fratricide | | CRISPR/Cas9 | 83.8 + 5.2% | Lymphodepletion (not specified) | | 1. increased expansion of *PTPN2* KO vs *PTPN2* WT CAR-T cells 2. PTPN2 KO CAR-T cells possess increased effector function, proliferation, and CNS infiltration versus PTPN2 WT CAR-Ts. 3. Enhanced efficacy was associated with increased severity of CRS and ICANS. |
| (Calviño, Ceballos et al. 2023)  2023 | AML | 8- to 12-week old NSG mice -MOLM-13-GFPLuc cells | | | Intravenous | | CD33 | HLA-I & TCR | | 1. Produce allogeneic CAR-T cells while avoiding GVHD  2. Increase efficacy | | CRISPR and Sleeping Beauty | NA | No | | Introducing a one-step production system of allogenic CAR T cells, with similar antitumor activity both in vivo and in vitro. High safety for site-specific gene editing was obtained. |
| (Cappabianca, Pham et al. 2024)  2024 | Neuroblastoma | Xenograft neuroblastoma models (NOD-SCID-γc-/- (NSGTM) mice)- CHLA-20 cell | | | Intraperitoneal | | GD2 | TRAC | | Increase efficacy | | GMP-compatibleCRISPR/Cas9 | >90% | NA | | 1. Compared to standard TRAC -CAR T cells, metabolic priming TRAC -CAR T cells showed less glycolysis, higher CCR7/CD62L expression, more bound NAD(P)H activity and reduced IFN-γ, IL-2, IP-10, IL-1β, IL-17, and TGFβ production at the end of manufacturing ex vivo , with increased central memory CAR T cells and better persistence observed in vivo  2. Metabolic priming with media during CAR T cell biomanufacturing can minimize glycolysis and enrich memory phenotypes and better responses in vivo. |
| (Carnevale, Shifrut et al. 2022)  2022 | Leukemia, Osteosarcoma | Xenograft NSG mice- LM7, Nalm6 cell line | | | Intravenous | | CD19 | RASA2 | | Increase efficacy | | CRISPR | NA | NA | | RASA KO was associated with improved tumor cell killing and survival outcomes and no increasing safety risk in leukemia xenograft model. Moreover, KO CAR-T cells were able to limit tumor in solid tumor model -osteosarcoma cell line- and improved survival. |
| (Certo, Kurtulus et al. 2023)  2023 Abstract | Non-Hodgkin Lymphoma | Xenograft NSG model | | | NA | | CD79a & CD20 | CBLB | | Increase efficacy | | CRISPR/Cas9 | NA | NA | | Improved CAR T cell expansion and antitumor activity was seen in CBLB KO compared to WT in xenograft model. |
| (Chan, Bolinger et al. 2022)  2022 Abstract | Not specified | ROR1+ PD-L1+ tumor bearing mice | | | NA | | ROR1 | PD-1 | | Introducing UCART cells with intrinsic checkpoint inhibitor activities | | TALEN | NA | NA | | Single infusion with PD-1 edited UCART was associated with rapid expansion, increase T cell proliferation with preferential memory phenotype, and better survival outcomes. |
| (Chang, Van Der Stegen et al. 2019)  2019  Abstract | ALL | NALM6 | | | N/A | | CD19 | TRAC | | 1. Produce allogeneic CAR-T cells while avoiding GVHD  2. Increase efficacy | | N/A | N/A | No | | FT819-iTs (TRAC KO) prolonged survival rate compared to primary CAR-T cells (survival >80 days, p> 0.1). |
| (Chang, Peralta et al. 2020)  2020 | ALL | N/A | | | N/A | | CD19 | TRAC | | Produce allogeneic CAR-T cells while avoiding GVHD | | N/A | N/A | No | | Allogeneic CARs were used. IL7 receptor fusion CARs inhibited tumor growth better than controls on day 34 (Log [BLI], 6.68 ± 1.93 vs. control group: 9.99 ± 0.23, p= 0.0143) |
| (Chen, López-Moyado et al. 2019)  2019 | Melanoma, thymoma,  colon adenocarcinoma | 8–12-week-old C57BL/6J,  B6.SJL-Ptprca  Pepcb  /BoyJ, Rag1−/− mice;  B16-OVA, EL4,  MC38  Cell lines | | | N/A | | CD19 | Nr4a1 (Nur77), Nr4a2 (Nurr1),  Nr4a3 (Nor1) | | Increase efficacy | | N/A | N/A | No | | Mice injected with  Nr4a TKO CAR T cells showed enhanced tumor regression, survival, and effector function. |
| (Chen, Yap et al. 2025)  2025 | Solid tumors | Syngeneic and xenogeneic mouse models-OVCAR-3 cell line | | | Intravenous | | LeY | RGS16, PD-1, NR4A2 | | Decreasing CAR T cell toxicity | | CRISPR/Cas9 | NA | No | | NR4A2-KO IL-12-KI CAR T cells mediated significantly enhanced antitumor responses against OVCAR-3 tumors, and had high resistance to rechallenge. This cell was associated with low GVHD unlike PD-1 KO CAR T which was associated with rapid weight loss.  In the IL-2 KI CAR T, RGS16 KO had higher antitumor activity compared to PD-1 and NR4A2. |
| (Chen, Deng et al. 2024)  2024 | Lymphoma | NCG mice model - Karpas 299 lymphoma cells | | | Intravenous and intraperitoneal | | CD5-CD30- CD19 | HLA-I & TCR | | Produce allogeneic CAR-T cells while avoiding GVHD | | CRISPR/Cas9 | NA | NA | | MU-CAR T had comparable antitumor efficacy as conventional CAR T cells in vivo. It also showed comparable tumor infiltration and cytotoxicity profiles to conventional CAR T cell. |
| (Chen, Tan et al. 2024)  2024 | B-cell malignancies | B-NDG (NOD.CB17-Prkdc^scid^Il2rg^tm1^/Bcgen) mice- Raji-Luc cells | | | Intravenous | | CD19 | β2M & TRAC | | Produce allogeneic CAR-T cells while avoiding GVHD | | CRISPR-Cas9 | >99% CD3 negative, >70% β2M negative | No | | 1. DKO UCART cells induced remission of leukemia cells with significant survival prolongation compared to CAR-T cells with positive β2M, TRAC, or both.  2.Evaluation of GVHD in NOD mice showed lower weight loss and IFN-g levels for DKO CAR-T cells compared to cells positive for β2M, TRAC, or both. (overall low alloreactivity with retained tumor-killing). |
| (Cheng, Zhang et al. 2025)  2025 | Leukemia | Xenograft mouse model -Nalm6-LUC cells | | | Intravenous | | CD19 | T316I mutation in LCK & TRAC & B2M | | 1. Produce allogeneic CAR-T cells while avoiding GVHD  2. Increase efficacy | | CRISPR/Cas9 & cytosine base editor | NA | Dasatinib | | LCK mutated UCAR-T cells showed increasing antitumor activity in vitro and in vivo, compared to conventional CD19 CAR T cells. No tumor growth was observed and most of the mice remained tumor free until day 28 in the first group. LCK mutated UCAR T cell was safe and was associated with stable body weight unlike control groups. |
| (Cherkassky, Morello et al. 2016)  2016 | Malignant pleural mesothelioma | 4-6-week old female NSG mice,  MSTO MSLN+ GFP-ffLuc+ | | | Intrapleural | | Mesothelin | PD-1 | | Increase efficacy | | shRNAs | >60% | No | | Transduced CAR T cells showed enhanced proliferation, cytotoxicity, and cytokine secretion but not greater tumor rejection. |
| (Choi, Yu et al. 2020)  2020  Abstract | GBM | Murine models of orthotopic, human GBM | | | Intravenous vs. intraventricular | | EGFRvIII | β2M, TRAC & PD-1 | | 1. Produce allogeneic CAR-T cells while avoiding GVHD  2. Increase efficacy | | CRISPR-Cas9 | >80% triple-knockout (TKO) | No | | Intraventricular, but not intravenous, TKO CAR T cells improved survival (p<0.001). |
| (Choi, Yu et al. 2019)  2019 | GBM | NSG mice,  Human glioma cell lines U87 and U251 | | | Intravenous (via tail vein) & intraventricular | | EGFRvIII | β2M, TRAC,  PD-1 | | 1. Produce allogeneic CAR-T cells while avoiding GVHD  2. Increase efficacy | | CRISPR/Cas9 | >80% DKO efficiency of β2M & TRAC | No | | 1. Intraventricular infusion of TKO CAR T significantly prolonged survival.  2. No signs of xenogeneic GVHD was observed. |
| (Chu, Nava-Barbero et al. 2024)  2024 Abstract | Tumor models | Murine tumor models | | | NA | | Not specified | TNFAIP3 (A20) | | Increase efficacy | | CRISPR/Cas9 | NA | NA | | A20 KO CAR T cells were associated with increasing antitumor efficacy compared to control. Metabolic analysis showed higher effector function and inclination of A20 CAR T cells, suggesting higher resistance to oxidative stress and possible persistence advantage. |
| (Chun, Kim et al. 2020)  2020  Abstract | T-cell leukemia,  B-ALL | NSG mice, Jurkat,  NALM6 | | | N/A | | CD5  CD19 | CD5 | | Increase efficacy | | CRISPR/Cas9 | 95-100% | No | | 1. CD5 KO CART5 showed significantly increased tumor control.  2. There was an increased expansion of CD5 KO CART5 in the peripheral blood compared to WT CART5. |
| (Condomines, Arnason et al. 2015)  2015 | ALL | 8-12-week old NSG mice,  GFP-FFLuc NALM6 | | | N/A | | CD19 | CTLA-4 | | Increase efficacy | | shRNA | 50% | No | | CTLA-4 down-regulation increases CD3 zeta-based CAR (19z1)-CD80+ T-cell efficiency but not that of CD3 zeta and CD28-based CAR (19-28z) T cells. |
| (Cooper, Staser et al. 2018)  2018  Abstract | CD19+ lymphoma | NOD-scid IL2Rgammanull (NSG) mice | | | N/A | | CD19 | TRAC | | Produce allogeneic CAR-T cells while avoiding GVHD | | CRISPR/Cas9 | N/A | NT-17 | | 1. NT-I7 increased the efficacy of TRAC KO CARs.  2. Mice treated with TRAC KO CART+NT-I7 showed no signs of GVHD.  3. CART+NT-I7 group survived longer than CART group which survived longer than NT-I7 only group. |
| (Cooper, Choi et al. 2018)  2018 | T-cell ALL | 6-10-week-old NSG mice,  CCRF-CEM | | | Intravenous | | CD7 | TRAC & CD7 | | 1. Produce allogeneic CAR-T cells while avoiding GVHD  2. Limit fratricide | | CRISPR/Cas9 | 72.8%±1.92 | No | | 1. WT T cells: Expansion of alloreactive T cells, severe GvHD (mean clinical GvHD score = 5.66), robust graft vs. leukemia effect  2. TRACΔ T cells: GvHD completely absent, T cells undetectable, considerable tumor burden  3. UCART7: no GvHD, effectively cleared T-ALL blasts |
| (Cox, Manriquez Roman et al. 2022)  2022 | B cell malignancies | NSG mice -JeKo1 cells | | | NA | | CD19 | GM-CSF | | Increase efficacy | | CRISPR/Cas9 | 60-70% | No | | GM-CSF KO CAR-T cell significantly increased T cell proliferation and antitumor efficacy.  KO cell was associated with lower late lymphoma relapse compared to WT CAR T -on day 30 to 50. No significant transcriptional difference related to memory phenotype was seen -suggesting higher antitumor activity related to higher persistence. Analysis also confirmed lower cell death related GM-CSF KO cells. |
| (Dai, Mu et al. 2022) 2022 | T-cell malignancies | 6-week-old female NOD-Prkdc^em26Cd52^Il2rg^em26Cd22^/Nju (NCG) mice- CCRF-CEM | | | Intravenous | | CD5/CD7 | CD5/CD7 | | 1. Increase efficacy  2. limit fratricide | | CRISPR/Cas9 | NA | | Cetuximab | CAR T cells were constructed either Tandem or Dual CD5/CD7 CAR T.  Both cells were associated with antitumor activity, improved survival and no weight loss. However Tandem CAR T were associated with higher T cell percentage on day 28. While both CAR T cells were associated with favorable outcomes, Tandem CAR T were associated with higher tumor suppression. |
| (Dai, Mu et al. 2021)  2021 | T-cell ALL | NSG mice, CCRF-CEM-firefly luciferase cells | | | Intravenous (via tail veins) | | CD5 | CD5 | | Limit fratricide | | CRISPR-Cas9 | >80% CD5 deletion | | No | CAR T cells (both standard and biepitopic) were resistant to fratricide, with biepitopic CD5KO FHVH3/VH1 showing better efficacy than either CD5 single-epitopic CARs. |
| (Das, Valton et al. 2023)  2023 | Breast cancer | Orthotopic mouse model of triple-negative breast cancer | | | Intravenous | | FAP | TRAC & B2M | | 1. Produce allogeneic CAR-T cells while avoiding GVHD  2. Increase efficacy | | TALEN | > 80% TCR and HLA-I double knock out | | Meso UCAR T cells and the checkpoint inhibitor anti-PD-1 | Targeting cancer-associated fibroblasts by FAP UCAR T-cells reduces desmoplasia and promotes T-cell infiltration and antitumor activity. Reduced resistance to Mesothelin-directed UCAR T and anti-PD-1 treatment and enhances antitumor activity. |
| (David, Schiele et al. 2025)  2025 | Metastatic colorectal cancer | Xenograft mice -Nalm6 cell line | | | Intravenous | | CD19 | PD-1 alone OR PD-1 & LAG3& TIM3& TIGIT & TGFBR2 | | 1. Increase efficacy  2. Overcome immune-suppressive TME by targeting checkpoint molecules -cells obtained from colorectal cancer liver metastases | | Multiplexed zinc finger repressors | NA | | NA | 1. PD-1-repressed CD19-CAR-T cells exhibited enhanced anti-tumor activity and improved survival 2. PD-1 repression alone did not increase cytotoxicity against a PD-L1-positive colorectal cell line in vitro. 3. ZFR-expressing lentiviral vectors targeting PD-1 and other immune checkpoint molecules significantly improved the cytotoxic activity in T cells of the immune-suppressive TME. |
| (Dawn Sakthi Vale, Wu et al. 2022)  2022 Abstract | liquid tumour | Immunodeficient xenograft mouse model | | | NA | | CD28 | LCK | | Produce allogeneic CAR-T cells while avoiding GVHD | | CRISPR/Cas9 | na | | na | LCK KO T cells showed reduced xenogeneic GVHD comparable to TCR knockout T cells in the immunodeficient mice.  In allogenic models, LCK KO CAR T showed lower GVHD and higher persistence and engraftment compared to TCR KO. |
| (De Munter, Buhl et al. 2024)  2024 | B-cell lymphoma | NSG mice- DLBCL patient-derived cell | | | NA | | CD70 (NanoCAR) | CD70 | | Limit fratricide | | CRISPR/Cas9 | NA | | No | CD70-specific nanoCAR T failed to show antitumor activity while CD70 KO show significant activities. Higher persistence of CD70 KO antiCD70 nanoCAR cells, neither shown by WT not CD70 KO anti-CD20 cell.  With lower extent of CD70-mediated exhaustion profile -possible proof for concomitant blockade of antigen targets in CAR T cells. |
| (Deeren, Maertens et al. 2020)  2020  Abstract | AML | Mice (not specified) | | | N/A | | NKG2D | MICA & MICB | | Increase efficacy | | ShRNA | N/A | | No | MICA & MICB KO led to 10-fold engraftment a week after injection and 2.6-fold increase in mouse survival |
| (Degagné, Donohoue et al. 2024)  2024 | Relapsed/refractory MM | Murine xenograft models of multiple myeloma | | | Intravenous | | BCMA | HLA-I complex & B2M & TRAC | | Produce allogeneic CAR-T cells while avoiding GVHD | | CRISPR/Cas9 | NA | | NA | 1. CB-011 cells were associated with improved antitumor activity and survival outcomes in vivo.  2. CB-011 cells did not show any GVHD in mouse models.  3. Additionally, the cells were protected from NK–mediated cytotoxicity in vitro and in vivo (immune cloaking). |
| (Dequeant, Sagert et al. 2021)  2021  Abstract | RCC, Leukemia | NOG mice,  A498,  Nalm6 | | | N/A | | CD70 | CD70,  β2M, TRAC | | 1. Increase efficacy  2. Produce allogeneic CAR-T cells while avoiding GVHD | | CRISPR/Cas9 | N/A | No | | 1. CD70 KO  improved CAR-T cell expansion.  2. TKO CARs continued to prevent tumor regrowth after in vivo re-challenge while DKO (CD70+) CARs failed to continue activity . |
| (Dharani, Cho et al. 2024)  2024 | Triple-negative breast cancer | 7–8 weeks old, female NSG mice- NCI-H226 tumor cells, either FAP+ or FAP- (on each flank) | | | Intravenous | | FAP | TRAC, PD-1 | | Produce allogeneic CAR-T cells while avoiding GVHD | | TALEN | NA | No | | FAP UCAR T cells showed significant accumulation of CAR T in the flank with FAP+ tumor. other FAP- flank showed higher CAR T cell infiltration compared to mice without any site of FAP positivity.  Significant tumor size reduction in the flanks with FAP+ tumor when administered with FAP CAR T, compared to WT and also compared to FAP- side (on-target effect). |
| (Diorio, Murray et al. 2022)  2022 | Childhood T-ALL | Patient-derived xenograft model, NSG - CCRF-GFP-Luc cells | | | NA | | CD7 | PD-1 & B2M & TRAC | | Produce allogeneic CAR-T cells while avoiding GVHD | | Cytosine base editors | 72.0% to 96.2% | No | | 7CAR8 cells significantly reduced tumor activity in a dose-dependent manner, compared to controls. Mice receiving 7CAR8 lived to study endpoint -day 35-, and the highest dose was associated with no disease recurrence.  7CAR8 cell inihibited in vivo leukemia activity in all PDX models. PD-1 KO increased survival outcomes related to PD-L1 expression. |
| (Doan, Mueller et al. 2024)  2024 | Leukemia | Xenograft mice -Nalm6cell | | | NA | | Not specified | FOXO1 | | Increase efficacy | | CRISPR/Cas9 | NA | No | | FOXO1 KO CAR T cells showed significantly lower antitumor activity and survival, indicating the crucial impact of FOXO1 -possible role of FOXO1 overexpression CAR T. |
| (Dörr, Gregor et al. 2024)  2024 Abstract | Solid cancer | Xenograft solid cancer mice model | | | NA | | PGE2 | EP2 & EP4 (PGE2 receptors) | | Increase efficacy | | CRISPR/Cas9 | NA | NA | | Double KO of PGE2 receptors rescued CAR T from PGE2-induced TME immune-suppression. As shown by improved efficacy and prolonged survival outcomes in vivo compared to WT CAR T cell administration. |
| (Dötsch, Svec et al. 2023)  2023 | ALL | Syngeneic immunocompetent OT-I Rag−/− mice model -Eμ-ALL cell line | | | NA | | CD19 | PD-1 | | Increase efficacy | | CRISPR/Cas9 | 90% | NA | | PD-1 KO CAR T cells were associated with long-term in vivo survival, 390 days. Notably, a higher expansion was seen for KO cells compared to WT cells, in the first 100 days.  PD-1 ablation did not result in differences in memory cells and persistence of CAR T cells. However, minimal concerns are present due to nonselective expansion of T cells. |
| (Engel, Steinfeld et al. 2025)  2025 | B-ALL | 6- to 8-week old male NSG mice -Nalm6 cell line | | | Intravenous | | CD19 | (TRAC or CD3E) & B2M & CIITA & PVR | | Produce allogeneic CAR-T cells while avoiding GVHD | | CRISPR/Cas9, adenine base editor |  |  | | Adenosine-base editor manufactured CAR-T cells showed comparable engraftment in leukemia mouse model, to conventional CAR T, whereas CRISPR/Cas9 edited expansion was impaired. This trend was also seen related to tumor clearance. Cas9-edited cells were associated with lower survival outcomes, overall suggesting disrupted fitness compared to base-editing technology. |
| (Evtimov, Nguyen et al. 2025)  2025 | Ovarian cancer | Non-obese diabetic NSG xenograft mouse model -TAG-72mid cancer cell line, OVCAR-3 | | | Intravenous | | TAG-72 | DGKα/ζ | | Increase efficacy | | CRISPR/Cas9 | 48.00% ± 16.82% DGKα. and 75.33% ± 2.08% DGK ζ | NA | | TAG-72 CAR/DGKa/z KO T cells ablate established tumors in vivo.  IL-4 levels were slightly higher following TAG72 CAR/DGKa/z KO T cell treatment compared to unedited T cells, but there was no significant difference between TAG-72 CAR-T cells and the other groups. No elevated levels of IL-10 or IL-13 were observed |
| (Fraessle, Tschulik et al. 2023)  2023 | B-cell lymphoma (Raji, CD19+) | Xenograft NSG-SGM3 mouse model; Human PBMC-derived primary T cells; EBV-transformed LCL (CD19+) and Raji lymphoma cells | | | Intravenous | | CD19 | PD-1 | | Increase efficacy | | CRISPR-Cas9 | NA | NA | | Engineered “feedback loop” CAR T cells express CAR only upon activation. They showed effective in vitro killing of CD19+ targets and in vivo clearance of lymphoma in mice. CAR expression declined after tumor clearance, suggesting potential safety advantage by allowing B cell recovery compared to conventional CAR T cells. |
| (Freitas, Belk et al. 2022)  2022 | Leukemia,  Osteosarcoma, melanoma | NSG mice-  NALM-6,  143B,  A375 | | | Intravenous, intramuscular, subcutaneous | | CD19, HER2, | MED12, CCNC | | Increase efficacy | | CRISPR-Cas9 | NA | NA | | Targeted MED12 deletion enhanced anti-tumor activity and sustained the effector phenotype in CAR- and TCR-engineered T cells |
| (Fu, Huang et al. 2024)  2024 | CLL, Burkitt lymphoma | (NOD-PrkdcscidIl2rγnull) mice; MEC1, HG3, Raji cell lines; Primary human T cells from healthy donors and CLL patients | | | Intravenous, Intraperitoneal, Subcutaneous | | CD19 | ITK | | Increase efficacy | | CRISPR-Cas9 | 86.6% for sgRNA1 and 96.9% for sgRNA2 | PF-06465469 (a potent ITK inhibitor that also inhibits BTK) and ibrutinib | | 1. TK-KO CD19-CAR-T cells showed enhanced long-term expansion and persistence in vivo  2. Significant reduction in T cell exhaustion markers  3. Better control of tumor relapse in Raji xenograft model with improved survival |
| (Gagnon, Litterman et al. 2022) 2022  Abstract | Solid tumors | NA | | | NA | | ALPG and mesothelin | FAS, PTPN2 | | Increase efficacy | | shRNA | NA | NA | | The shRNA knockdown of FAS and PTPN2 significantly enhances CAR T cell proliferation, persistence, and effector function, leading to better tumor control and increased T cell numbers in circulation |
| (Garner, Kelly et al. 2023) 2023  Abstract | AML | NPG mice | | | Intravenous (via tail vein) | | CLL-1 | PD-1,  TRAC,  Β2M | | Produce allogeneic CAR-T cells while avoiding GVHD | | CRISPR /Cas12a | NA | NA | | PD-1 knockout improves cytolytic potential and prolongs in vivo survival |
| (Georgiadis, Nickolay et al. 2024)  2024 | B-cell leukemia | NSG mice,  Umbilical cord blood T cells (CD62L+ selected), Humanized NSG mice; CD19+ Daudi tumor cells | | | Intravenous (via tail vein) | | CD19 | TRAC, CD52 | | 1. Produce allogeneic CAR-T cells while avoiding GVHD  2. Resistance to lymphodepleting agent alemtuzumab | | CRISPR-Cas9 | TRAC insertions / deletions (indels) 59-76% and CD52 indels 51-58% | Alemtuzumab | | CD62L-selected umbilical cord blood CAR19 T cells engineered with TRAC and CD52 knockout exhibited high transduction efficiency and minimal TCRαβ expression |
| (Georgiadis, Preece et al. 2018)  2018 | Burkitt's lymphoma | NSG mice,  CD19+  EGFP+  luciferase+ Daudi cells | | | N/A | | CD19 | TRAC | | Produce allogeneic CAR-T cells while avoiding GVHD | | CRISPR/Cas9 | >99% | No | | 1. CAR19+ TCR- cells showed more tumor clearance, higher CAR19 expression, lower tumor burden, and less exhaustion compared to CAR19+  TCR+ T cells.  2. There was no GVHD observed in mice injected with CAR19+ TCR- cells. |
| (Georgiadis, Rasaiyaah et al. 2020)  2020  Abstract | T-cell leukemia | NOD/SCID/γc mice.  Luciferase-labeled Jurkat T cells modified to express CD3, CD7, or both | | | N/A | | CD3 or CD7 | β2M, TRAC & CD52 (TKO) plus CD3 & CD7 | | 1. Produce allogeneic CAR-T cells while avoiding GVHD  2. Resistance to lymphodepleting agent alemtuzumab  3. Limit fratricide | | CRISPR-Cas9 | TKO efficiency of 92% | Alemtuzumab | | Mixture of anti-CD3 and anti-CD7 allogeneic CAR T cells decreased leukemic cell expansion and luciferase signals. |
| (Giuffrida, Sek et al. 2021)  2021 | Sarcoma,  breast cancer,  colon carcinoma | Female C57BL/6 human NSG mice,  E0771,  24JK,  MC38 | | | Intravenous | | HER2,  Lewis Y | A2aR | | Increase efficacy | | CRISPR/Cas9,  shRNA | CRISPR/Cas9  :87.3% ± 5.7%  shRNA: ~90% | IL-2 | | 1. A2AR KD using CRISPR/Cas9 significantly improved the efficacy of both murine and human CAR T cells and is superior to shRNA-mediated KD or pharmacological A2AR blockade.  2. While A2AR KD enhanced CAR T-cell effector function, it also reduced persistence. |
| (Goala, Davila et al. 2025) 2025  Abstract | B-ALL | IL2Rα knockout mice, B cell leukemia cell line, clinical patient serum | | | Injection | | CD19 | IFNγ, IL2Rα | | Decreasing CAR T cell toxicity | | NA | NA | NA | | IFNγ KO-CAR-T cells reversed CRS/neutropenia and restored cytokine IL-17, improving survival. |
| (Gomes-Silva, Atilla et al. 2019)  2019 | CD7+ AML | NSG mice-  KG-1a AML cells expressing FFluc | | | Intravenous | | CD7 | CD7 | | Limit fratricide | | CRISPR-Cas9 | Uniformly CD7-negative | No | | Mice injected with CARs on day 8 were all tumor-free on day 125, while those injected on day 5 had and controls had median overall survival of 97 & 54 days, respectively. |
| (Gouble, Philip et al. 2014)  2014  Abstract | Lymphoma | Orthotopic human CD19+ lymphoma xenograft immunodeficient mouse model | | | N/A | | CD19 | TRAC & CD52 | | 1. Produce allogeneic CAR-T cells while avoiding GVHD  2. Resistance to lymphodepleting agent alemtuzumab | | TALEN™ | N/A | Alemtuzumab | | 1. No decreased antitumor potency of DKO allogeneic CAR T cells.  2. No GVHD observed.  3. CAR T cells designed to coexpress RQR8 and could be depleted by rituximab. |
| (Gouble, Galetto et al. 2018)  2018  Abstract | MM | NSG mice,  MM.1S | | | Intravenous | | SLAMF7 (CSI) | TRAC SLAMF7 | | 1. Produce allogeneic CAR-T cells while avoiding GVHD  2. Limit fratricide | | TALEN® | N/A | No | | All mice in DKO group were alive on day 100 and had undetectable M protein levels on day 67, while vehicle controls had increased tumor size. |
| (Gough, Georgiadis et al. 2025)  2025 Abstract | Leukemia | NSG immunodeficient mice - luciferase-expressing Daudi B-cell lines | | | NA | | CD19 | TCR | | Produce allogenic CAR-T cells while avoiding GVHD, comparing virus-free and virus-mediated approaches | | CRISPR/Cas9 | NA | No | | 'knock-in' CAR-T cells exhibited less expansion and lower yields than those achieved for viral-mediated products, but similar in vitro cytotoxicity profiles.  Moreover, KI model showed better survival outcomes compared to conventional CAR T, still lower than lentiviral model, though. |
| (Graham, Jozwik et al. 2021)  2021 | High-grade lymphoma | NSG Raji luciferase | | | Intravenous (via tail veins) | | CD19 | TRAC | | Produce allogeneic CAR-T cells while avoiding GVHD | | TALEN® | N/A | No | | 1. CAR-T cells increased OS, but OS was higher in CARs from healthy donors (HD) than those derived from lymphoma patients (20 vs. 15 days).  2. HD-TRAC-KO CARs had non-inferior survival to HD CAR T cells, showing maintained efficacy after TRAC KO. |
| (Grauwet, Berger et al. 2024) 2024 | Hematological malignancies | Primary human T cells; mouse leukemia and lymphoma models | | | Intravenous | | CD19 | β2M & CIITA | | 1. Produce allogeneic CAR-T cells while avoiding GVHD  2. Increase  efficacy | | CRISPR-Cas9 and  shRNA | NA | NA | | CAR-T cell with reduced MHC I and II surface levels (termed "stealth" CAR-T cells), reducing allogeneic T cell without loss of anti-tumor activity in vivo. |
| (Gregor, Dörr et al. 2025) 2025  Abstract | Solid tumors | Murine and human CAR T cells; xenograft model; patient-derived tumor samples | | | Intravenous | | Not specified | EP2, EP4 | | Increase efficacy | | CRISPR-Cas9 | NA | NA | | Double knockout CAR T cells (EP2 -/-  EP4-/-) accumulated more in tumors and prolonged mouse survival. Single receptor knockout was insufficient. |
| (Guo, Jiang et al. 2018)  2018 | HCC | 6-8-week-old female NSG mice,  GPC3-positive PLC/PRF/5 and GPC3-  negative SK-HEP-1 | | | Intravenous | | GPC3 | PD-1 | | Increase efficacy | | CRISPR/Cas9 | 85% | No | | PD-1 KO enhanced efficacy, survival, cytokines  production, and infiltration of GPC3-CAR T Cells. |
| (Guruprasad, Carturan et al. 2024) 2024 | Lymphoma and solid malignancies | Mouse models of lymphoma and solid tumors; human CAR T cells; patient samples | | | Intravenous | | CD19 | BTLA | | Increase efficacy | | CRISPR-Cas9 | NA | NA | | BTLA knockout in CAR T cells enhanced tumor control, persistence, and effector function in lymphoma and solid tumor models. |
| (Guzman, Sugita et al. 2016)  2016,  Abstract | AML | PDX-AML mice | | | N/A | | CD123 (alpha-chain of IL3 receptor) | TRAC | | Produce allogeneic CAR-T cells while avoiding GVHD | | CRISPR-Cas9 | N/A | No | | 1 million UCART123 cells selectively eliminated AML cells resulting in normal hematopoiesis. Improved OS was observed and all mice are alive on day 167. |
| (Ho, Yu et al. 2023) 2023  Abstract | T-cell malignancies (T-ALL) | Jurkat xenograft model | | | NA | | CD5 | CD5 | | 1. Limit fratricide  2. Increase efficacy | | shRNA | NA | NA | | Downregulation of CD5 in CAR-T cells (CD5-KD C7CAR5) reduced fratricide more and maximized anti-tumor function. |
| (Hooper, Havens et al. 2018)  2018  Abstract | lung adenocarcinoma | A549 | | | N/A | | EGFR | CBLB | | Increase efficacy | | megaTAL | >90% | No | | Treatment with the CBLB megaTAL-treated CAR T cells led to complete and durable eradication of tumor which was more significant at lower CAR T cell doses. |
| (Hu, Chen et al. 2022)  2022 | CD19+ Lymphoma | NSG mice;  CA-46 | | | Intravenous | | CD19 | PD-1H | | Increase efficacy | | shRNA | N/A | No | | PD-1H KO enhances anti-tumor activity of CAR T cells and mice survival |
| (Hu, Zi et al. 2019)  2019 | Breast cancer | NSG mice,  BT549,  HeLa,  MCF7 | | | Intravenous (via tail vein) | | Mesothelin | PD-1 | | Increase efficacy | | Cas9 RNP | 59.2±9.0% | PD-1 blocking antibody (EH12) | | 1. PD-1 blocking antibody did not improve the effect of CAR T cells.  2. None of the treated groups showed autoimmune symptoms.  3. PD-1 editing led to better expansion of Meso CAR T cells. |
| (Hu, Zou et al. 2019)  2019 | Glioma | 6-8-week-old female NPG mice,  CD133-  overexpressing luc+ U251 | | | Intravenous | | CD133 | PD-1 | | Increase efficacy | | CRISPR/Cas9 | 91.5% | No | | 1. PD-1 KO enhanced survival.  2. No deaths or signs of GVHD or toxicity were found in mice injected with PD-1-deficient CAR T cells. |
| (Hu, Yi et al. 2020)  2020 | ALL | NSG mice- Nalm-6 leukemia cells | | | Intravenous | | CD19 | TRAC & GM-CSF | | 1. Produce allogeneic CAR-T cells while avoiding GVHD  2. Decreasing CAR T cell toxicity | | CRISPR-Cas9 | N/A | No | | 1. 5/6 (83%) of the mice were alive at 10 months (long-term survival).  2. Regarding safety concerns, CAR T cells decreased gradually after tumor eradication. |
| (Hu, Wang et al. 2022) 2022 | Pancreatic ductal adenocarcinoma | Pancreatic cancer cell lines, patient-derived tumors, Female Nod Scidg mice aged 6 weeks | | | Intravenous | | Mesothelin | CCR7 | | Increase efficacy | | CRISPR-Cas9 | NA | NA | | Inducible expression of chemokine CCL19 in mesothelin-targeting CAR T cells (mesoCAR-N19) enhanced recruitment of memory T cells via CCR7, increasing infiltration into pancreatic tumors. This led to superior tumor suppression and prolonged survival in xenograft models. |
| (Hu, Manner et al. 2023) 2023 | CD19+ hematological cancers | immunodeficient NSG mice, fully immunocompetent humanized mice | | | Intravenous | | CD19 | B2M & CIITA & TRAC | | Produce allogeneic CAR-T cells while avoiding GVHD | | CRISPR-Cas9 | NA | NA | | Hypoimmune (HIP) CAR T cells evade host immune rejection and GVHD, showing improved persistence and durable tumor control in fully immunocompetent humanized mice compared with conventional allogeneic CAR T cells. |
| (Hu, McAlister et al. 2025) 2025 | B-cell malignancies, post-transplant lymphoproliferative disorder (PTLD) | Human primary T cells and B cells; Nalm6 cell line; immunosuppressed humanized mouse models with tacrolimus and rapamycin treatment | | | Intravenous | | CD19 | FKBP12 & B2M & CIITA | | Increase efficacy | | CRISPR-Cas9 | More than 90% | NA | | FKBP12 knockout confers resistance to mTOR- and calcineurin inhibitor-based drugs, enabling CAR T cells to maintain cytotoxicity under high concentrations of tacrolimus and rapamycin. Only HIP-FKBP12 knockout CAR T cells persist and control Nalm6 tumors in immunosuppressed humanized mice. |
| (Jambon, Ruiz et al. 2024)  2024 | Relapsed/refractory B-cell malignancies | Stress xenograft models of WT (CD19+CD20+) RAJI (CLL, DLBCL) | | | NA | | CD19 &CD20 | β2M & TRBC1 & TRBC2 | | Increase efficacy | | Nonviral transposon-based system for transgene delivery | NA | No | | Superior anti-tumor antigen-specific activity compared to single-targeting products. |
| (Jiang, Chen et al. 2023) 2023 | T-ALL | Human primary T cells; T-cell leukemia cell lines; mouse xenografts (Jurkat and CCRF-CEM models) | | | Intravenous | | CD7 | CD7 | | 1. Limit fratricide  2. Increase efficacy | | CRISPR-Cas9 | ~80-90% knockout | NA | | CD7 gene knockout and insertion of CAR under EF1α promoter into CD7 locus reduced fratricide and enhanced expansion of CAR-T cells. CD7 CAR-T cells effectively killed T-ALL cells in vivo with better persistence. |
| (Jo, Das et al. 2022) 2022 | Hematological malignancies (AML, ALL, B cell) | Primary human T cells from healthy donors; leukemia cell lines RAJI, MOLM13; NSG mouse model; hIL-15 NOG mouse model | | | Intravenous | | CD22, CD123 | TRAC, B2M | | Produce allogeneic CAR-T cells while avoiding GVHD | | TALEN® | Up to ~96% | NA | | TALEN/AAV6-mediated editing efficiently disrupts TRAC and B2M loci with insertion of CAR and HLA-E, producing ΔTRACCARΔB2MHLAE T cells that evade host all responsive T cell and NK cell-mediated depletion. These cells retain antitumor activity in vivo, prolong tumor control in presence of cytotoxic NK cells. |
| (Jung, Bartoszek et al. 2023) 2023 | Hematologic malignancies, Solid tumors | Human CAR T cells; NALM-6 B-ALL, PC3 prostate cancer, Capan2 and AsPC1 pancreatic cancer xenografts; NSG mice | | | Intravenous | | CD19/  PSMA/mesothelin | EGR2 | | Increase efficacy | | CRISPR-Cas9 | NA | NA | | EGR2-deficient CAR T-cells demonstrate enhanced tumor control and survival in leukemia and solid tumor xenograft models. EGR2-associated gene signature correlates with poor patient response and survival, indicating EGR2 as a therapeutic target to improve CAR T-cell efficacy. |
| (Jung, Narayan et al. 2022) 2022 | Solid tumors (Prostate, Pancreatic), Hematologic (B-ALL) | Human CAR T cells (CD8+, CD4+), PC3-PSMA prostate cancer, NALM-6 B-ALL, Capan2 pancreatic cancer xenografts; NSG mice | | | Intravenous | | PSMA/ CD19 | PRDM1 & NR4A3 | | Increase efficacy | | CRISPR-Cas9 | NA | NA | | Dual PRDM1/NR4A3 KO sustains effector function, increases polyfunctionality, reduces exhaustion, and markedly improves CAR T-cell antitumor activity and survival, outperforming single KO, in solid tumor (prostate, pancreatic) and leukemia models. |
| (Jung, Kim et al. 2018)  2018 | GBM | 6-8-week-old female NSG mice,  U87vIII | | | Intravenous & Intratumoral | | EGFRvIII | DGKα/ζ | | Increase efficacy | | CRISPR/Cas9 | 80% to 90% | temozolomide | | 1. Treatment with dKO CAR-T cells led to  significant tumor regression on day 56 and an increase in tumor-infiltration.  2. IV and IT injection did not lead to different amounts of tumor growth and T cell infiltration.  3. DGK dKO showed a  synergistic effect compared to single KO. |
| (Kagoya, Guo et al. 2020)  2020 | ALL | 6-12-week-old male NSG mice,  NALM6-GL  K562 | | intravenous (via tail vein) | | | CD19 | Β2M, CIITA,  TRAC | | 1. Produce allogeneic CAR-T cells while avoiding GVHD  2. Increase efficacy | | CRISPR/Cas9 | 25% | No | | The triple KO T cells persisted better than HLA sufficient T cells without inducing GVHD. |
| (Kalaitzidis, Henderson et al. 2019)  2019  Abstract | MM, RCC, AML | NSG mice,  RPMI-8226, A498,  THP-1 | | N/A | | | CD19 (CTX110), BCMA (CTX120), CD70 (CTX130),  CD33 | β2M, TRAC | | Produce allogeneic CAR-T cells while avoiding GVHD | | CRISPR/Cas9 | >99.5% | No | | 1. CTX120, CTX130 and  anti-CD33 CAR-T cells all completely eradicated xenograft tumors of MM, RCC, AML respectively without any relapse.  2. The CTX110-treated mice  showed no signs of GvHD. |
| (Kath, Du et al. 2022) 2022  Abstract | Leukemia (mouse model), general immune cell therapy | Human T cells, NK cells, xenograft mouse models | | Intravenous | | | CD19 | CD247 & TRAC & β2M | | Produce allogeneic CAR-T cells while avoiding GVHD | | Virus-free homologous recombination | NA | NA | | Universal virus-free CAR reprogramming by truncated CAR integration at CD247 reduces transgene size, knock-in toxicity, and abolishes T cell allo-reactivity, allows all T cell and NK cell reprogramming. |
| (Kath, Du et al. 2022) 2022 | Leukemia (B-ALL) | Primary human T cells, Nalm6, MM1.S, xenograft mouse models (NRG mice) | | Intravenous | | | CD19, BCMA | TRAC | | Increase efficacy | | CRISPR-Cas9 | Up to >50% | Pharmacological inhibitors of DNA sensing and HDR enhancers | | Virus-free genome editing to insert CAR into TRAC locus improved T cell viability and CAR knockin efficiency while maintaining specificity and minimizing off-target effects. Resulting CAR T cells exhibited antigen-specific cytotoxicity and cytokine secretion, effectively controlling leukemia in murine xenografts. |
| (Keerthi, Balke-Want et al. 2024) 2024  Abstract | Small-cell lung cancer (SCLC) | Human SCLC cell lines, xenograft mouse models | | NA | | | B7-H3 (CD276) | TRAC | | Increase efficacy | | CRISPR-Cas9 | NA | NA | | Identified B7-H3 as overexpressed in SCLC and developed effective CKI CAR-Ts targeting B7-H3 with enhanced cytotoxicity via cJUN co-expression. |
| (Kelly, Sanchez-Pupo et al. 2023) 2023  Abstract | Leukemia, Ovarian cancer | Human T cells, NALM6 (CD19+) and SKOV3 (HER2+) cancer cell lines; mouse xenograft models | | Intravenous | | | CD19, HER2 | TRAC, AAVS1 | | CRISPR knock-in of CAR and reporter genes into TRAC and AAVS1 to enable therapeutic and multimodal imaging of CAR-T cells | | HiFi SpCas9 + AAV6 vectors | >75% | NA | | Demonstrated efficient multiplex gene editing with functional CAR and reporter integration enabling multi-modal in vivo tracking by MRI, PET, and BLI while maintaining cytotoxic function. |
| (Kienka, Korell et al. 2024) 2024  Abstract | Pancreatic adenocarcinoma | Primary human T cells, NSG mice bearing ASPC1 pancreatic adenocarcinoma xenografts | | Intravenous | | | Mesothelin | TRAC, multiple genes (Mario library) | | Increase efficacy | | CRISPR-Cas9 | NA | NA | | GATA3 knockout identified as enhancing early tumor clearance by CAR T cells in vivo. Gene knockout effects on CAR-T function dynamically changed over time, involving genes in TGF-beta, JAK-STAT, and trafficking pathways. |
| (Kim, Jeun et al. 2025) 2025 | Leukemia | Human primary T cells; Nalm6-Luc, Daudi-Luc cell lines; NSG mouse xenograft model | | Intravenous | | | CD19 | TET2 | | Increase efficacy | | shRNA | NA | NA | | TET2 knockdown CAR T cells showed significantly improved tumor control and survival in vivo. |
| (Kinder, Athena-Estrada et al. 2022) 2022  Abstract | MM | Human T cells; MM cell lines (RPMI8226, MM.1S); NSG mice xenograft model | | Intravenous | | | BCMA | TRAC & B2M & CIITA & CD47 | | Increase efficacy | | CRISPR/Cas12b | NA | NA | | Hypoimmune allogeneic CAR T cells with disrupted TCR, MHC I/II, and CD47 overexpression demonstrate potent in vivo anti-MM activity enhanced tumor control and persistence expected due to immune evasion. |
| (Koo, Jeong et al. 2025) 2025  Abstract | Hematologic cancer (CD19+ tumors) | Human T cells; in vivo tumor models | | NA | | | CD19 | DGKα& DGKζ | | Increase efficacy | | CRISPR gene editing (method not specified) | NA | NA | | DGKA/DGKZ knockout CAR T cells resist dysfunction under repeated antigen stimulation, maintain effector functions and tumor killing in vivo. |
| (Korst, O'Neill et al. 2024) 2024 | MM | MM cell lines (MM.1S, L363, UM9, U266), primary BM samples from MM patients,  NSG mouse xenograft model | | Intravenous | | | SLAMF7 (CS1) | TCRα (TRAC), SLAMF7 | | 1. Produce allogeneic CAR-T cells while avoiding GVHD  2. Limit fratricide | | TALEN® | NA | NA | | UCARTCS1, an off-the-shelf allogeneic SLAMF7 CAR T product, showed potent dose-dependent lysis of MM cell lines and primary MM cells from newly diagnosed and refractory patients. |
| (Lam, Barragan et al. 2022) 2022  Abstract | Solid tumors (lung cancer) | Human primary T cells; H1975 lung cancer xenograft model | | NA | | | ROR1 | NR4A1, NR4A2, NR4A3 | | Increase efficacy | | CRISPR-Cas9 | NA | NA | | NR4A3 KO combined with c-Jun overexpression synergistically reduces CAR T cell exhaustion, enhances cytokine production, persistence, and antitumor activity. |
| (Lam, Li et al. 2023) 2023  Abstract | Solid tumors (lung cancer) | Human primary T cells; H1975 lung cancer xenograft | | NA | | | ROR1 | NR4A3 | | Increase efficacy | | CRISPR-Cas9 | ~90% | NA | | LYL119 CAR T cells combining c-Jun overexpression, NR4A3 KO, Epi-R, and Stim-R technologies provide effective CAR T-cell  antitumor activity. |
| (Lau, Kwong et al. 2023)  2023 | B-NHL | Patient-derived DLBCL tumor xenograft (CTG-3020): NOD-SCID-gamma (NSG) mice, HEK293 cells transfected (NALM-6-ffLuc-eGFP/PD-L1), Toledo, MCL, tFL | | Intravenous | | | CD19 | TRAC & PD-1 | | 1. Increase efficacy  2. Produce allogeneic CAR-T cells while avoiding GVHD | | CRISPR/Cas9 | 98% for TRAC, 98% for PD1 | No | | CB-010 showed a dose-dependent increase in survival in several SQ and orthotopic -Toledo, MCL, tFL- models and a PDX DLBCL model and no  GVHD. No CB-010 cells were identified in any organs of the NOD mice in contrast to TCR+ cells injected.  CB-010 injection showed prolonged survival outcomes compared to PD1+ CAR-T cells in the PDX model, without an impact on the body weight. |
| (Lee, Lee et al. 2022)  2022 | Lymphoma,  ALL | 6-8-week-old NSG mice,  6-8-week-old female NOG mice,  Nalm-6, K562,  Raji-PD-L1 | | Intravenous | | | CD19 | PD-1,  TIGIT | | Increase efficacy | | shRNA | 19PTBBz-nt: 26.8% ± 11.44%  19BBz-nt:  32.3% ± 11.07% | No | | 1. PD-1/TIGIT downregulation reduced relapse and increased survival compared to PD-1 downregulation alone.  2. 19PTBBz-nt CARs suppressed tumor cells at lower doses compared to 19BBz-nt and led to longer mice survival. |
| (Lei, Li et al. 2024) 2024 | Glioblastoma, Breast Cancer | Human cell lines (HEK-293T, U251, MDA-MB-231), mouse tumor models (glioma, breast cancer) | | Intravenous | | | Various: EGFP, AKT1, endogenous proteins like BATF, KDM5B, EGFR; CAR-modified T cells | Targeted proteins including AKT, BATF, KDM5B, EGFR etc. | | Increase efficacy | | Endosome-microautophagy targeting chimera (eMIATAC) | NA | NA | | Using endosome-microautophagy targeting chimera (eMIATAC) knockdown of multiple gene targets was shown to be associated with superior cell killing compared to convensional CAR-T cells. |
| (Li, Su et al. 2024) 2024  Abstract | Hematologic malignancies (CD19+ tumors) | Human primary T cells; in vivo models | | NA | | | CD19 | TRAC, CD52, PD-1 | | Produce allogeneic CAR-T cells while avoiding GVHD | | Transformer base editor (tBE) | ~90% | NA | | Edited CAR-T cells showed comparable antitumor efficacy to unedited CAR-T cells, indicating tBE as a safer and effective alternative to CRISPR-Cas9 for universal CAR-T therapies. |
| (Li, Zhu et al. 2022) 2022 | Leukemia | Human primary T cells, Nalm6 tumor models, NSG mice | | Intravenous | | | CD19 | TRAC, B2M, CIITA, HLA-E | | Increase efficacy | | CRISPR-Cas9 | >99% loss of CD3 (TCR), >99% loss HLA-I, >98% loss HLA-II | NA | | Triple-edited CAR-T (ETUCAR-T) showed resistance to allo-rejection and maintained anti-leukemia activity with good safety profile. |
| (Li, Tang et al. 2020)  2020 | Pancreatic carcinoma | 6-week-old female NPG mice,  CRL5826,  pancreatic carcinoma PDX | | Intratumoral | | | Mesothelin | A2aR | | Increase efficacy | | CRISPR/Cas9 | N/A | No | | In the A2AR KO group smaller tumors and better tumor burden control was observed compared to the control group. |
| (Lin, Yamada-Hunter et al. 2025) 2025  Abstract | Solid tumors | Human primary T cells; mouse tumor models | | NA | | | GD2 (example) | MED12 | | Increase efficacy | | CRISPR-Cas9 | NA | NA | | Epigenetic silencing of MED12 enhances CAR T cytokine production, proliferation and tumor killing, similar to genomic KO. Concurrent IL2 locus activation further boosts functionality, potentially limiting toxicity by inducible cytokine release, offering a safer alternative to permanent gene KO. |
| (Liu, Zhang et al. 2017)  2017 | Lymphoma | Xenograft mouse model.  FFLuc-transduced Raji (Raji-ffluc) lymphoma cells intraperitoneally | | Intraperitoneal | | | CD19 | β2M & TRAC | | Produce allogeneic CAR-T cells while avoiding GVHD | | CRISPR-Cas9 | 100% β2M and 85% TRAC disruption | No | | Both standard and DKO allogeneic CAR T cells decreased tumor size compared to controls, showing preserved function after DKO. |
| (Liu, Zhang et al. 2021)  2021 | ovarian carcinoma | Xenograft mouse models, SKOV3 cell line | | | N/A | | Mesothelin | A2aR | | Increase efficacy | | shRNA | N/A | No | | A2aR-KD CAR T cells showed a significant increase in anti-tumor activity and reduction of the tumor burden compared to non-transduced CARs. |
| (Lonez, Bolsée et al. 2025)  2025 | MM | Immunodeficient NGS mice, Xenograft MM model with KMS-11 cell line | | | Intravenous | | BCMA | TCR | | 1. Produce allogenic CAR T and minimize GVHD  2. Increase efficacy. | | shRNA | 99.8% | NA | | 1. Mice injected with allogenic CAR-T cells (CYAD-211) were all alive during follow-up, with no evidence of GVHD, while control T cells were associated with it. CYAD-211 concentration was constant until day 14 and was almost undetectable by day 42.  2. MM model injected with control T cells were surrendered by the tumor on day 43, while CYAD-211 injected mice remained alive on the follow-up period (day 63), with a slowed progression of tumor by 3 weeks. |
| (Lu, C Lam et al. 2024) 2024  Abstract | lung cancer | Human primary T cells; H1975 lung cancer xenograft model | | | NA | | ROR1 | NR4A3 | | Increase efficacy | | Lentiviral transduction, gene editing, Epi-R & Stim-R (?) | NA | NA | | LYL119 displays potent antitumor activity with reduced T-cell exhaustion, maintained stem-like memory phenotype, epigenetic reprogramming supporting durable and functional CAR-T responses against solid tumors. |
| (Lu, Li et al. 2024) 2024 | Multiple Myeloma | Human MM cell lines (MM.1S, RPMI-8226, others), MM patient samples, NCG mice | | | Intravenous | | BCMA & CD47 | TRAC &  B2M | | Increase efficacy | | CRISPR-Cas9 | ~89-98% | NA | | UCAR-T cells targeting BCMA and CD47 exhibited strong antitumor activity, with combined targeting outperforming monospecific approaches. |
| (Lu, Yang et al. 2024)  2024 | MM | 6- to 8-week old female NCG mice - MM.1S-mCherry.ffLuc or RPMI-8226-mCherry.ffLuc cells | | | NA | | BCMA | TRAC & B2M | | 1. Produce allogeneic CAR-T cells while avoiding GVHD  2. Increase efficacy | | CRISPR-Cas9 | 63.51% for B2M and 55.88% for TRAC | No | | BC404-UCART (producing anti-CD47 antibody) cells significantly improved survival outcomes and inhibited tumor growth in xenograft mouse model. |
| (MacLeod, Antony et al. 2017)  2017 | CD19+ Burkitt’s lymphoma, transformed B lymphoblastoid,  CD19− CLL,  CD19− histiocytic lymphoma | 5-6-week-old female NSG mice,  Raji,  Daudi,  IM-9,  K562, U937,  NALM-6 | | | Intravenous (via tail vein) | | CD19 | TRAC | | Produce allogeneic CAR-T cells while avoiding GVHD | | AAV6 vector | >60% | No | | 1. On day 56, no mice in the mid- and high-dose CAR T cohorts showed tumor regrowth, 3/5 mice in the low-dose cohort were alive, with 1 mouse showing tumor regrowth.  2. All mice in the control group showed high tumor burden by days 17–19. |
| (Mai, Boyce et al. 2024) 2024 | Solid tumors (pancreatic cancer) | Human T cells engineered with mesothelin CAR; NSG mouse xenograft model | | | NA | | Mesothelin | ZFP36 | | Increase efficacy | | CRISPR-Cas9 | NA | NA | | ZFP36 KO slightly increases antigen-independent activation and cytokine secretion but does not improve CAR-T cell cytotoxicity, proliferation, or antitumor efficacy in vivo. |
| (Maldini, Messana et al. 2024) 2024 | B-cell malignancies | Human T cells; humanized immune system (HIS) mice, NSG mice with tumors- JeKo-1 mantle cell lymphoma | | | Intravenous | | CD19 | FKBP1A & TRAC & B2M & CIITA | | Produce allogeneic CAR-T cells while avoiding GVHD | | CRISPR-Cas9 | ~93.7% | Immunosuppressants: rapamycin, tacrolimus | | FKBP1A KO CAR-T cells resist immunosuppressant-mediated inhibition, enabling robust tumor control and persistence in vivo under immunosuppressive therapy. |
| (Maldonado-Pérez, Tristán-Manzano et al. 2022)  2022 | Lymphoma | NGS mice-Namalwa cells (expressing eGFP-NanoLuc | | | Intravenous | | CD19 | TRAC | | Produce allogeneic CAR-T cells while avoiding GVHD | | CRISPR/Cas9 | TCRKO>80% | No | | Mice treated with TCRKO cells showed 50% progression while 40% progression was observed in the control group at day 31.  Re-challenge at that day with Namalwa cells, showed similar survival for both groups up to day 56 (20% vs 17%). Notably, the two mice from WT group showed severe GVHD, while none was observed in TCRKO group.  High levels of CAR T in all tissues were identified with higher but non-significant levels in WT group. |
| (Mathur, Zhang et al. 2018)  2018  Abstract | MM | NSG mice,  MM.1S | | | Intravenous | | SLAMF7 (CSI) | TRAC SLAMF7 | | 1. Produce allogeneic CAR-T cells while avoiding GVHD  2. Limit fratricide | | TALEN® | N/A | No | | All DKO CAR T cells had undetectable M protein levels on day 50 (when euthanized), while control T cells had gradual increase in M protein levels. |
| (Mavuluri 2022) 2022  Abstract | B-ALL | B6 mouse model; human Pan-T CD19 CAR-T cells | | | Intravenous | | CD19 | PSGL-1 | | Increase efficacy | | CRISPR-Cas9 | NA | NA | | PSGL-1 KO CAR-T cells show improved tumor clearance, maintained central memory T cell phenotype, and reduced exhaustion profile. |
| (McEwan, Padalia et al. 2019)  2019,  Abstract | AML | THP-1 tumor xenograft model in NOG mice | | | Intravenous | | CD33 | β2M & TRAC | | Produce allogeneic CAR-T cells while avoiding GVHD | | CRISPR-Cas9 | N/A | No | | All the tumor cells were eradicated with gene-KO CARs without relapse in 30 days. |
| (Menegatti, Lopez-Cobo et al. 2024) 2024 | Leukemia | Mouse models (syngeneic and allogeneic mice); human CAR T cells, NSG mice bearing leukemia | | | Intravenous | | CD19 | FAS, B2M, CD3 | | Produce allogeneic CAR-T cells while avoiding GVHD | | CRISPR-Cas9 | FAS KO ~50-99%; B2M ~65% | NA | | FAS KO in CAR T cells outperforms B2M KO by enabling resistance to both T cell- and NK cell-mediated rejection, enhances anti-tumor control and persistence. |
| (Mise-Omata, Ando et al. 2023) 2023 | Solid tumors (MC38 colon adenocarcinoma, B16 melanoma) and hematologic (NALM6 leukemia) | Mouse models (Cd4Cre and Cd8Cre conditional SOCS3 knockout mice), human CAR-T cells in NSG mice bearing NALM6 | | | Intravenous | | CD19 | SOCS3 | | Increase efficacy | | CRISPR-Cas9 | NA | NA | | SOCS3 deletion switched IL-6 from a pro-tumor to anti-tumor cytokine via upregulation of type I IFN-regulated genes and enhanced metabolic activity in T cells and potent therapeutic efficacy of SOCS3-deficient human CAR-T cells |
| (Nakagawara, Ando et al. 2024) 2024 | Solid tumors (lung adenocarcinoma A549) | Human CAR-T cells from multiple donors; A549 tumor-bearing immunodeficient NSG mice | | | Intravenous | | HER2 | NR4A1 & NR4A2 & NR4A3 | | Increase efficacy | | CRISPR-Cas9 | NA | NA | | NR4A TKO CAR-T cells exhibit enhanced mitochondrial oxidative phosphorylation and fitness, stronger antitumor cytotoxicity, greater persistence within tumors, and improved survival in mice. |
| (Nakashima, Ouchida et al. 2025) 2025 | AML, ovarian cancer | Human CAR-T cells; AML cell lines and primary samples; NSG mice xenografted with AML and ovarian cancer tumors | | | Intravenous | | Membrane-bound TNF (TNF-NTF) | TNF | | Limit fratricide | | CRISPR/Cas9 | NA | NA | | TNF-NTF targeted CAR-T cells with TNF KO and G6/7R expression display superior proliferation, persistence, and potent antitumor activity against AML and solid tumors. |
| (Oumzil, Guerrero et al. 2022) 2022  Abstract | AML | Animal models of THP-1 tumor, xenogeneic GvHD models | | | NA | | CD83 | CD83, TRAC, B2M, ZC3H12A, TGFBR2 | | 1. Produce allogeneic CAR-T cells while avoiding GVHD  2. Limit fratricide | | CRISPR/Cas9 | NA | Belatacept | | CD83 KO anti-CD83 CAR-T cells show improved expansion, antitumor activity, and GvHD suppression; triple KO including Regnase-1 and TGFBRII genes gave robust cytotoxicity and durable tumor eradication, plus full prevention of GvHD. |
| (Ouyang, Jin et al. 2024)  2024 | MM | NGS mice- PD-L1/BCMA+K562 cells | | | Intravenous | | BCMA | PD-1 | | Increase efficacy | | shRNA | NA | No | | Compared to WT BCMA-CAR-T cells, PD1 KD cell infusion was associated with greater antitumor activity on imaging. These cells showed comparable cytotoxic ability with WT CAR-T cells together with anti-PD1 treatment. The antitumor activity correlated with OS.  No GVHD was observed related to PD1 KD CAR-T cells. |
| (Pal, Tran et al. 2024)  2024 | Clear cell RCC | NOG mice- A498 cells, NGS mice (GVHD explore) | | | Intravenous | | CD70 | TRAC & β2M & CD70 | | 1. Produce allogeneic CAR-T cells while avoiding GVHD  2. Increase efficacy | | CRISPR/Cas9 | >98% for TCR, >98% for CD70 | No | | Administration of CTX130 cells was associated with complete removal of the tumor in A498 RCC xenograft model, whereas CD70+ CAR-T cells was not associated with significant response. When comparing PD1 KO and WT, PD1 KO cells had reduced anti-tumor activity on rechallenge compared to PD1 WT CAR T cell infusion.  WT CAR had fatal GVHD within 21 days, whereas CTX130 injected mice survived until 12 weeks endpoint. |
| (Panowski, Srinivasan et al. 2022)  2022 | RCC | In vitro RCC cell lines (786-O, ACHN, REH), patient-derived xenografts, Cynomolgus monkeys | | | Intravenous | | CD70 | TRAC, CD52 | | 1. Produce allogeneic CAR-T cells while avoiding GVHD  2. Limit fratricide | | TALEN® | >95% | Rituximab | | CD70 CAR T cells show robust cytotoxicity against RCC models with two functionally distinct CAR classes varying in epitope binding and cytotoxicity. CAR expression masks CD70 on T cells preventing fratricide. CD70 KO not universally beneficial but improves some clones and Rituximab off-switch controls activity. |
| (Park, Monroy et al. 2022) 2022  Abstract | Solid tumors | Human tumor cell lines, xenograft mouse models | | | NA | | CD19 | TCR (alpha and beta) | | Produce allogeneic CAR-T cells while avoiding GVHD | | CRISPR-Cas9 | NA | NA | | Combination of Oncolytic virus (OV) expressing truncated CD19 and allogeneic CD19-CAR T cells from placental and PBMC sources shows potent tumor killing in vivo with reduced cytokine release. |
| (Patel, Ghilardi et al. 2022) 2022  Abstract | CD5+ Nodal T-cell lymphomas | NSG mice xenografted with Jurkat T-lymphoblastic leukemia; Human T cells | | | NA | | CD5 | CD5 | | 1. Increase efficacy  2. Limit fratricide | | CRISPR-Cas9 | >90% | NA | | Developed a GMP-grade autologous CD5 KO anti-CD5 CAR-T (Senza5TM) that was effective tumor clearance in preclinical models with no treatment-related lesions or abnormalities observed. |
| (Patel, Ghilardi et al. 2024) 2024 | T cell lymphomas, B cell leukemias/lymphomas; solid tumors | NSG mice xenografted with Jurkat, primary Sézary, T-ALL, Nalm6 (B-ALL), HLLM2 (Hodgkin lymphoma), AsPC1 (pancreatic cancer), PC3 (prostate cancer); murine lymphoma model A20 in BALB/c mice | | | Intravenous | | CD5, CD19, CD30, Mesothelin, HER2 | CD5 | | 1. Increase efficacy  2. Limit fratricide | | CRISPR-Cas9 | >90% CD5 | NA | | CD5 KO in CAR T cells prevents fratricide, enhances expansion, persistence, cytotoxicity, and antitumor efficacy across diverse hematologic and solid tumor models outperforming PD-1 KO |
| (Petersen, Bell et al. 2019)  2019  Abstract | High-grade glioma | U373 glioma-bearing mice | | | N/A | | IL-13Rα2 | PTPN6 | | Increase efficacy | | CRISPR-Cas9 | N/A | No | | Significant delay in tumor growth and increased survival in KO CAR T cells compared to standard CAR T cells |
| (Poirot, Philip et al. 2015)  2015 | lymphoma | 6-8-week- old NSG mice,  8-10-week-old NOG mice,  Raji cells,  Daudi cells | | | Intravenous (via tail vein) | | CD19 | TRAC, CD52 | | 1. Produce allogeneic CAR-T cells while avoiding GVHD  2. Resistance to lymphodepleting agent alemtuzumab | | TALEN® | 53.7% (±12.9%) | alemtuzumab | | 1. All mice in the dKO CAR19+ alemtuzumab group, 5/7 mice in the dKO CAR19 group and zero mice in the control group showed complete response by day 13 in the Raji experiment.  2. There was no significant difference in OS between mice treated with dKO-CART19 or CART19 cells, but they survived significantly longer than mice receiving non-modified T-cells in the Daudi experiment. |
| (Preece, Gough et al. 2025) 2025  Abstract | Relapsed/refractory ALL, AML, myeloma | Humanized NSG mice with Daudi B cell malignancy | | | Intravenous | | CD38 | CD38, TRBC1/2, B2M, RFX5 | | 1. Produce allogeneic CAR-T cells while avoiding GVHD  2. Limit fratricide | | Lentiviral delivery + cytidine base editing | 70%-100% | NA | | CD38 KO resolves CAR38 T-cell fratricide without reducing cytotoxicity and multiplex KO minimizes GVHD and immune rejection. universal CAR38 T cells showed superior leukemia control and extended survival compared with CAR19 in humanized mouse models. |
| (Prinzing, Zebley et al. 2021)  2021 | Osteosarcoma (intraperitoneal and intravenous lung-disseminated), & glioma | NSG mice-  LM7 osteosarcoma & U373 glioma models | | | Intravenous for osteosarcoma, intratumoral for glioma | | EphA2, HER2, or IL13Rα2 | DNMT3A | | Increase efficacy | | CRISPR-Cas9 | N/A | No | | 1. DNMT3A KO EphA2 and HER2 CARs had OS advantage over standard CARs in intraperitoneal (p<0.05) and lung-disseminated (p<0.01) osteosarcoma models, respectively.  2. DNMT3A KO IL13Rα2 showed slower glioma growth (p<0.05) and a non-significant 33-day increased median OS. |
| (Qiao, Chen et al. 2023) 2023 | Cholangiocarcinoma | Human tumor cell lines TFK-1, HuCCT1; cholangiocarcinoma patient-derived organoids; humanized NSG mice with tumor xenografts | | | Intravenous | | EGFR, B7H3 | PD-1, TIM3, TIGIT, TGFbR2, IL-10R, IL-6R | | Increase efficacy | | shRNA | 60-75% | None specified | | CAR-T cells targeting EGFR and B7H3 with combined shRNA clusters demonstrated superior tumor infiltration, cytokine production, memory phenotype, tumor control, and survival with no apparent toxicity. |
| (Rasaiyaah, Georgiadis et al. 2018)  2018 | T-ALL | 10-week-old female NSG mice,  Jurkat | | | Intravenous | | CD3 | TCRαβ, CD3 | | 1. Produce allogeneic CAR-T cells while avoiding GVHD  2. Limit fratricide | | TALEN® | 80% | No | | Transduced 3CAR T cells led to rapid clearance of TCR/CD3+ Jurkat cells. |
| (Ren, Liu et al. 2017)  2017 | ALL,  prostatic adenocarcinoma | 6-10-week-old NSG,  NALM6,  PC3-CBG | | | Intravenous (via tail vein) | | CD19,  PSCA | β2M, TRAC,  PD-1 | | 1. Produce allogeneic CAR-T cells while avoiding GVHD  2. Increase efficacy | | CRISPR/Cas9 | 65.4% (TCR and β2M double-negative) | No | | 1. Ablation of TCR alone or with HLA-I did not affect anti-tumor activity while PD-1 KO enhanced the efficacy of T cells.  2. GVHD was observed in 4/5 mice treated with standard CARs but none of the mice treated with TCR- or TCR/HLA-I double negative CARs. |
| (Ren, Liao et al. 2024) 2024 | T cell malignancies (CTCL, PTCL, T-ALL) | Humanized NSG mice with patient-derived xenografts (Vβ2+ malignant T cells); Jurkat-TRBV20-1 cells; healthy donors and patient T cells | | | Intravenous | | TCR-Vβ2 (TRBV20-1) | TRAC, B2M, CIITA | | 1. Produce allogeneic CAR-T cells while avoiding GVHD  2. Increase efficacy | | CRISPR-Cas9, AAV6 vector | >70% | NA | | Successfully generated allogeneic CAR-T cells specific for TCR-Vβ2 with minimal off-target killing and GVHD that was effective and specific for killing of malignant Vβ2+ T cells in patient-derived xenografts with persistence and specific anti-tumor activity in long-term mouse models. |
| (Richter, Cranert et al. 2019)  2019  Abstract | Multiple myeloma | NSG xenograft model implanted with RPMI-8226 | | | - | | BCMA | TCR | | Produce allogeneic CAR-T cells while avoiding GVHD | | high-fidelity CasCLOVER™ (CC) Site-Specific Gene Editing System | >99.5% TCR knockout | No | | Mice treated by allogeneic TCR-KO CAR T cells showed tumor reduction beyond detection in two weeks and all stayed alive, while controls died within 5-7 weeks. |
| (Roders, Nakid-Cordero et al. 2024) 2024 | Multiple myeloma | Humanized mouse models - CD34+ cells | | | Intravenous | | CD38 & SLAMF7 (CS1) | CD38 | | 1. Limit fratricide  2. Increase efficacy | | CRISPR-Cas9 | Up to 95% | No | | Developed DCAR T cells with CD38 deletion showing enhanced anti-myeloma activity in vivo; DCAR T cells exhibited specific targeting with reduced toxicity compared to single anti-CD38 CAR-T therapy. |
| (Rupp, Schumann et al. 2017)  2017 | Myelogenous Leukemia | 6-10-week-old female NSG mice,  K562 | | | Intravenous | | CD19 | PD-1 | | Increase efficacy | | Cas9 RNP | >50% | No | | PD-1 KO increased median survival compared to controls (35 days vs. 21 days). |
| (Salim, Shaikh et al. 2023) 2023  Abstract | Glioblastoma | Human GBM xenograft in NSG mice | | | Intravenous | | CD133 | TRAC | | Produce allogeneic CAR-T cells while avoiding GVHD | | CRISPR-Cas9 | NA | NA | | Allogeneic TCR-KO CAR-T may overcome limitations of autologous manufacturing and improve therapeutic outcomes in GBM. |
| (Sanber, Nawas et al. 2020)  2020  Abstract | GBM | orthotopic LN229-GBM mouse model | | | N/A | | HER2 | PTPN11 | | Increase efficacy | | CRISPR-Cas9 | N/A | No | | Better tumor control and overall survival |
| (Schiffer-Mannioui, Leduc et al. 2021)  2021  Abstract | Pancreatic tumor,  pleural mesothelioma tumor | N/A | | | Intravenous,  Intrapleural | | Mesothelin | TRAC, CD52,  TGFbR2 | | 1. Produce allogeneic CAR-T cells while avoiding GVHD  2. Resistance to lymphodepleting agent alemtuzumab  3. Increase efficacy | | TALEN® | N/A | alemtuzumab | | TKO anti- MSLN CAR T cells showed potent antitumor activity. |
| (Senz, Metzger et al. 2020)  2020  Abstract | Pancreatic cancer | CD4-Cre Ido1 fl/fl mice | | | N/A | | EpCAM | IDO1 | | Increase efficacy | | N/A | N/A | No | | IDO1 knockout CAR T cells showed improved  survival and tumor control and comparable proliferation and  cytotoxic function to WT CAR T  cells. |
| (Shang, Huang et al. 2025) 2025 | AML | Human AML cell lines (HL-60, MOLM-13, OCI-AML2), primary AML patient samples, multiple xenografts and PDX mouse models | | | Intravenous | | CD97 | ADGRE5 | | 1. Limit fratricide  2. Increase efficacy | | CRISPR-Cas9 | ~67% | NA | | CD97 KO in CAR-T cells prevent fratricide, enhance proliferation and eradicate AML blasts and leukemia stem/progenitor cells in diverse xenograft models with minimal toxicity to HSPCs. |
| (Shi, Zhang et al. 2025) 2025 | Solid tumors, B cell tumors | Murine C57BL/6N (WT and Trim21 KO), LLC, MC38, MC38-hCD19, LLC-hCD19; Human Jurkat and MOLT-4 | | | Intravenous | | CD19 | TRIM21 | | Increase efficacy | | CRISPR/Cas9 | NA | Anti-CTLA-4 and anti-PD-1 antibodies | | TRIM21 KO reduces PD-1 protein via impaired K63-linked ubiquitination of PD-1, enhancing CD8+ T and CAR-T cell activation and anti-tumor activity. Trim21 KO potentiates anti-CTLA-4 therapy and enhances anti-CD19 CAR-T efficacy against tumors in vivo. |
| (Shirinbak, Grant et al. 2022) 2022  Abstract | Multiple liquid and solid tumors (e.g. leukemia, breast, cervical, ovarian cancers) | Human cell lines: Nalm6, MDA-MB231, CasKi; orthotopic ovarian tumor mouse model | | | NA | | MICA/B CAR target; Fc receptor CD16a (hnCD16) | TRAC, CD38 | | 1. Produce allogeneic CAR-T cells while avoiding GVHD  2. Increase efficacy | | CRISPR-Cas9 | >95% | Anti-PDL1, daratumumab | | hnCD16 armed CAR iT cells showed enhanced activation, cytokine production, tumor cytotoxicity, and sustained tumor control compared to CAR alone. In vivo ovarian tumor model showed improved tumor control and prevention of relapse with combination therapy. |
| (Sodji, Shea et al. 2025) 2025 | Neuroblastoma | Human neuroblastoma xenograft models (CHLA-20, SY5Y), melanoma cell line (M21) in NRG mice | | | Intravenous | | GD2 | TRAC | | Increase efficacy | | CRISPR-Cas9 | NA | Low-dose radiopharmaceutical therapy (177Lu-NM600) | | Low-dose 177Lu RPT prior to GD2 TRAC-CAR T cells enhances tumor regression and survival in neuroblastoma models. Irradiation increases CAR T cell viability and infiltration in TME. |
| (Soltantoyeh, Akbari et al. 2024) 2024 | Cervical cancer | Mice xenografted with HeLa cells | | | Intratumoral | | Mesothelin | TIM3, A2aR | | Increase efficacy | | shRNA | NA | NA | | Dual KD of TIM-3 and A2aR in MSLN-CAR T cells significantly reduced tumor volume and improved survival compared to single KD or control, whereas caution was needed due to observed toxicity for single KD groups. |
| (Sommer, Boldajipour et al. 2019)  2019 | MM | 8-12 weeks NSG mice- Molp-8 or MM.1S cells | | | Intravenous | | BCMA | TRAC & CD52 | | 1. Produce allogeneic CAR T cells and avoid GVHD  2. Resistance to lymphodepleting agent alemtuzumab | | TALEN® | N/A | Alemtuzumab | | 1. DKO CARs had similar anti-tumor ability to normal CAR T cells, with potent anti-tumor activity in large scale production of allogeneic CAR T cells. |
| (Stenger, Stief et al. 2020)  2020 | ALL | | NSG mice, NALM6,  ALL-265 PDX | | | Intravenous | CD19 | | TRBC | Produce allogeneic CAR-T cells while avoiding GVHD | | CRISPR/Cas9 | 78.2% | No | | 1. treatment with TCR+ CARs led to an improved prevention of leukemia re-growth and prolonged survival rates compared to TRBC- CAR-T cells.  2. Treatment with TCR+ CARs led to sacrifice of 4 mice due to clinical signs of GvHD. |
| (Stepanova, Volkov et al. 2024) 2024 | Acute leukemia and hematologic malignancies | | Human leukemia and lymphoma cell lines (Nalm-6, Jeko-1, THP-1, Jurkat), primary human hematopoietic cells, humanized PBMC mice, mouse xenograft models | | | Intravenous | PTPRC | | PTPRC | Limit fratricide | | CRISPR-Cas9 | >85% | Dasatinib kinase inhibitor | | CD45 knockout CAR T cells prevent fratricide, maintain T cell function, display potent cytotoxicity, improve survival and reduce GVHD-like symptoms in humanized mouse model. |
| (Sterner, Sakemura et al. 2019)  2019 | ALL | | 8-12-week old female NSG mice,  NALM6 | | | N/A | CD19 | | GM-CSF | 1. Decreasing CAR T cell toxicity  2. Increase efficacy | | CRISPR/Cas9 | 60% | Lenzilumab,  anti-mouse GM-CSF neutralizing antibody | | 1. GM-CSF antibody therapy with CAR-T therapy ameliorates CRS and improves tumor burden control compared with isotype-control treatment with CAR-T therapy.  2. GM-CSF KO CART19 cells improved OS and tumor burden control significantly. |
| (Stewart, Siegler et al. 2024) 2024 | Hematological malignancies | | JeKo-1 cell- murine xenograft models | | | Intravenous | CD19, BCMA, CS1 | | IL4, IL4R | Increase efficacy | | CRISPR-Cas9, shRNA | NA | IL-4 neutralizing antibodies | | IL-4 induces reduced CAR T proliferation, cytokine production, increased inhibitory receptor expression. IL-4 or IL4R knockout and IL-4 neutralizing antibodies restore CART function and improve antitumor efficacy in vivo. |
| (Su, Yao et al. 2024) 2024 | B-cell lymphoma | | Human B-cell lymphoma lines (Raji, Daudi); NSG mouse xenograft with Raji tumors | | | Intravenous | CD19 | | ACAT1 | Increase efficacy | | shRNA | NA | NA | | ACAT1 knockdown in CD19-CAR T cells increases activation marker, cytokine secretion, proliferation, and degranulation, leading to enhanced cytotoxicity and improved tumor control and survival in vivo. |
| (Sugita, Galetto et al. 2022) 2022 | AML | | Human AML primary samples, AML cell lines, patient-derived xenografts (PDX), xenograft mouse models, humanized bone marrow (BM) | | | Intravenous | CD123 | | TRAC | Produce allogeneic CAR-T cells while avoiding GVHD | | TALEN® | NA | Rituximab | | UCART123 allogeneic CAR T cells selectively target AML leukemic stem cells, preferentially kill AML over normal hematopoietic cells in vivo and show potent antileukemia effect with improved survival. Safety with low GvHD risk and efficacy can be controlled with rituximab. |
| (Tang, Liu et al. 2024) 2024 | MM | | Human MM patient samples; MM cell lines (RPMI-8226, MM.1S) engineered for CD200 expression; MM tumor xenografts in NSG mice | | | Intravenous | BCMA, TnMUC1 | | CD200R | Increase efficacy | | CRISPR-Cas9 | NA | NA | | Engineering CAR-T cells with CD200R-CD28 switch receptor converts inhibitory CD200 signal to activating co-stimulation, enhancing CAR T poly-functionality, cytokine secretion, metabolism, proliferation, and in vivo tumor control. |
| (Tang, Cheng et al. 2020)  2020 | Lung cancer,  pancreatic carcinoma | | 6-week-old female NPG mice,  CRL5826,  pancreatic carcinoma PDX | | | Intravenous,  intratumor | mesothelin | | TGFBR2,  PD-1 | Increase efficacy | | CRISPR/Cas9 | 50%–80% | No | | 1. TGFBR2 KO improved tumor elimination efficacy.  2. GvHD occurred in all mice treated with TGFBR2 KO CAR T cells after i.t. injections but in no mice after i.v. injection.  3. TGFBR2 and PD1 dKO is necessary for efficient elimination of tumors with PDL1 overexpression. |
| (Tipanee, Samara-Kuko et al. 2022) 2022 | Hematological malignancies | Human T cells, NALM6 leukemia mouse xenografts | | | Intravenous | | CD19 | TRAC & TRBC2 | | Produce allogeneic CAR-T cells while avoiding GVHD | | CRISPR-Cas9 | ~80% | NA | | Non-viral platform combining CRISPR-Cas9 for TCR KO and Sleeping Beauty transposon for CD19 CAR expression generates functional TCR-negative CAR T cells with potent antitumor activity in vivo, with reduced alloreactivity and GVHD development. |
| (Trefny, Kirchhammer et al. 2023) 2023 | Solid tumors (melanoma, colon carcinoma) and hematologic cancers | Human T cells (ex vivo exhaustion model), NY-ESO-1 TCR-transduced T cells, murine OTI T cells, human CAR-T cells, various cell lines and tumor xenografts | | | Intravenous | | CD19 (for CAR-T), NY-ESO-1 TCR | SNX9 | | Increase efficacy | | CRISPR-Cas9 | NA | NA | | SNX9 deletion reduces TCR/CD28-mediated signaling and NFAT activation, lowers exhaustion, enhances cytokine secretion, and improves CAR-T and T cell antitumor efficacy in vivo, with potential to reduce toxicity. |
| (Vale and Gascoigne 2025) 2025  Abstract | Multiple cancers | Human and mouse T cells; xenograft tumor models | | | Intravenous | | Various | LCK, TCR | | Produce allogeneic CAR-T cells while avoiding GVHD | | CRISPR-Cas9 | NA | NA | | LCK knockout suppresses TCR activation reducing alloreactivity and GVHD, improves engraftment/persistence relative to TCR knockout, enhances cytotoxicity, reduces exhaustion, and supports better tumor control in vivo compared to TCR KO CAR-T. |
| (Wang, Wu et al. 2025) 2025 | Solid tumors | 6- to 8-week old NSG mice and BALB/c mice - HCT116-mEpCAM cells | | | Intravenous | | Various (solid tumor CAR targets) | ITGAL & ITGA4 & PSGL-1 | | Increase efficacy | | CRISPR-Cas9, shRNA | NA | Cyclophosphamide | | Triple KO/KD CAR-T cells showed significantly reduced on-target, off-tumor toxicity in vivo, while maintained tumor-killing efficacy in mouse models. |
| (Wang, Li et al. 2021)  2021 | Pancreatic cancer | Female NPG mice.  CRL5826-PD-L1-luci tumor cells | | | intratumorally or intravenously | | Mesothelin | TRAC, PD-1 | | 1. Produce allogeneic CAR-T cells while avoiding GVHD  2. Increase efficacy | | CRISPR-Cas9 | PD-1 & TRAC KO of 90% | No | | 1. DKO CAR T cells eliminated the tumor faster and more efficient than standard CAR T cell.  2. TCR KO caused disadvantages in engraftment and/or proliferation |
| (Wang, Prager et al. 2021)  2021 | GBM | 6-8-week-old NSG mice,  IDH1/2-wildtype | | | Intracranial | | IL13Rα2,  HER2 | TLE4, IKZF2, TMEM184B,  EIF5A | | Increase efficacy | | CRISPR/Cas9 | N/A | No | | These gene knock outs increased antitumor activity and OS of tumor-bearing mice. |
| (Ward, Senaoui et al. 2024) 2024  Abstract | medulloblastoma | Human CAR-T cells; orthotopic xenograft models with MB cell lines (D341, HDMB03, D425) | | | NA | | B7-H3 | DNMT3A, TET2 | | Increase efficacy | | CRISPR/Cas9 | NA | NA | | DNMT3A knockout significantly improves CAR-T cell persistence and expansion, and enhances tumor control in vivo, outperforming TET2 knockout, while DNMT3A KO alone was insufficient to cure aggressive tumors |
| (Ward, Adeshakin et al. 2024) 2024  Abstract | Medulloblastoma, DIPG | Human CAR-T; PDX and syngeneic murine models (NSG, C57BL/6) | | | NA | | B7-H3 | Regnase-1 (Reg-1) | | Increase efficacy | | CRISPR/Cas9 | NA | NA | | Reg-1 knockout enhances CAR-T anti-tumor activity and survival but causes severe neurotoxicity linked to meningoencephalitis and immune infiltration and toxicity limits long-term survival despite tumor regression. |
| (Wei, Luo et al. 2019)  2019 | Lymphoma,  Lung adenocarcinoma | 4-6-week-old female NPG mice,  Raji-luc lymphoma,  A549–19luc adenocarcinomic human alveolar basal epithelial cells | | | Intravenous (via tail vein) | | CD19 | PD-1 | | Increase efficacy | | shRNA | 72% (S3-CART19) and 88% (S4-CART19) | No | | PD-1 knockdown impaired the persistence, proliferation, antitumor activity of CAR-T cells |
| (Wellhausen, O'Connell et al. 2023) 2023 | Hematologic malignancies | Human primary T cells and HSCs, AML cell lines, multiple in vivo mouse xenograft and engraftment models | | | Intravenous | | CD45 | PTPRC | | 1. Limit fratricide  2. Increase efficacy | | CRISPR base editing (ABE) | >85-90% | NA | | Epitope base editing of CD45 prevents CAR-T fratricide and off-target killing of normal hematopoietic cells.; edited HSCs engraft and differentiate normally. CAR-T with edited CD45 retain anti-tumor function in vivo. |
| (Wen, Huo et al. 2021)  2021 | B-cell Burkitt’s lymphoma | | NSG (Prkdc^scid^IL2rg^tm1^/Bcgen) mice- Raji-Luc cells | | Intravenous | | CD19 | | IL-6 | | Decreasing CAR T cell toxicity | shRNA | N/A | No | | IL-6-deleted CAR T cells prolonged survival, killed lymphoma cells, decreased kidney, liver, and spleen involvement, alleviated clinical symptoms, and produced cytokines compared to controls with no significant CNS toxicity, immunotoxicity, or tumorigenicity. |
| (Wiebking, Lee et al. 2021)  2021 | ALL | | 6-12-week old male NSG mice,  Nalm6-GL, Raji cells (GFP-Luc+) | | Intravenous | | CD19 | | TRAC | | Produce allogeneic CAR-T cells while avoiding GVHD | Cas9 RNP | 95.7% | No | | 1. TCR KO CAR T cells showed similar efficacy to standard CAR T cells.  2. Mice treated with TCR KO CAR T cells showed no signs of GVHD. |
| (Wu, Giddings et al. 2024) 2024  Abstract | Leukemia, lymphoma, melanoma | | Murine CAR-T (mouse), human CAR-T (human) cells, mouse melanoma and leukemia tumor models | | NA | | CD19 | | DnaJC15 | | Increase efficacy | CRISPR-Cas9, shRNA | NA | | NA | MCJ deficiency enhances mitochondrial Complex I activity, increases mitochondrial membrane potential and respiration, improves CAR-T cytotoxicity and cytokine secretion, leads to improved tumor control and survival in murine leukemia and melanoma models. |
| (Xia, Peng et al. 2023) 2023  Abstract | Hematologic malignancies | | Human CAR-T; xenograft tumor models (mouse, NSG) | | NA | | CD19, BCMA | | TRAC | | Produce allogeneic CAR-T cells while avoiding GVHD | CRISPR-Cas9 | NA | | NA | Disruption of TCR or insertion of CAR at TRAC locus reduces CAR-T proliferation and tumor control due to impaired IL-2 production, while exogenous IL-2 rescues function. |
| (Yang, Li et al. 2021)  2021 | Oropharyngeal SCC | | 6-8-week-old female NSG mice,  FaDu | | Intravenous & intratumoral | | HER2 | | PD-1 | | Increase efficacy | CRISPR/Cas9 | N/A | | Atezolizumab (5 mg/kg twice weekly in both cases and controls) | Colonization of tumors by CAR T cells and animal survival was greater in the PD-1 KO compared to HER2 CARs.  PD-1 KO CARs led to higher IL-2 production (p< 0.02) & T cell expansion (p< 0.01) |
| (Yoshikawa, Wu et al. 2021)  2021 | ALL,  melanoma | | 4-10-week-old male NSG mice,  NALM6-GL,  K562-CD19,  A375-CD19 | | Intravenous | | CD19,  GD2,  mesothelin | | PRDM1 | | Increase efficacy | CRISPR/Cas9 | N/A | | No | 1. PRDM1-KO CAR-T cells showed stronger antitumor activity, expansion and persistence in both liquid and solid tumors and also when targeting antigens other than CD19.  2. Mice treated with PRDM1-KO CAR-T cells developed GVHD more often than controls. |
| (Yoshikawa, Wu et al. 2022) 2022 | Hematologic and solid tumors (leukemia, melanoma) | | Human primary T cells, TILs, mouse xenograft models (NSG mice) | | Intravenous | | CD19, GD2, Mesothelin, MART1 | | PRDM1 | | Increase efficacy | CRISPR-Cas9 | NA | | NA | PRDM1 knockout suppresses terminal differentiation, promotes early memory and cytokine polyfunctionality, enhances persistence and delayed but potent cytolytic activity in vivo, improves control of liquid and solid tumors. |
| (Zhang, Yao et al. 2024) 2024 | Prostate Cancer | | Human T cells; prostate cancer cell lines (PC-3, DU-145); mouse xenograft model | | Intravenous | | NKG2D | | HDAC11 | | Increase efficacy | shRNA | NA | | NA | HDAC11 knockdown CAR-T cells showed enhanced proliferation, reduced exhaustion markers, increased memory T cell formation, elevated cytotoxicity and cytokine secretion, and improved tumor control and mouse survival. |
| (Zhang, Ying et al. 2024) 2024 | B-ALL | | Human Nalm-6CD19pos and Nalm-6CD19neg cell lines; PBMC; NSG mice xenograft | | Intravenous | | CD19 | | IFNγ | | Decreasing CAR T cell toxicity | shRNA | NA | | NA | shIFN-γ-anti-CD19CAR-T cells significantly reduce IFN-γ, IL-6, TNF-α cytokine levels in in vivo, mitigate CRS-associated cytokine storms, preserve antitumor cytotoxicity comparable to regular anti-CD19CAR-T cells with dose adjustment. |
| (Zhang, Wu et al. 2024) 2024  Abstract | Leukemia | | Human T cells; Nalm-6 leukemia xenograft in NSG mice | | Intravenous | | CD19 | | IL2, IL2Rβ (prime-editing) | | Increase efficacy | CRISPR-Cas9 | >96% | | NA | OrthoIL-2 system with CAR knocked into IL2 locus and endogenous IL2Rβ replaced improves CAR T uniformity and potency, reduces tonic signaling, and shows superior tumor control in vivo. IL2 gene knockout does not impair CAR T function. |
| (Zhang, Zhang et al. 2022) 2022 | Solid tumors (pancreatic, mesothelioma, melanoma, lymphoma models) | | Human CAR-T cells targeting mesothelin, HER1, CD19; mouse OT-1 T cells; multiple cell lines and patient-derived xenograft (PDX) models; NSG and syngeneic mouse models | | Intravenous and intratumoral | | Mesothelin, HER1, CD19, OVA | | BATF | | Increase efficacy | CRISPR-Cas9 | NA | | NA | BATF knockout CAR-T cells show resistance to exhaustion, improved tumor killing with increased central memory CAR-T cell proportion, and superior and long-lasting antitumor activity across solid tumor models in vivo. |
| (Zhang, Zhang et al. 2017)  2017 | Lymphoma | | 6‒12-week-old NOD-Prkdcscid Il2rgnull (NPG) mice.  Raji-ffluc lymphomb a | | Intraperitoneal | | CD19 | | LAG-3 | | Increase efficacy | CRISPR-Cas9 | LAG-3 KO of 45-70% | | No | 1. Standard and LAG-3 KO CARs had similar tumor size but significantly smaller compared to controls.  2. CAR T cell-treated mice had longer survival. |
| (Zhou, Yu et al. 2021)  2021 | Lymphoma,  Prostate cancer | | 4–6-week-old NSG mice,  K562-CD19,  PC3-PSCA | | Intravenous (via tail vein) | | CD19,  PSCA | | PD-1 | | Increase efficacy | shRNA | CD19 plus PD-1 KO: 35.1%  PSCA plus PD-1 KO: also 35.1% | | No | 1. PD-1 KO improved survival, reduced immunosuppression and increased the anti-tumor activity of CAR-T cells.  2. CD19 PD-1 KOA CAR-T cells had slightly better efficacy and lower side effect compared to CD19 CAR-T plus PD-1 antibody. |
| (Zhu, You et al. 2020)  2020 | GBM | | BALB/c nude mice, U373 | | Intratumoral | | EGFRvIII | | PD-1 | | Increase efficacy | CRISPR/Cas9 | N/A | | No | PD-1 KD CAR-T cell-treated mice exhibited longer survival. |
| (Zhu, Li et al. 2024) 2024 | T-ALL | | Human γδT cells from PBMCs, T-ALL cell lines (CCRF-CEM, MOLT-4), NSG mice xenograft models | | Intravenous | | CD5 | | CD5 | | 1. limit fratricide  2. Increase efficacy | CRISPR/Cas9 | ~90% | | NA | Constructed a CD5 nanobody (CD5-27 Nb)-based CAR on CRISPR-edited γδTCD5− cells via mRNA electroporation; fratricide was prevented by CD5 knockout and mRNA-engineered CD5-CAR-γδTCD5− cells demonstrated high CAR expression, potent and specific cytotoxicity to CD5+ malignant T-ALL cells in vivo. |
| (Zou, Liu et al. 2022)  2022 | Breast cancer,  Glioblastoma | | Xenograft models of breast cancer and glioblastoma | | N/A | | HER2 (breast)  CD133 (glioblastoma) | | IKZF3 | | Increase efficacy | CRISPR/Cas9 | N/A | | No | 1. IKZF3 KO increased T cell proliferation and activation, enhanced tumor killing.  2. IKZF3 KO led to elevated expression of genes in chemotaxis, cytokine signaling, and cytotoxicity. |

* Studies mentioned as “Abstract” are abstracts and do not contain full texts

Abbreviations: β2M: Beta-2 microglobulin, TRAC: TCRα subunit constant, GVHD: Graft-versus-host disease, CRISPR-Cas9: Clustered regularly interspaced short palindromic repeats- CRISPR-associated protein 9, DKO/TKO: Double/triple knockout, BCMA: B-cell maturation antigen, TCR: T-cell receptor, shRNA: Short hairpin RNA, N/A: Not available/applicable, CNS: Central nervous system, GBM: Glioblastoma multiforme, EGFRvIII: Epidermal growth factor receptor variant III, TALEN: Transcription activator-like effector nucleases, NPG: NOD-Prkdc scid Il2rgnull, SHP2: Src homology region 2-containing protein tyrosine phosphatase 2, LAG-3: Lymphocyte activation gene-3, ALL: Acute lymphoblastic leukemia, OS: Overall survival, DNMT3A: DNA methyltransferase 3 alpha, RCC: Renal cell carcinoma, AML: Acute myeloid leukemia, MM: Multiple myeloma, A2AR: Adenosine A2A receptor, DGK: diacylglycerol kinase, HCC: Hepatocellular carcinoma, IDO1: Indoleamine-2,3-dioxygenase 1, EpCAM: anti-epithelial cell adhesion molecule, SCC: Squamous cell carcinoma, CBLB: Casitas B-lineage lymphoma proto-oncogene-b, NOG: NOD.Cg-Prkdcscid Il2rgtm1Sug/JicTac, PD-1H: Programmed death-1 homolog, LA: low affinity, HA: high affinity, BL: Burkitt's lymphoma, TME: tumor microenvironment, DIPG: diffuse intrinsic pontine glioma.

**Table 2.** Risk of bias assessment for the included animal studies using the SYRCLE’s tool (Hooijmans, Rovers et al. 2014). Each item is allocated with Yes (Yes) for proper addressing, No (No) for insufficient addressing, or unlcear (N/A) addressing of risk of bias.

| Study | Selection bias | | | Performance bias | | Detection bias | | Attrition bias | Reporting bias | Other |
| --- | --- | --- | --- | --- | --- | --- | --- | --- | --- | --- |
|  | Sequence generation | Baseline characteristics | Allocation concealement | Random housing | Blinding | Random outcome assessment | Blinding | Incomplete outcome data | Selective outcome reporting | Other sources of bias |
| (Adachi, Terakura et al. 2024) | Yes | Yes | N/A | N/A | N/A | N/A | N/A | N/A | Yes | Yes |
| (Adeshakin, Zhou et al. 2023) | Yes | Yes | Yes | N/A | N/A | N/A | N/A | Yes | Yes | N/A |
| (Agarwal, Aznar et al. 2023) | Yes | Yes | N/A | N/A | N/A | N/A | N/A | N/A | N/A | N/A |
| (Agarwal 2020) | N/A | N/A | N/A | N/A | N/A | N/A | N/A | Yes | Yes | Yes |
| (Ajmal, Farooq et al. 2024) | Yes | N/A | N/A | N/A | No | N/A | N/A | N/A | N/A | N/A |
| (Alizadeh, Nee et al. 2023) | N/A | N/A | N/A | N/A | N/A | N/A | N/A | N/A | N/A | N/A |
| (An, Pan et al. 2023) | N/A | N/A | N/A | N/A | N/A | N/A | N/A | N/A | N/A | N/A |
| (Andreu-Saumell, Rodriguez-Garcia et al. 2024) | N/A | Yes | N/A | N/A | N/A | N/A | N/A | Yes | N/A | Yes |
| (Angelos, Patel et al. 2023) | N/A | N/A | N/A | N/A | N/A | N/A | N/A | N/A | N/A | N/A |
| (Bachl, Talbot et al. 2023) | N/A | N/A | N/A | N/A | N/A | N/A | N/A | N/A | N/A | N/A |
| (Bailey, Takei et al. 2025) | Yes | Yes | N/A | N/A | N/A | N/A | N/A | N/A | Yes | Yes |
| (Bailey, Vatsa et al. 2022) | N/A | N/A | N/A | N/A | N/A | N/A | N/A | N/A | N/A | Yes |
| (Balakrishnan, Leach et al. 2023) | N/A | N/A | N/A | N/A | N/A | N/A | N/A | N/A | N/A | N/A |
| (Battram, Mane-Pujol et al. 2025) | Yes | N/A | Yes | N/A | N/A | N/A | N/A | N/A | N/A | Yes |
| (Beavis, Henderson et al. 2017) | N/A | Yes | N/A | N/A | N/A | N/A | N/A | Yes | Yes | N/A |
| (Beckett, Chockley et al. 2023) | N/A | N/A | N/A | N/A | N/A | N/A | N/A | N/A | N/A | N/A |
| (Benton, Liu et al. 2025) | N/A | Yes | N/A | N/A | N/A | N/A | N/A | Yes | N/A | Yes |
| (Bolaños, Calviño et al. 2023) | N/A | N/A | N/A | N/A | N/A | N/A | N/A | N/A | N/A | N/A |
| (Braun, Pruene et al. 2023) | N/A | Yes | N/A | N/A | N/A | N/A | N/A | Yes | Yes | Yes |
| (Bridge, Johnson et al. 2024) | N/A | Yes | N/A | N/A | N/A | N/A | N/A | Yes | N/A | Yes |
| (Cai, Gouble et al. 2022) | N/A | N/A | N/A | N/A | N/A | N/A | N/A | N/A | N/A | Yes |
| (Calderon, Fleming et al. 2023) | N/A | N/A | N/A | N/A | N/A | N/A | N/A | N/A | N/A | N/A |
| (Calviño, Ceballos et al. 2023) | N/A | Yes | N/A | N/A | N/A | N/A | N/A | Yes | N/A | N/A |
| (Cappabianca, Pham et al. 2024) | N/A | N/A | N/A | N/A | Yes | N/A | Yes | Yes | Yes | N/A |
| (Carnevale, Shifrut et al. 2022) | N/A | Yes | N/A | N/A | N/A | N/A | N/A | N/A | N/A | N/A |
| (Certo, Kurtulus et al. 2023) | N/A | N/A | N/A | N/A | N/A | N/A | N/A | N/A | N/A | N/A |
| (Chan, Bolinger et al. 2022) | Yes | Yes | N/A | N/A | N/A | N/A | N/A | Yes | Yes | N/A |
| (Chang, Van Der Stegen et al. 2019) | N/A | N/A | N/A | N/A | N/A | N/A | N/A | N/A | Yes | Yes |
| (Chang, Peralta et al. 2020) | N/A | N/A | N/A | N/A | N/A | N/A | N/A | Yes | Yes | N/A |
| (Chen, López-Moyado et al. 2019) | N/A | N/A | N/A | N/A | N/A | N/A | N/A | N/A | N/A | Yes |
| (Chen, Yap et al. 2025) | N/A | Yes | N/A | N/A | N/A | N/A | N/A | Yes | Yes | Yes |
| (Chen, Deng et al. 2024) | Yes | N/A | N/A | N/A | N/A | N/A | N/A | N/A | Yes | N/A |
| (Chen, Tan et al. 2024) | N/A | Yes | N/A | N/A | N/A | N/A | N/A | Yes | Yes | N/A |
| (Cheng, Zhang et al. 2025) | N/A | N/A | N/A | N/A | N/A | N/A | N/A | Yes | Yes | N/A |
| (Cherkassky, Morello et al. 2016) | N/A | Yes | N/A | N/A | N/A | N/A | N/A | Yes | Yes | Yes |
| (Choi, Yu et al. 2019) | N/A | N/A | N/A | N/A | No | N/A | No | Yes | Yes | N/A |
| (Choi, Yu et al. 2020) | Yes | N/A | N/A | N/A | No | N/A | N/A | N/A | N/A | N/A |
| (Chu, Nava-Barbero et al. 2024) | Yes | Yes | No | N/A | N/A | N/A | N/A | Yes | Yes | N/A |
| (Chun, Kim et al. 2020) | N/A | N/A | N/A | N/A | N/A | N/A | N/A | Yes | Yes | N/A |
| (Condomines, Arnason et al. 2015) | N/A | Yes | N/A | N/A | No | No | No | Yes | Yes | Yes |
| (Cooper, Choi et al. 2018) | N/A | N/A | N/A | N/A | N/A | N/A | N/A | N/A | N/A | Yes |
| (Cooper, Staser et al. 2018) | N/A | N/A | N/A | N/A | N/A | N/A | N/A | N/A | N/A | Yes |
| (Cox, Manriquez Roman et al. 2022) | N/A | Yes | N/A | N/A | N/A | N/A | N/A | Yes | Yes | N/A |
| (Dai, Mu et al. 2022) | N/A | N/A | N/A | N/A | N/A | N/A | N/A | Yes | Yes | N/A |
| (Dai, Mu et al. 2021) | N/A | N/A | N/A | N/A | N/A | N/A | N/A | Yes | N/A | Yes |
| (Das, Valton et al. 2023) | Yes | Yes | N/A | N/A | N/A | N/A | N/A | Yes | Yes | Yes |
| (David, Schiele et al. 2025) | N/A | N/A | N/A | N/A | N/A | N/A | N/A | Yes | Yes | Yes |
| (Dawn Sakthi Vale, Wu et al. 2022) | N/A | N/A | N/A | N/A | N/A | N/A | N/A | N/A | N/A | N/A |
| (De Munter, Buhl et al. 2024) | N/A | N/A | N/A | N/A | N/A | N/A | N/A | N/A | Yes | Yes |
| (Deeren, Maertens et al. 2020) | N/A | N/A | N/A | N/A | N/A | N/A | N/A | Yes | No | N/A |
| (Degagné, Donohoue et al. 2024) | N/A | N/A | N/A | N/A | N/A | N/A | N/A | N/A | N/A | N/A |
| (Dequeant, Sagert et al. 2021) | N/A | N/A | N/A | N/A | Yes | N/A | Yes | Yes | Yes | N/A |
| (Dharani, Cho et al. 2024) | N/A | Yes | N/A | N/A | N/A | N/A | N/A | Yes | Yes | Yes |
| (Diorio, Murray et al. 2022) | N/A | Yes | N/A | N/A | N/A | N/A | N/A | Yes | Yes | Yes |
| (Dörr, Gregor et al. 2024) | N/A | N/A | N/A | N/A | N/A | N/A | N/A | N/A | N/A | N/A |
| (Dötsch, Svec et al. 2023) | N/A | Yes | N/A | N/A | N/A | N/A | N/A | Yes | Yes | Yes |
| (Drake, Garcia et al. 2022) | N/A | N/A | N/A | N/A | N/A | N/A | N/A | N/A | N/A | N/A |
| (Engel, Steinfeld et al. 2025) | N/A | Yes | N/A | N/A | N/A | N/A | N/A | Yes | Yes | Yes |
| (Evtimov, Nguyen et al. 2025) | N/A | Yes | N/A | N/A | N/A | N/A | N/A | Yes | Yes | Yes |
| (Fraessle, Tschulik et al. 2023) | N/A | N/A | N/A | N/A | N/A | N/A | N/A | Yes | Yes | Yes |
| (Freitas, Belk et al. 2022) | N/A | Yes | N/A | N/A | N/A | N/A | N/A | Yes | Yes | Yes |
| (Fu, Huang et al. 2024) | N/A | N/A | N/A | N/A | N/A | N/A | N/A | Yes | Yes | Yes |
| (Gagnon, Litterman et al. 2022) | N/A | Yes | N/A | N/A | N/A | N/A | N/A | N/A | Yes | Yes |
| (Garner, Kelly et al. 2023) | N/A | N/A | N/A | N/A | N/A | N/A | N/A | N/A | N/A | N/A |
| (Georgiadis, Rasaiyaah et al. 2020) | N/A | N/A | N/A | N/A | N/A | N/A | N/A | N/A | N/A | N/A |
| (Georgiadis, Preece et al. 2018) | N/A | N/A | N/A | N/A | N/A | N/A | N/A | N/A | N/A | N/A |
| (Georgiadis, Nickolay et al. 2024) | N/A | Yes | N/A | N/A | N/A | N/A | N/A | Yes | Yes | Yes |
| (Giuffrida, Sek et al. 2021) | Yes | N/A | Yes | N/A | N/A | N/A | N/A | Yes | Yes | N/A |
| (Goala, Davila et al. 2025) | N/A | Yes | N/A | N/A | N/A | N/A | N/A | Yes | Yes | Yes |
| (Gomes-Silva, Atilla et al. 2019) | N/A | N/A | N/A | N/A | N/A | N/A | N/A | N/A | N/A | Yes |
| (Goto, Ishii et al. 2024) | N/A | N/A | N/A | N/A | N/A | N/A | N/A | N/A | N/A | N/A |
| (Gouble, Galetto et al. 2018) | N/A | N/A | N/A | N/A | N/A | N/A | N/A | N/A | Yes | N/A |
| (Gouble, Philip et al. 2014) | N/A | N/A | N/A | N/A | N/A | N/A | N/A | N/A | N/A | N/A |
| (Gough, Georgiadis et al. 2025) | N/A | N/A | N/A | N/A | N/A | N/A | N/A | N/A | Yes | Yes |
| (Graham, Jozwik et al. 2021) | N/A | N/A | N/A | N/A | N/A | N/A | N/A | N/A | N/A | Yes |
| (Grauwet, Berger et al. 2024) | N/A | Yes | N/A | N/A | N/A | N/A | N/A | Yes | N/A | Yes |
| (Gregor, Dörr et al. 2025) | N/A | N/A | N/A | N/A | N/A | N/A | N/A | N/A | N/A | N/A |
| (Guo, Jiang et al. 2018) | Yes | Yes | No | N/A | N/A | N/A | N/A | Yes | Yes | Yes |
| (Guruprasad, Carturan et al. 2024) | N/A | Yes | N/A | N/A | N/A | Yes | Yes | N/A | Yes | Yes |
| (Guzman, Sugita et al. 2016) | N/A | Yes | N/A | N/A | N/A | N/A | N/A | Yes | Yes | Yes |
| (Ho, Yu et al. 2023) | N/A | N/A | N/A | N/A | N/A | N/A | N/A | N/A | N/A | N/A |
| (Hooper, Havens et al. 2018) | N/A | N/A | N/A | N/A | N/A | N/A | N/A | N/A | N/A | N/A |
| (Hu, Wang et al. 2022) | N/A | Yes | N/A | N/A | N/A | N/A | N/A | Yes | Yes | N/A |
| (Hu, Manner et al. 2023) | N/A | Yes | N/A | N/A | N/A | Yes | N/A | Yes | Yes | Yes |
| (Hu, McAlister et al. 2025) | N/A | Yes | N/A | N/A | N/A | Yes | N/A | Yes | Yes | Yes |
| (Hu, Zi et al. 2019) | Yes | Yes | No | N/A | N/A | N/A | N/A | Yes | Yes | N/A |
| (Hu, Zou et al. 2019) | Yes | N/A | N/A | N/A | N/A | N/A | N/A | Yes | N/A | Yes |
| (Hu, Yi et al. 2020) | N/A | N/A | N/A | N/A | N/A | N/A | N/A | N/A | N/A | N/A |
| (Hu, Chen et al. 2022) | N/A | N/A | N/A | N/A | N/A | N/A | N/A | N/A | N/A | Yes |
| (Jambon, Ruiz et al. 2024) | N/A | N/A | N/A | N/A | N/A | N/A | N/A | N/A | N/A | N/A |
| (Jiang, Chen et al. 2023) | N/A | N/A | N/A | N/A | N/A | Yes | N/A | N/A | Yes | N/A |
| (Jing, Scarfo et al. 2022) | N/A | Yes | N/A | N/A | N/A | Yes | N/A | N/A | No | N/A |
| (Jo, Das et al. 2022) | N/A | Yes | N/A | N/A | N/A | Yes | N/A | N/A | Yes | Yes |
| (Jung, Narayan et al. 2022) | N/A | N/A | N/A | N/A | No | N/A | N/A | Yes | Yes | Yes |
| (Jung, Bartoszek et al. 2023) | N/A | Yes | N/A | N/A | N/A | N/A | N/A | Yes | Yes | Yes |
| (Jung, Kim et al. 2018) | Yes | N/A | Yes | N/A | N/A | N/A | N/A | Yes | Yes | N/A |
| (Kagoya, Guo et al. 2020) | N/A | Yes | N/A | N/A | N/A | N/A | N/A | Yes | Yes | Yes |
| (Kath, Du et al. 2022) | N/A | N/A | N/A | N/A | N/A | N/A | N/A | N/A | Yes | N/A |
| (Kath, Du et al. 2022) | N/A | N/A | N/A | N/A | N/A | N/A | N/A | Yes | Yes | Yes |
| (Kalaitzidis, Henderson et al. 2019) | N/A | N/A | N/A | N/A | N/A | N/A | N/A | Yes | Yes | N/A |
| (Kelly, Sanchez-Pupo et al. 2023) | N/A | N/A | N/A | N/A | N/A | N/A | N/A | N/A | Yes | N/A |
| (Keerthi, Balke-Want et al. 2024) | N/A | N/A | N/A | N/A | N/A | N/A | N/A | N/A | Yes | N/A |
| (Kienka, Korell et al. 2024) | N/A | N/A | N/A | N/A | N/A | N/A | N/A | N/A | Yes | Yes |
| (Kim, Jeun et al. 2025) | N/A | N/A | N/A | N/A | N/A | N/A | N/A | Yes | Yes | N/A |
| (Kinder, Athena-Estrada et al. 2022) | N/A | N/A | N/A | N/A | N/A | N/A | N/A | N/A | Yes | N/A |
| (Koo, Jeong et al. 2025) | N/A | N/A | N/A | N/A | N/A | N/A | N/A | N/A | Yes | N/A |
| (Korst, O'Neill et al. 2024) | N/A | N/A | N/A | N/A | N/A | N/A | N/A | Yes | Yes | Yes |
| (Lam, Barragan et al. 2022) | N/A | N/A | N/A | N/A | N/A | N/A | N/A | N/A | N/A | N/A |
| (Lam, Li et al. 2023) | N/A | N/A | N/A | N/A | N/A | N/A | N/A | N/A | Yes | N/A |
| (Lau, Kwong et al. 2023) | N/A | N/A | N/A | N/A | N/A | N/A | N/A | N/A | Yes | N/A |
| (Lee, Lee et al. 2022) | N/A | Yes | N/A | N/A | N/A | N/A | N/A | Yes | N/A | N/A |
| (Lei, Li et al. 2024) | N/A | N/A | N/A | N/A | N/A | N/A | N/A | Yes | Yes | Yes |
| (Li, Su et al. 2024) | N/A | N/A | N/A | N/A | N/A | N/A | N/A | N/A | Yes | N/A |
| (Li, Zhu et al. 2022) | N/A | N/A | N/A | N/A | N/A | N/A | N/A | Yes | Yes | Yes |
| (Li, Tang et al. 2020) | N/A | Yes | N/A | N/A | N/A | N/A | N/A | Yes | Yes | Yes |
| (Lin, Yamada-Hunter et al. 2025) | N/A | N/A | N/A | N/A | N/A | N/A | N/A | N/A | Yes | N/A |
| (Liu, Zhang et al. 2021) | N/A | N/A | N/A | N/A | N/A | N/A | N/A | Yes | Yes | N/A |
| (Liu, Zhang et al. 2017) | N/A | N/A | N/A | N/A | N/A | N/A | N/A | N/A | N/A | N/A |
| (Lonez, Bolsée et al. 2025) | N/A | N/A | N/A | N/A | N/A | N/A | N/A | Yes | Yes | N/A |
| (Lu, Chu et al. 2025) | N/A | Yes | Yes | N/A | N/A | N/A | N/A | Yes | Yes | Yes |
| (Lu, C Lam et al. 2024) | N/A | N/A | N/A | N/A | N/A | N/A | N/A | N/A | Yes | Yes |
| (Lu, Li et al. 2024) | N/A | Yes | N/A | N/A | N/A | N/A | N/A | Yes | Yes | Yes |
| (Lu, Yang et al. 2024) | N/A | Yes | N/A | N/A | N/A | N/A | N/A | Yes | Yes | Yes |
| (MacLeod, Antony et al. 2017) | N/A | Yes | N/A | N/A | N/A | N/A | N/A | Yes | Yes | Yes |
| (Mai, Boyce et al. 2024) | N/A | Yes | N/A | N/A | N/A | N/A | N/A | Yes | Yes | Yes |
| (Maldini, Messana et al. 2024) | N/A | Yes | Yes | N/A | N/A | N/A | N/A | Yes | Yes | Yes |
| (Maldonado-Pérez, Tristán-Manzano et al. 2022) | N/A | N/A | N/A | N/A | N/A | N/A | N/A | Yes | Yes | N/A |
| (Mathur, Zhang et al. 2018) | N/A | N/A | N/A | N/A | N/A | N/A | N/A | N/A | N/A | N/A |
| (Mavuluri 2022) | N/A | N/A | N/A | N/A | N/A | N/A | N/A | N/A | N/A | N/A |
| (McEwan, Padalia et al. 2019) | N/A | N/A | N/A | N/A | N/A | N/A | N/A | N/A | N/A | Yes |
| (Menegatti, Lopez-Cobo et al. 2024) | N/A | Yes | Yes | N/A | N/A | N/A | N/A | Yes | Yes | Yes |
| (Mise-Omata, Ando et al. 2023) | N/A | Yes | N/A | N/A | N/A | N/A | N/A | Yes | Yes | Yes |
| (Nakashima, Ouchida et al. 2025) | N/A | Yes | Yes | N/A | N/A | N/A | N/A | Yes | Yes | Yes |
| (Nakagawara, Ando et al. 2024) | N/A | Yes | N/A | N/A | N/A | N/A | N/A | Yes | Yes | N/A |
| (Oumzil, Guerrero et al. 2022) | N/A | N/A | N/A | N/A | N/A | N/A | N/A | N/A | N/A | N/A |
| (Ouyang, Jin et al. 2024) | N/A | N/A | N/A | N/A | N/A | N/A | N/A | Yes | Yes | Yes |
| (Pal, Tran et al. 2024) | N/A | N/A | N/A | N/A | N/A | N/A | N/A | Yes | Yes | Yes |
| (Panowski, Srinivasan et al. 2022) | N/A | N/A | N/A | N/A | N/A | N/A | N/A | N/A | No | N/A |
| (Park, Monroy et al. 2022) | N/A | N/A | N/A | N/A | N/A | N/A | N/A | N/A | Yes | N/A |
| (Patel, Ghilardi et al. 2022) | N/A | N/A | N/A | N/A | N/A | N/A | N/A | N/A | Yes | N/A |
| (Patel, Ghilardi et al. 2024) | N/A | Yes | N/A | N/A | N/A | N/A | N/A | Yes | Yes | Yes |
| (Peng, Yao et al. 2025) | N/A | N/A | N/A | N/A | N/A | N/A | N/A | N/A | Yes | N/A |
| (Petersen, Bell et al. 2019) | N/A | N/A | N/A | N/A | N/A | N/A | N/A | N/A | N/A | N/A |
| (Poirot, Philip et al. 2015) | N/A | N/A | N/A | N/A | N/A | N/A | N/A | N/A | N/A | N/A |
| (Preece, Gough et al. 2025) | N/A | N/A | N/A | N/A | N/A | N/A | N/A | N/A | Yes | N/A |
| (Prinzing, Zebley et al. 2021) | N/A | N/A | N/A | N/A | N/A | N/A | N/A | N/A | N/A | Yes |
| (Qiao, Chen et al. 2023) | N/A | Yes | N/A | N/A | N/A | N/A | N/A | Yes | Yes | Yes |
| (Rasaiyaah, Georgiadis et al. 2018) | N/A | Yes | N/A | N/A | N/A | N/A | No | Yes | Yes | N/A |
| (Ren, Liu et al. 2017) | N/A | N/A | N/A | N/A | N/A | N/A | N/A | N/A | N/A | Yes |
| (Ren, Liao et al. 2024) | N/A | Yes | Yes | N/A | N/A | N/A | N/A | Yes | Yes | N/A |
| (Richter, Cranert et al. 2019) | N/A | N/A | N/A | N/A | N/A | N/A | N/A | N/A | N/A | N/A |
| (Roders, Nakid-Cordero et al. 2024) | N/A | Yes | Yes | N/A | N/A | N/A | N/A | Yes | Yes | Yes |
| (Rupp, Schumann et al. 2017) | N/A | N/A | N/A | N/A | N/A | N/A | N/A | Yes | Yes | N/A |
| (Salim, Shaikh et al. 2023) | N/A | Yes | N/A | N/A | N/A | N/A | N/A | Yes | Yes | N/A |
| (Sanber, Nawas et al. 2020) | N/A | N/A | N/A | N/A | N/A | N/A | N/A | N/A | N/A | N/A |
| (Schiffer-Mannioui, Leduc et al. 2021) | N/A | N/A | N/A | N/A | N/A | N/A | N/A | N/A | N/A | N/A |
| (Senz, Metzger et al. 2020) | N/A | Yes | N/A | N/A | N/A | N/A | N/A | Yes | Yes | Yes |
| (Shang, Huang et al. 2025) | N/A | Yes | N/A | N/A | N/A | N/A | N/A | Yes | Yes | N/A |
| (Shi, Zhang et al. 2025) | N/A | Yes | N/A | N/A | N/A | N/A | N/A | Yes | Yes | Yes |
| (Shirinbak, Grant et al. 2022) | N/A | N/A | N/A | N/A | N/A | N/A | N/A | N/A | Yes | N/A |
| (Sodji, Shea et al. 2025) | N/A | Yes | N/A | N/A | N/A | N/A | N/A | Yes | Yes | Yes |
| (Sommer, Boldajipour et al. 2019) | N/A | Yes | N/A | N/A | N/A | N/A | N/A | Yes | Yes | N/A |
| (Soltantoyeh, Akbari et al. 2024) | N/A | Yes | Yes | N/A | N/A | N/A | N/A | Yes | Yes | N/A |
| (Stenger, Stief et al. 2020) | N/A | Yes | N/A | N/A | N/A | N/A | N/A | Yes | Yes | Yes |
| (Stepanova, Volkov et al. 2024) | N/A | Yes | Yes | N/A | N/A | N/A | N/A | Yes | Yes | N/A |
| (Sterner, Sakemura et al. 2019) | N/A | Yes | N/A | N/A | N/A | N/A | N/A | Yes | Yes | Yes |
| (Stewart, Siegler et al. 2024) | N/A | Yes | N/A | N/A | N/A | N/A | N/A | Yes | Yes | Yes |
| (Su, Yao et al. 2024) | N/A | Yes | Yes | N/A | N/A | N/A | N/A | Yes | Yes | N/A |
| (Sugita, Galetto et al. 2022) | N/A | Yes | N/A | N/A | N/A | N/A | N/A | Yes | Yes | N/A |
| (Tang, Liu et al. 2024) | N/A | Yes | Yes | N/A | N/A | N/A | N/A | Yes | Yes | Yes |
| (Tang, Cheng et al. 2020) | Yes | Yes | No | N/A | No | N/A | No | Yes | Yes | Yes |
| (Tipanee, Samara-Kuko et al. 2022) | N/A | Yes | N/A | N/A | N/A | N/A | N/A | Yes | Yes | Yes |
| (Trefny, Kirchhammer et al. 2023) | N/A | Yes | N/A | N/A | N/A | N/A | N/A | Yes | Yes | N/A |
| (Vale and Gascoigne 2025) | N/A | N/A | N/A | N/A | N/A | N/A | N/A | N/A | N/A | N/A |
| (Wang, Wu et al. 2025) | N/A | Yes | Yes | N/A | N/A | N/A | N/A | Yes | Yes | Yes |
| (Wang, Li et al. 2021) | N/A | N/A | N/A | N/A | N/A | N/A | N/A | Yes | N/A | Yes |
| (Wang, Prager et al. 2021) | N/A | N/A | N/A | N/A | N//A | N/A | N/A | Yes | Yes | N/A |
| (Ward, Senaoui et al. 2024) | N/A | N/A | N/A | N/A | N/A | N/A | N/A | N/A | Yes | N/A |
| (Ward, Adeshakin et al. 2024) | N/A | N/A | N/A | N/A | N/A | N/A | N/A | N/A | Yes | N/A |
| (Wei, Luo et al. 2019) | Yes | Yes | N/A | N/A | N/A | No | N/A | Yes | N/A | N/A |
| (Wellhausen, O'Connell et al. 2023) | N/A | Yes | N/A | N/A | N/A | N/A | N/A | Yes | Yes | Yes |
| (Wen, Huo et al. 2021) | Yes | N/A | N/A | N/A | No | N/A | Yes | N/A | N/A | N/A |
| (Wiebking, Lee et al. 2021) | N/A | Yes | N/A | N/A | N/A | N/A | N/A | Yes | Yes | Yes |
| (Williams, Mbofung et al. 2024) | N/A | N/A | N/A | N/A | N/A | N/A | N/A | N/A | Yes | Yes |
| (Wu, Giddings et al. 2024) | N/A | N/A | N/A | N/A | N/A | N/A | N/A | N/A | Yes | N/A |
| (Xia, Peng et al. 2023) | N/A | N/A | N/A | N/A | N/A | N/A | N/A | N/A | N/A | N/A |
| (Yang, Li et al. 2021) | N/A | N/A | N/A | N/A | No | N/A | No | No | No | N/A |
| (Yoshikawa, Wu et al. 2021) | N/A | Yes | N/A | N/A | N/A | N/A | N/A | Yes | Yes | Yes |
| (Yoshikawa, Wu et al. 2022) | N/A | Yes | N/A | N/A | N/A | N/A | N/A | Yes | Yes | Yes |
| (Zhang, Zhang et al. 2022) | N/A | Yes | Yes | N/A | N/A | N/A | N/A | Yes | Yes | Yes |
| (Zhang, Zhang et al. 2017) | Yes | Yes | N/A | N/A | No | N/A | No | Yes | Yes | Yes |
| (Zhang, Yao et al. 2024) | N/A | Yes | N/A | N/A | N/A | N/A | N/A | Yes | Yes | N/A |
| (Zhang, Ying et al. 2024) | N/A | Yes | N/A | N/A | N/A | N/A | N/A | Yes | Yes | N/A |
| (Zhang, Wu et al. 2024) | N/A | N/A | N/A | N/A | N/A | N/A | N/A | N/A | Yes | N/A |
| (Zhou, Yu et al. 2021) | N/A | Yes | N/A | N/A | N/A | N/A | N/A | Yes | Yes | N/A |
| (Zhu, Li et al. 2024) | N/A | Yes | N/A | N/A | N/A | N/A | N/A | Yes | Yes | N/A |
| (Zhu, You et al. 2020) | Yes | Yes | N/A | N/A | N/A | N/A | N/A | Yes | Yes | Yes |
| (Zou, Liu et al. 2022) | N/A | Yes | N/A | N/A | N/A | N/A | N/A | Yes | Yes | Yes |

**Table 3.** Risk of bias assessment for the included clinical studies using National Heart, Lung, and Blood Institute (NHLBI)’s study quality assessment tools . This quality assessment tool gives responses as yes (Y), no (N), or Other (cannot determine (CD), not applicable (NA), or not reported (NR)). As this checklist comprise tools for several types of studies with different number of questions (e.g. 12 questions for pre-post studies with no control groups and nine for case series), if a row did not reach Q14, we would mention the non-existence of these questions with a dash “-“ sign.

The incomplete clinical trials (recruiting, no published data, etc.) are mentioned as incomplete and did not undergo quality assessment.

| Study | Q1 | Q2 | Q3 | Q4 | Q5 | Q6 | Q7 | Q8 | Q9 | Q10 | Q11 | Q12 | Q13 | Q14 |
| --- | --- | --- | --- | --- | --- | --- | --- | --- | --- | --- | --- | --- | --- | --- |
| NCT03545815  (Wang, Li et al. 2021) | Y | Y | Y | N | N | Y | Y | N | Y | N | N | Y | - | - |
| NCT03747965 (Wang, Chen et al. 2020) | Y | Y | Y | NR | N | Y | Y | N | Y | N | N | Y | - | - |
| (Kang, Tang et al. 2019) | NR | NR | NR | NR | NR | Y | Y | Y | NR | NR | Y | NR | Y | NR |
| NCT04150497  (Jain, Roboz et al. 2021) | Y | Y | Y | N | N | Y | Y | N | Y | N | N | Y | - | - |
| NCT04416984  (Lekakis, Locke et al. 2021) | Y | Y | Y | N | N | Y | Y | N | Y | N | N | Y | - | - |
| NCT03939026  (Neelapu, Nath et al. 2021) | Y | Y | Y | N | Y | Y | Y | N | Y | N | N | Y | - | - |
| NCT03525782  (Chen, Lin et al. 2018) | Y | Y | Y | N | N | Y | Y | N | Y | N | N | Y | - | - |
| (Fraietta, Nobles et al. 2018) | Y | Y | Y | NA | Y | Y | Y | Y | Y | - | - | - | - | - |
| (Mao and Xu 2021) | Y | Y | Y | Y | Y | Y | Y | N | Y | - | - | - | - | - |
| NCT02746952  NCT02808442  (Benjamin, Graham et al. 2020) | Y | Y | Y | N | N | Y | Y | N | Y | N | N | Y | - | - |
| (Qasim, Zhan et al. 2017) | Y | Y | N | Y | Y | Y | Y | NA | Y | - | - | - | - | - |
| (Ma, Dai et al. 2022) | Y | Y | NR | Y | Y | N | Y | NA | Y | - | - | - | - | - |
| (Hu, Yi et al. 2020) | Y | N | Y | NR | N | Y | Y | N | Y | N | N | Y | - | - |
| NCT01082926  (Brown, Rodriguez et al. 2022) | Y | Y | Y | NR | N | Y | Y | N | Y | N | N | Y | - | - |
| NCT03064269  (Chen, Kang et al. 2020) | Y | Y | Y | NR | N | Y | Y | N | Y | N | N | Y | - | - |
| NCT04167696  (Deeren, Maertens et al. 2020) | Y | Y | Y | NR | N | Y | Y | N | Y | N | N | Y | - | - |
| (Guo, Tong et al. 2022) | Y | Y | NR | Y | Y | Y | Y | NA | Y | - | - | - | - | - |
| NCT04227015  (Hu, Zhou et al. 2021) | Y | Y | Y | N | N | Y | Y | N | Y | N | N | Y | - | - |
| Part of NCT03919240  (Liu, Chen et al. 2020) | Y | Y | NA | Y | Y | Y | Y | NA | Y | - | - | - | - | - |
| NCT04093596  (Mailankody, Liedtke et al. 2021) | Y | Y | Y | N | Y | Y | Y | N | Y | N | N | Y | - | - |
| NCT04035434 | N | NA | NA | N | N | NR | N | Y | NR | N | Y | N | Y | NA |
| NCT04035434 (McGuirk, Tam et al. 2022) | N | NA | NA | N | N | N | N | Y | NR | N | Y | N | Y | NA |
| NCT02746952 (Benjamin, Jain et al. 2022) | N | NA | NA | N | N | N | Y | Y | Y | N | Y | N | Y | NA |
| ChiCTR1800020306 (Cen, Ke et al. 2022) | N | NA | NA | N | N | NA | NR | NA | NR | NR | Y | N | CD | NA |
| NCT03275493 (Gong, Qiu et al. 2022) | N | NA | NA | N | N | NA | Y | NA | Y | Y | Y | Y | Y | NA |
| (Guo, Tong et al. 2022) | Y | Y | CD | Y | Y | Y | N | NA | Y | - | - | - | - | - |
| NCT04637763 (Harcha, Hart et al. 2022) | N | NA | NA | N | N | NA | NR | NA | Y | Y | Y | N | Y | NA |
| NCT04557436 (Ottaviano, Georgiadis et al. 2022) | N | NA | NA | N | N | NA | Y | NA | Y | NA | Y | N | Y | NA |
| NCT04106076 (Sallman, DeAngelo et al. 2022) | N | NA | N | N | N | NR | NR | NR | Y | NR | Y | N | Y | NA |
| NCT04938115  NCT04916860 (Yang, Li et al. 2022) | N | NA | NA | N | N | NA | NR | NA | Y | NR | Y | N | Y | NA |
| NCT04823091 (Zhang, Luo et al. 2022) | N | NA | NA | N | N | NA | NR | NA | Y | NR | Y | N | Y | NA |
| (Baptista and Carvalho 2023) | N | NA | NA | N | N | NA | NR | NA | Y | NR | Y | N | Y | NA |
| NCT03706326 (Chen and Lin 2023) | N | NA | NA | N | N | NA | NR | NA | Y | NR | Y | N | Y | NA |
| NCT05397184 (Chiesa, Georgiadis et al. 2023) | N | NA | NA | N | N | NA | NR | NA | Y | NR | Y | N | Y | NA |
| NCT04960579 (Dholaria, Kocoglu et al. 2023) | N | NA | NA | N | N | NA | NR | NA | Y | NR | Y | N | Y | NA |
| (Elizabeth, Lauren et al. 2023) | Y | Y | NA | NA | Y | Y | Y | NA | Y | - | - | - | - | - |
| NCT04213469 (Hu, Zu et al. 2023) | N | NA | NA | N | N | NA | NR | NA | Y | N | Y | N | Y | NA |
| NCT04150497 (Jain, Chevallier et al. 2023) | N | NA | NA | N | N | NA | Y | NA | Y | Y | Y | N | Y | NA |
| NCT03919240 (Kang, Ma et al. 2023) | N | NA | NA | N | N | Y | NR | NR | Y | N | Y | N | Y | NA |
| NCT05454241 (Liu, Guo et al. 2023) | N | NA | NA | N | N | N | Y | NA | Y | Y | Y | N | Y | NA |
| NCT03229876 (Chen, Tan et al. 2024) | N | NA | NA | N | N | N | Y | NA | Y | N | Y | N | Y | NA |
| NCT04637763 (Hu, Nastoupil et al. 2024) | N | NA | NA | N | N | N | Y | NA | Y | N | Y | N | Y | NA |
| (Li, Xia et al. 2024) | Y | Y | NA | NA | Y | Y | Y | NA | Y | - | - | - | - | - |
| NCT05812326 (Lin, Yin et al. 2024) | N | NA | NA | N | N | NA | Y | NA | Y | N | Y | N | Y | NA |
| NCT06014073 (Liu, Shi et al. 2024) | N | NA | NA | N | N | NA | Y | NA | Y | Y | Y | N | Y | NA |
| (Ouyang, Jin et al. 2024) | N | NA | NA | N | N | NA | N | NA | Y | N | Y | N | Y | NA |
| NCT04438083 (Pal, Tran et al. 2024) | N | NA | NA | N | N | N | N | N | Y | N | Y | N | Y | NA |
| NCT05741359 (Zheng, Hu et al. 2024) | N | NA | NA | N | N | NR | NR | NR | Y | N | Y | N | Y | NA |
| NCT04502446 (Iyer, Sica et al. 2025) | N | NA | NA | N | N | NR | NR | NA | Y | N | Y | N | Y | NA |
| NCT04613557 (Lonez, Bolsée et al. 2025) | N | NA | NA | N | N | NA | NR | NA | Y | NR | Y | N | Y | NA |
| NCT05032599 (Pan, Tan et al. 2025) | N | NA | NA | N | N | NA | Y | NA | Y | N | Y | N | Y | NA |
| NCT04825496 (Xue, Tang et al. 2025) | N | NA | NA | N | N | NR | Y | Y | Y | N | Y | N | Y | NA |

**References**

"<https://www.clinicaltrials.gov/study/NCT04035434>, Last update: January 8th, 2025, Accessed online: August 11th, 2025.".

"Study Quality Assessment Tools, National Heart, Lung, and Blood Institute (NHLBI), Accessed at: February 18, 2024. Available online at: <https://www.nhlbi.nih.gov/health-topics/study-quality-assessment-tools>."

Adachi, Y., S. Terakura, M. Osaki, Y. Okuno, Y. Sato, K. Sagou, Y. Takeuchi, H. Yokota, K. Imai, P. Steinberger, J. Leitner, R. Hanajiri, M. Murata and H. Kiyoi (2024). "Cullin-5 deficiency promotes chimeric antigen receptor T cell effector functions potentially via the modulation of JAK/STAT signaling pathway." Nat Commun **15**(1): 10376.

Adeshakin, A. O., P. Zhou, J. Y. Métais, P. Nguyen, S. Perry, H. Sheppard, X. Sun, T. Cunningham, H. Shi, J. Wagner, J. T. Yustein, C. DeRenzo, G. Krenciute, H. Chi and S. Gottschalk (2023). "B7-H3-CAR T-cell therapy in immunecompetent osteosarcoma models: Regnase-1 KO overcomes limited CAR T-cell expansion." Cancer Research **83**(7).

Agarwal, S. (2020). "Investigating the Therapeutic Efficacy of Disruption of Cell Intrinsic Checkpoint Regulator CTLA-4 in Chimeric Antigen Receptor T Cells." Molecular Therapy **28**(4): 500-501.

Agarwal, S., M. A. Aznar, A. J. Rech, C. R. Good, S. Kuramitsu, T. Da, M. Gohil, L. Chen, S. A. Hong, P. Ravikumar, A. K. Rennels, J. Salas-Mckee, W. Kong, M. Ruella, M. M. Davis, G. Plesa, J. A. Fraietta, D. L. Porter, R. M. Young and C. H. June (2023). "Deletion of the inhibitory co-receptor CTLA-4 enhances and invigorates chimeric antigen receptor T cells." Immunity **56**(10): 2388-2407.e2389.

Ajmal, I., M. A. Farooq, Y. Duan, J. Yao, Y. Gao, X. Hui, Y. Ge, Y. Chen, Y. Ren, B. Du and W. Jiang (2024). "Intrinsic ADRB2 inhibition improves CAR-T cell therapy efficacy against prostate cancer." Mol Ther **32**(10): 3539-3557.

Alizadeh, D., R. Nee, Z. Wang, M. Maker, F. Chen, J. Hibbard and C. Brown (2023). "TARGETING TGFβ PATHWAY TO ENHANCE CAR-T THERAPY FOR GLIOBLASTOMA." Journal for ImmunoTherapy of Cancer **11**: A259.

An, N., Y. Pan, L. Yang, Q. Zhang, S. Deng, Q. Zhang and X. Du (2023). "Anti-Acute Myeloid Leukemia Activity of CD38-CAR-T Cells with PI3Kδ Downregulation." Mol Pharm **20**(5): 2426-2435.

Andreu-Saumell, I., A. Rodriguez-Garcia, V. Mühlgrabner, M. Gimenez-Alejandre, B. Marzal, J. Castellsagué, F. Brasó-Maristany, H. Calderon, L. Angelats, S. Colell, M. Nuding, M. Soria-Castellano, P. Barbao, A. Prat, A. Urbano-Ispizua, J. B. Huppa and S. Guedan (2024). "CAR affinity modulates the sensitivity of CAR-T cells to PD-1/PD-L1-mediated inhibition." Nat Commun **15**(1): 3552.

Angelos, M. G., R. P. Patel, H. Chiang, W. Xie, R. Pajarillo, C. E. Shaw, E. Singh, J. Xu, A. D. Posey, A. Rook, A. Rotolo, V. Pillai, S. A. Pileri, D. T. Teachey and M. Ruella (2023). "Fratricide-Resistant Anti-CD2 Chimeric Antigen Receptor T-Cells with Endogenous CD2 Knockout Are Highly Effective Against T-Cell Neoplasms." Blood **142**.

Bachl, S., A. Talbot, C. Ching, N. Kale, Z. Li, M. Diolaiti, A. P. Chen, J. Muldoon, C. Liu, T. Martin, J. Eyquem and J. Carnevale (2023). "Ablation of Cullin-5 in Primary Human T Cells Improves Tumor Killing and Persistence in BCMA-Targeting CAR-T Cells in a Multiple Myeloma Model." Blood **142**: 99.

Bailey, S. R., H. N. Takei, G. Escobar, M. C. Kann, A. A. Bouffard, T. Kienka, V. M. Supper, A. Armstrong, D. Salas-Benito, M. K. Phillips, F. Birocchi, S. Vatsa, H. Silva, I. Scarfò, M. Wehrli, K. Grauwet, E. P. Darnell, C. E. Graham, M. B. Leick, F. Korell, T. R. Berger and M. V. Maus (2025). "IFN-γ-resistant CD28 CAR T cells demonstrate increased survival, efficacy, and durability in multiple murine tumor models." Sci Transl Med **17**(801): eadp8166.

Bailey, S. R., S. Vatsa, R. C. Larson, A. A. Bouffard, I. Scarfò, M. C. Kann, T. R. Berger, M. B. Leick, M. Wehrli, A. Schmidts, H. Silva, K. A. Lindell, A. Demato, K. M. E. Gallagher, M. J. Frigault and M. V. Maus (2022). "Blockade or Deletion of IFNγ Reduces Macrophage Activation without Compromising CAR T-cell Function in Hematologic Malignancies." Blood Cancer Discov **3**(2): 136-153.

Balakrishnan, I., L. Leach, A. Pierce, S. Lakshmanachetty, K. Madhavan, S. Fosmire, H. Chatwin, A. Green, T. Fry, R. Vibhakar, E. M. Kohler and S. Venkataraman (2023). "PRECLINICAL TESTING OF THE EFFICACY AND SAFETY OF CRISPR/CAS9 GENE EDITED CAR-T CELL THERAPY FOR DIFFUSE MIDLINE GLIOMA (DMG)." Neuro-Oncology **25**: i20.

Baptista, G. and J. Carvalho (2023). "CAR‑T Cell Therapy With CRISPR/CAS9 Engineering for Relapsed/Refractory B‑Cell Acute Lymphoblastic Leukemia, With Universal Dual Target CD19/CD22." Clinical Lymphoma, Myeloma and Leukemia **23**: S529.

Battram, A. M., J. Mane-Pujol, D. F. Moreno, A. Oliver-Caldés, J. Carpio, O. Cardus, L. G. Rodríguez-Lobato, Á. Urbano-Ispizua and C. F. de Larrea (2025). "Genetic disruption of Blimp-1 drastically augments the antitumor efficacy of BCMA-targeting CAR T cells." Blood Advances **9**(3): 627-641.

Beavis, P. A., M. A. Henderson, L. Giuffrida, J. K. Mills, K. Sek, R. S. Cross, A. J. Davenport, L. B. John, S. Mardiana, C. Y. Slaney, R. W. Johnstone, J. A. Trapani, J. Stagg, S. Loi, L. Kats, D. Gyorki, M. H. Kershaw and P. K. Darcy (2017). "Targeting the adenosine 2A receptor enhances chimeric antigen receptor T cell efficacy." J Clin Invest **127**(3): 929-941.

Beckett, A. N., P. Chockley, S. M. Pruett-Miller, P. Nguyen, P. Vogel, H. Sheppard, G. Krenciute, S. Gottschalk and C. DeRenzo (2023). "CD47 expression is critical for CAR T-cell survival in vivo." J Immunother Cancer **11**(3).

Benjamin, R., C. Graham, D. Yallop, A. Jozwik, O. C. Mirci-Danicar, G. Lucchini, D. Pinner, N. Jain, H. Kantarjian, N. Boissel, M. V. Maus, M. J. Frigault, A. Baruchel, M. Mohty, A. Gianella-Borradori, F. Binlich, S. Balandraud, F. Vitry, E. Thomas, A. Philippe, S. Fouliard, S. Dupouy, I. Marchiq, M. Almena-Carrasco, N. Ferry, S. Arnould, C. Konto, P. Veys and W. Qasim (2020). "Genome-edited, donor-derived allogeneic anti-CD19 chimeric antigen receptor T cells in paediatric and adult B-cell acute lymphoblastic leukaemia: results of two phase 1 studies." Lancet **396**(10266): 1885-1894.

Benjamin, R., N. Jain, M. V. Maus, N. Boissel, C. Graham, A. Jozwik, D. Yallop, M. Konopleva, M. J. Frigault, T. Teshima, K. Kato, F. Boucaud, S. Balandraud, A. Gianella-Borradori, F. Binlich, I. Marchiq, S. Dupouy, M. Almena-Carrasco, M. Pannaux, S. Fouliard, E. Brissot and M. Mohty (2022). "UCART19, a first-in-class allogeneic anti-CD19 chimeric antigen receptor T-cell therapy for adults with relapsed or refractory B-cell acute lymphoblastic leukaemia (CALM): a phase 1, dose-escalation trial." Lancet Haematol **9**(11): e833-e843.

Benton, A., J. Liu, M. A. Poussin, A. Lang Goldgewicht, M. Udawela, A. S. Bear, N. Wellhausen, B. M. Carreno, P. M. Smith, M. D. Beasley, B. R. Kiefel and D. J. Powell, Jr. (2025). "Mutant KRAS peptide targeted CAR-T cells engineered for cancer therapy." Cancer Cell **43**(7): 1365-1376.e1365.

Bolaños, C. C., C. Calviño, A. A. Pierola, P. Jauregui, M. E. Calleja-Cervantes, P. San-Martin, P. R. Márquez, A. M. Mallo, E. Iglesias, G. Abizanda, S. R. Diaz, R. M. Turrillas, J. I. Esteban, A. M. Redondo, M. C. Viguria-Alegría, C. Panizo, J. J. Rifon, S. Villar, J. J. Lasarte, S. Inogés, A. L. D. De Cerio, M. Hernaez, F. Prosper and J. R. Rodriguez-Madoz (2023). "Optimization of a Universal Allogeneic CAR-T Cells Combining CRISPR and TransposonBased Technologies for Treatment of Acute Myeloid Leukemia." Blood **142**.

Braun, T., A. Pruene, M. Darguzyte, A. F. Vom Stein, P. H. Nguyen, D. L. Wagner, J. Kath, A. Roig-Merino, M. Heuser, L. L. Riehm, A. Schneider, S. Awerkiew, S. R. Talbot, A. Bleich, C. Figueiredo, M. Bornhäuser and R. Stripecke (2023). "Non-viral TRAC-knocked-in CD19(KI)CAR-T and gp350(KI)CAR-T cells tested against Burkitt lymphomas with type 1 or 2 EBV infection: In vivo cellular dynamics and potency." Front Immunol **14**: 1086433.

Bridge, J., M. J. Johnson, J. Kim, S. Wenthe, J. Krueger, B. Wick, M. Kluesner, A. T. Crane, J. Bell, J. G. Skeate, B. S. Moriarity and B. R. Webber (2024). "Efficient multiplex non-viral engineering and expansion of polyclonal γδ CAR-T cells for immunotherapy." bioRxiv.

Brown, C. E., A. Rodriguez, J. Palmer, J. R. Ostberg, A. Naranjo, J. Wagner, B. Aguilar, R. Starr, L. Weng, T. W. Synold, V. Tran, S. Wang, A. Reik, M. D'Apuzzo, J. A. Ressler, Y. Zhou, M. Mendel, P. D. Gregory, M. C. Holmes, W. W. Tang, S. J. Forman, M. C. Jensen and B. Badie (2022). "Off-the-shelf, Steroid Resistant, IL13Rα2-Specific CAR T Cells for Treatment of Glioblastoma." Neuro Oncol.

Cai, T., A. Gouble, K. L. Black, A. Skwarska, A. S. Naqvi, D. Taylor, M. Zhao, Q. Yuan, M. Sugita, Q. Zhang, R. Galetto, S. Filipe, A. Cavazos, L. Han, V. Kuruvilla, H. Ma, C. Weng, C. G. Liu, X. Liu, S. Konoplev, J. Gu, G. Tang, X. Su, G. Al-Atrash, S. Ciurea, S. S. Neelapu, A. A. Lane, H. Kantarjian, M. L. Guzman, N. Pemmaraju, J. Smith, A. Thomas-Tikhonenko and M. Konopleva (2022). "Targeting CD123 in blastic plasmacytoid dendritic cell neoplasm using allogeneic anti-CD123 CAR T cells." Nat Commun **13**(1): 2228.

Calderon, F. A., R. A. Fleming, L. Gorfinkel, K. Michaelis, X. L. Rui, J. Kaminski, D. Bonin, A. Mallette, K. Brodeur, H. Domingues, J. Lane, V. Tkachev, L. Kean and U. Gerdemann (2023). "Enhanced T Cell Function of PTPN2 Deleted CAR-T Cells Comes at a Cost: PTPN2 Knockout CAR-Ts Secrete More Cytokines and Demonstrate Increased Cytotoxicity, but Exhibit More Severe CRS and Icans in a Non-Human Primate Model." Blood **142**.

Calviño, C., C. Ceballos, A. Alfonso, P. Jauregui, M. E. Calleja-Cervantes, P. San Martin-Uriz, P. Rodriguez-Marquez, A. Martin-Mallo, E. Iglesias, G. Abizanda, S. Rodriguez-Diaz, R. Martinez-Turrillas, J. Illarramendi, M. C. Viguria, M. Redondo, J. Rifon, S. Villar, J. J. Lasarte, S. Inoges, A. Lopez-Diaz de Cerio, M. Hernaez, F. Prosper and J. R. Rodriguez-Madoz (2023). "Optimization of universal allogeneic CAR-T cells combining CRISPR and transposon-based technologies for treatment of acute myeloid leukemia." Front Immunol **14**: 1270843.

Cappabianca, D., D. Pham, M. H. Forsberg, M. Bugel, A. Tommasi, A. Lauer, J. Vidugiriene, B. Hrdlicka, A. McHale, Q. Sodji, M. C. Skala, C. M. Capitini and K. Saha (2024). "Metabolic priming of GD2 TRAC -CAR T cells during manufacturing promotes memory phenotypes while enhancing persistence." bioRxiv.

Carnevale, J., E. Shifrut, N. Kale, W. A. Nyberg, F. Blaeschke, Y. Y. Chen, Z. Li, S. P. Bapat, M. E. Diolaiti, P. O’Leary, S. Vedova, J. Belk, B. Daniel, T. L. Roth, S. Bachl, A. A. Anido, B. Prinzing, J. Ibañez-Vega, S. Lange, D. Haydar, M. Luetke-Eversloh, M. Born-Bony, B. Hegde, S. Kogan, T. Feuchtinger, H. Okada, A. T. Satpathy, K. Shannon, S. Gottschalk, J. Eyquem, G. Krenciute, A. Ashworth and A. Marson (2022). "RASA2 ablation in T cells boosts antigen sensitivity and long-term function." Nature **609**(7925): 174-182.

Cen, H., Q. Ke, Z. Li, D. Zhou, M. Wang, J. Sun and C. Liao (2022). "shRNA-mediated PD1 gene knock-down anti-CD19 CAR-T cell therapy for relapsed/refractory b cell malignancies." Annals of Oncology **33**(7): S827-S828.

Certo, M., S. Kurtulus, J. Evans, M. L. Cabral, N. Rouillard, V. K. Nguyen, M. Fujiwara, S. Adams, J. B. Rottman, D. Zak, E. Thompson, M. R. Perkins and P. D. Gregory (2023). "bbT369, a Clinical-Stage Dual-Targeted and CBLB Gene Edited Autologous CAR T Product for Non-Hodgkin Lymphoma, Shows Edit Driven Enhanced Activity in Preclinical In Vitro and In Vivo Models." Molecular Therapy **31**(4): 293-294.

Chan, T., C. Bolinger, S. Scott, M. Du, C. Poortman, B. Koenitzer, T. Athwal, L. Shepard, R. D. Slone, S. Dutta, S. Zilko, J. M. Dunleavey, G. Zenere, J. Plummer, B. Klocke, C. Zinser, S. Ahmad, D. E. Brough, R. R. Shah and H. Sabzevari (2022). "Incorporation of intrinsic checkpoint blockade enhances functionality of multigenic autologous UltraCAR-T® cells manufactured using non-viral gene delivery and rapid manufacturing process." Cancer Research **82**(12).

Chang, C., E. Peralta, G. Hsia, B. H. Yang, W. I. Yeh, R. Clarke, M. Mili, T. Lee, M. Pribadi, R. Abujarour, Y. S. Lai, D. Robbins, M. Denholtz, S. K. Hanok, E. Driver, E. Carron, N. Navarrete, M. Sumi, A. Mehta, P. Chu, J. Orourke, A. Gutierrez, E. Sung, S. Gasparian, A. Gentile, A. Witty, A. Yzaguirre, S. LaBarge, C. Nguyen and B. Valamehr (2020). "Generation of Multiplexed Engineered, Off-the-Shelf CAR T Cells Uniformly Carrying Multiple Anti-Tumor Modalities to Prevent Tumor Relapse." Blood **136**: 11.

Chang, C., S. Van Der Stegen, M. Mili, R. Clarke, Y. S. Lai, A. Witty, P. Lindenbergh, B. H. Yang, M. Husain, H. Shaked, B. Groff, L. Stokely, R. Abujarour, T. Lee, H. Y. Chu, M. Pribadi, J. Orourke, A. Gutierrez, I. Riviere, M. Sadelain and B. Valamehr (2019). "FT819: Translation of Off-the-Shelf TCR-Less Trac-1XX CAR-T Cells in Support of First-of-Kind Phase I Clinical Trial." Blood **134**: 4434.

Chen, A. X. Y., K. M. Yap, J. S. Kim, K. Sek, Y. K. Huang, P. A. Dunbar, V. Wiebking, J. D. Armitage, I. Munoz, K. L. Todd, E. B. Derrick, D. Nguyen, J. Tong, C. W. Chan, T. X. Hoang, K. M. Audsley, M. J. van Elsas, J. Middelburg, J. N. Lee, M. N. de Menezes, T. J. Cole, J. Li, C. Scheffler, A. M. Scott, L. K. Mackay, J. Waithman, J. Oliaro, S. J. Harrison, I. A. Parish, J. Lai, M. H. Porteus, I. G. House, P. K. Darcy and P. A. Beavis (2025). "Rewiring endogenous genes in CAR T cells for tumour-restricted payload delivery." Nature.

Chen, J., I. F. López-Moyado, H. Seo, C. J. Lio, L. J. Hempleman, T. Sekiya, A. Yoshimura, J. P. Scott-Browne and A. Rao (2019). "NR4A transcription factors limit CAR T cell function in solid tumours." Nature **567**(7749): 530-534.

Chen, L. Y., L. Q. Kang, H. X. Zhou, H. Q. Gao, X. F. Zhu, N. Xu, L. Yu, D. P. Wu, S. L. Xue and A. N. Sun (2020). "Successful application of anti-CD19 CAR-T therapy with IL-6 knocking down to patients with central nervous system B-cell acute lymphocytic leukemia." Transl Oncol **13**(11): 100838.

Chen, S., Y. Lin, S. Zhong, H. An, Y. Lu, M. Yin, W. Liang and E. M. McGowan (2018). "Anti-MUC1 CAR-T cells combined with PD-1 knockout engineered T cells for patients with non-small cell lung cancer (NSCLC): A pilot study." Annals of Oncology **29**.

Chen, S. Z. and Y. G. Lin (2023). "Phase I clinical trial using a unique immunotherapeutic combination of MUC1-targeted CAR-T cells with PD-1-knockout in the treatment of patients with advanced esophageal cancer." Journal of Clinical Oncology **41**(16).

Chen, T., J. Deng, Y. Zhang, B. Liu, R. Liu, Y. Zhu, M. Zhou, Y. Lin, B. Xia, K. Lin, X. Ma and H. Zhang (2024). "The construction of modular universal chimeric antigen receptor T (MU-CAR-T) cells by covalent linkage of allogeneic T cells and various antibody fragments." Mol Cancer **23**(1): 53.

Chen, X., B. Tan, H. Xing, X. Zhao, Y. Ping, Z. Zhang, J. Huang, X. Shi, N. Zhang, B. Lin, W. Cao, X. Li, X. Zhang, L. Li, Z. Jiang, M. Zhang, W. Li, M. Liu, B. Du and Y. Zhang (2024). "Allogeneic CAR-T cells with of HLA-A/B and TRAC disruption exhibit promising antitumor capacity against B cell malignancies." Cancer Immunol Immunother **73**(1): 13.

Cheng, Y., J. Zhang, W. Mu, S. Ye, J. Cheng, L. Zhu, G. Wang, Y. Cao, D. Li, G. Hu, L. Huang, J. Wang and J. Zhou (2025). "Dasatinib-resistant universal CAR-T cells proliferate in the presence of host immune cells and exhibit antitumor activity." Mol Ther **33**(4): 1535-1551.

Cherkassky, L., A. Morello, J. Villena-Vargas, Y. Feng, D. S. Dimitrov, D. R. Jones, M. Sadelain and P. S. Adusumilli (2016). "Human CAR T cells with cell-intrinsic PD-1 checkpoint blockade resist tumor-mediated inhibition." J Clin Invest **126**(8): 3130-3144.

Chiesa, R., C. Georgiadis, F. Syed, H. Zhan, A. Etuk, S. A. Gkazi, R. Preece, G. Ottaviano, T. Braybrook, J. Chu, A. Kubat, S. Adams, R. Thomas, K. Gilmour, D. O'Connor, A. Vora and W. Qasim (2023). "Base-Edited CAR7 T Cells for Relapsed T-Cell Acute Lymphoblastic Leukemia." N Engl J Med **389**(10): 899-910.

Choi, B., X. L. Yu, A. Castano, A. Bouffard, M. Frigault, A. Schmidts, W. Curry, B. Carter and M. Maus (2020). "Intraventricular Delivery and CRISPR-Cas9 Disruption of PD-1 is required for CAR T-cell Efficacy in Glioblastoma." Journal of Neurosurgery **132**(4): 7-7.

Choi, B. D., X. Yu, A. P. Castano, H. Darr, D. B. Henderson, A. A. Bouffard, R. C. Larson, I. Scarfò, S. R. Bailey, G. M. Gerhard, M. J. Frigault, M. B. Leick, A. Schmidts, J. G. Sagert, W. T. Curry, B. S. Carter and M. V. Maus (2019). "CRISPR-Cas9 disruption of PD-1 enhances activity of universal EGFRvIII CAR T cells in a preclinical model of human glioblastoma." J Immunother Cancer **7**(1): 304.

Chu, N. J., C. Nava-Barbero, A. Merlino, P. H. Lee, D. Baker, B. Taylor, G. Moody and L. Giardino (2024). "CRISPR-mediated knock-out of A20 lowers the activation threshold and induces metabolic rewiring of CAR-T cells." Cancer Research **84**(6).

Chun, I., K. H. Kim, Y. H. Chiang, W. Xie, Y. G. G. Lee, R. Pajarillo, A. Rotolo, O. Shestova, S. J. Hong, M. Abdel-Mohsen, M. Wysocka, H. J. Ballard, D. M. Barrett, A. D. Posey, D. Powell, S. I. Gill, S. J. Schuster, S. K. Barta, A. H. Rook, C. H. June and M. Ruella (2020). "CRISPR-Cas9 Knock out of CD5 Enhances the Anti-Tumor Activity of Chimeric Antigen Receptor T Cells." Blood **136**.

Condomines, M., J. Arnason, R. Benjamin, G. Gunset, J. Plotkin and M. Sadelain (2015). "Tumor-Targeted Human T Cells Expressing CD28-Based Chimeric Antigen Receptors Circumvent CTLA-4 Inhibition." PLoS One **10**(6): e0130518.

Cooper, M. L., J. Choi, K. Staser, J. K. Ritchey, J. M. Devenport, K. Eckardt, M. P. Rettig, B. Wang, L. G. Eissenberg, A. Ghobadi, L. N. Gehrs, J. L. Prior, S. Achilefu, C. A. Miller, C. C. Fronick, J. O’Neal, F. Gao, D. M. Weinstock, A. Gutierrez, R. S. Fulton and J. F. DiPersio (2018). "An “off-the-shelf” fratricide-resistant CAR-T for the treatment of T cell hematologic malignancies." Leukemia **32**(9): 1970-1983.

Cooper, M. L., K. W. Staser, J. Ritchey, J. Niswonger, B. H. Lee, J. Park and J. F. DiPersio (2018). "A long-acting pharmacological grade interleukin-7 molecule logarithmically accelerates ucart proliferation, differentiation, and tumor killing." Blood **132**.

Cox, M. J., C. Manriquez Roman, E. E. Tapper, E. L. Siegler, D. Chappell, C. Durrant, O. Ahmed, S. Sinha, R. Mwangi, N. S. Scott, M. Hefazi, K. J. Schick, P. Horvei, M. W. Ruff, I. Can, M. Adada, E. Bezerra, L. A. Kankeu Fonkoua, S. A. Parikh, N. E. Kay, R. Sakemura and S. S. Kenderian (2022). "GM-CSF disruption in CART cells modulates T cell activation and enhances CART cell anti-tumor activity." Leukemia **36**(6): 1635-1645.

Dai, Z., W. Mu, Y. Zhao, J. Cheng, H. Lin, K. Ouyang, X. Jia, J. Liu, Q. Wei, M. Wang, C. Liu, T. Tan and J. Zhou (2022). "T cells expressing CD5/CD7 bispecific chimeric antigen receptors with fully human heavy-chain-only domains mitigate tumor antigen escape." Signal Transduct Target Ther **7**(1): 85.

Dai, Z., W. Mu, Y. Zhao, X. Jia, J. Liu, Q. Wei, T. Tan and J. Zhou (2021). "The rational development of CD5-targeting biepitopic CARs with fully human heavy-chain-only antigen recognition domains." Mol Ther **29**(9): 2707-2722.

Das, S., J. Valton, P. Duchateau and L. Poirot (2023). "Stromal depletion by TALEN-edited universal hypoimmunogenic FAP-CAR T cells enables infiltration and anti-tumor cytotoxicity of tumor antigen-targeted CAR-T immunotherapy." Front Immunol **14**: 1172681.

David, M., P. Schiele, D. Monteferrario, G. Saviane, A. E. Martelli, C. F. Dupont, C. Jeanneau, I. Marchetti, S. K. Tadi, J. Vahldick, L. N. Truong, Y. Y. Zhou, I. M. Sauer, W. Schöning, I. K. Na, A. Reik, M. Frentsch, M. de la Rosa and D. Fenard (2025). "Enhanced anti-tumor activity by zinc finger repressor-driven epigenetic silencing of immune checkpoints and TGFBR2 in CAR-T cells and TILs." Molecular Therapy Oncology **33**(2).

Dawn Sakthi Vale, P., L. Wu, V. J. Y. Tan, B. Chua, L. Wu, J. C. Tan, C. K. T. Koh, J. Ong and N. Gascoigne (2022). "LCK KNOCKOUT CAR-T CELLS AS A NOVEL ALLOGENEIC PLATFORM." Journal for ImmunoTherapy of Cancer **10**: A289.

De Munter, S., J. L. Buhl, L. De Cock, A. Van Parys, W. Daneels, E. Pascal, L. Deseins, J. Ingels, G. Goetgeluk, H. Jansen, L. Billiet, M. Pille, J. Van Duyse, S. Bonte, N. Vandamme, J. Van Dorpe, F. Offner, G. Leclercq, T. Taghon, E. Depla, J. Tavernier, T. Kerre, J. Drost and B. Vandekerckhove (2024). "Knocking Out CD70 Rescues CD70-Specific NanoCAR T Cells from Antigen-Induced Exhaustion." Cancer Immunol Res **12**(9): 1236-1251.

Deeren, D., J. A. Maertens, T. Lin, Y. Beguin, B. Demoulin, M. Fontaine, P. A. Sotiropoulou, E. Alcantar-Orozco, E. Breman, M. S. Dheur, N. Braun, C. Lonez, D. E. Gilham, A. Flament and F. F. Lehmann (2020). "First Results from the Dose Escalation Segment of the Phase I Clinical Study Evaluating Cyad-02, an Optimized Non Gene-Edited Engineered NKG2D CAR T-Cell Product, in Relapsed or Refractory Acute Myeloid Leukemia and Myelodysplastic Syndrome Patients." Blood **136**.

Degagné, É., P. D. Donohoue, S. Roy, J. Scherer, T. W. Fowler, R. T. Davis, G. A. Reyes, G. Kwong, M. Stanaway, V. Larroca Vicena, D. Mutha, R. Guo, L. Edwards, B. Schilling, M. Shaw, S. C. Smith, B. Kohrs, H. J. Kufeldt, G. Churchward, F. Ruan, D. B. Nyer, K. McSweeney, M. J. Irby, C. K. Fuller, L. Banh, M. S. Toh, M. Thompson, A. L. G. Owen, Z. An, S. Gradia, J. Skoble, M. Bryan, E. Garner and S. B. Kanner (2024). "High-Specificity CRISPR-Mediated Genome Engineering in Anti-BCMA Allogeneic CAR T Cells Suppresses Allograft Rejection in Preclinical Models." Cancer Immunol Res **12**(4): 462-477.

Dequeant, M. L., J. Sagert, D. Kalaitzidis, H. Yu, A. Porras, B. McEwan, Z. Padalia, P. Tetteh, T. Nguyen, A. Dunn, S. Spencer, N. Lee, H. Dar, D. Henderson, S. Karnik, P. Keerthipati and J. A. Terrett (2021). "CD70 knockout: A novel approach to augment CAR-T cell function." Cancer Research **81**(13).

Dharani, S., H. Cho, J. P. Fernandez, A. Juillerat, J. Valton, P. Duchateau, L. Poirot and S. Das (2024). "TALEN-edited allogeneic inducible dual CAR T cells enable effective targeting of solid tumors while mitigating off-tumor toxicity." Mol Ther **32**(11): 3915-3931.

Dholaria, B., M. H. Kocoglu, A. Kin, A. S. Asch, A. Ramakrishnan, C. Bachier, T. E. Rodriguez, L. Shune, K. McArthur, J. McCaigue, S. DePrimo, C. Martin, S. Haag, M. Zhang, H. Namini, E. Christie, R. Belani, S. A. Cranert, J. Coronella, D. J. Shedlock and C. L. Costello (2023). "Early Safety Results of P-BCMA-ALLO1, a Fully Allogeneic Chimeric Antigen Receptor T-Cell (CAR-T), in Patients with Relapsed / Refractory Multiple Myeloma (RRMM)." Blood **142**: 3479.

Diorio, C., R. Murray, M. Naniong, L. Barrera, A. Camblin, J. Chukinas, L. Coholan, A. Edwards, T. Fuller, C. Gonzales, S. A. Grupp, A. Ladd, M. Le, A. Messana, F. Musenge, H. Newman, Y. C. Poh, H. Poulin, T. Ryan, R. Shraim, S. K. Tasian, T. Vincent, L. Young, Y. Zhang, G. Ciaramella, J. Gehrke and D. T. Teachey (2022). "Cytosine base editing enables quadruple-edited allogeneic CART cells for T-ALL." Blood **140**(6): 619-629.

Doan, A. E., K. P. Mueller, A. Y. Chen, G. T. Rouin, Y. Chen, B. Daniel, J. Lattin, M. Markovska, B. Mozarsky, J. Arias-Umana, R. Hapke, I. Y. Jung, A. Wang, P. Xu, D. Klysz, G. Zuern, M. Bashti, P. J. Quinn, Z. Miao, K. Sandor, W. Zhang, G. M. Chen, F. Ryu, M. Logun, J. Hall, K. Tan, S. A. Grupp, S. E. McClory, C. A. Lareau, J. A. Fraietta, E. Sotillo, A. T. Satpathy, C. L. Mackall and E. W. Weber (2024). "FOXO1 is a master regulator of memory programming in CAR T cells." Nature **629**(8010): 211-218.

Dörr, J., L. Gregor, S. B. Lacher, A. Öner, S. Lesch, S. Michaelides, L. Majed, L. Fertig, E. Carlini, D. Andreu Sanz, D. Briukhovetska, S. Stock, A. Gottschlich, J. P. Böttcher and S. Kobold (2024). "PROSTANOID-INSENSITIVE CHIMERIC ANTIGEN RECEPTOR MODIFIED T CELLS MEDIATE THERAPEUTIC EFFICACY IN SOLID CANCER MODELS." Journal for ImmunoTherapy of Cancer **12**: A4.

Dötsch, S., M. Svec, K. Schober, M. Hammel, A. Wanisch, F. Gökmen, S. Jarosch, L. Warmuth, J. Barton, L. Cicin-Sain, E. D'Ippolito and D. H. Busch (2023). "Long-term persistence and functionality of adoptively transferred antigen-specific T cells with genetically ablated PD-1 expression." Proc Natl Acad Sci U S A **120**(10): e2200626120.

Drake, C., A. Garcia, C. W. Chang, B. H. Yang, S. Ibitokou, C. Pride, S. Markov, A. Liao, M. Pribadi, Y. Pan, T. Dailey, T. Lee, S. R. Chan, M. Ports, J. Goodridge, R. Bjordahl, J. Erhardt and B. Valamehr (2022). "OFF-THE-SHELF IPSC-DERIVED CAR-T CELLS TARGETING KLK2 DEMONSTRATE PROLONGED TUMOR CONTROL AND SURVIVAL IN XENOGRAFT MODELS OF PROSTATE CANCER." Journal for ImmunoTherapy of Cancer **10**: A343.

Elizabeth, B., P. B. Lauren, K. Christina, M. Emiri, J. Blake, H. Ashley, M. Donna, C. Shally, B. Kalin, T. Ben, L. Guy, D. Franco, G. Ashraf, G. Elizabeth, Z. Enrique, K. Steven, N. Tonia, P. Socorro, R. Syed and O. Susan (2023). "Durable Complete Response achieved in Relapsed/ Refractory Diffuse Large B Cell Lymphoma (DLBCL) patient treated with a CRISPR-Edited Allogeneic Anti-CD19 CAR-T Cell Therapy with a PD-1 Knockout: Case Report from the CB-010 ANTLER Trial." American Journal of Hematology **98**: S37-S37.

Engel, N. W., I. Steinfeld, D. Ryan, K. Anupindi, S. Kim, N. Wellhausen, L. Chen, K. Wilkins, D. J. Baker, P. C. Rommel, D. Jarocha, M. Gohil, Q. Zhang, M. C. Milone, J. A. Fraietta, M. Davis, R. M. Young and C. H. June (2025). "Quadruple adenine base-edited allogeneic CAR T cells outperform CRISPR/Cas9 nuclease-engineered T cells." Proc Natl Acad Sci U S A **122**(20): e2427216122.

Evtimov, V. J., N. N. Nguyen, M. V. Hammett, A. Pupovac, P. J. Hudson, J. Zhuang, J. Y. Lee, S. Kim, A. O. Trounson, R. L. Boyd and R. Shu (2025). "CRISPR-Cas9 knockout of DGKα/ζ improves the anti-tumor activities of TAG-72 CAR-T cells in ovarian cancer." Mol Ther Oncol **33**(2): 200962.

Fraessle, S. P., C. Tschulik, M. Effenberger, V. Cletiu, M. Gerget, K. Schober, D. H. Busch, L. Germeroth, C. Stemberger and M. P. Poltorak (2023). "Activation-inducible CAR expression enables precise control over engineered CAR T cell function." Commun Biol **6**(1): 604.

Fraietta, J. A., C. L. Nobles, M. A. Sammons, S. Lundh, S. A. Carty, T. J. Reich, A. P. Cogdill, J. J. D. Morrissette, J. E. DeNizio, S. Reddy, Y. Hwang, M. Gohil, I. Kulikovskaya, F. Nazimuddin, M. Gupta, F. Chen, J. K. Everett, K. A. Alexander, E. Lin-Shiao, M. H. Gee, X. Liu, R. M. Young, D. Ambrose, Y. Wang, J. Xu, M. S. Jordan, K. T. Marcucci, B. L. Levine, K. C. Garcia, Y. Zhao, M. Kalos, D. L. Porter, R. M. Kohli, S. F. Lacey, S. L. Berger, F. D. Bushman, C. H. June and J. J. Melenhorst (2018). "Disruption of TET2 promotes the therapeutic efficacy of CD19-targeted T cells." Nature **558**(7709): 307-312.

Freitas, K. A., J. A. Belk, E. Sotillo, P. J. Quinn, M. C. Ramello, M. Malipatlolla, B. Daniel, K. Sandor, D. Klysz, J. Bjelajac, P. Xu, K. A. Burdsall, V. Tieu, V. T. Duong, M. G. Donovan, E. W. Weber, H. Y. Chang, R. G. Majzner, J. M. Espinosa, A. T. Satpathy and C. L. Mackall (2022). "Enhanced T cell effector activity by targeting the Mediator kinase module." Science **378**(6620): eabn5647.

Fu, Z., Z. Huang, H. Xu, Q. Liu, J. Li, K. Song, Y. Deng, Y. Tao, H. Zhang, P. Wang, H. Li, Y. Sheng, A. Zhou, L. Han, Y. Fu, C. Wang, S. K. Choudhary, K. Ye, G. Veggiani, Z. Li, A. August, W. Huang, Q. Shan and H. Peng (2024). "IL-2-inducible T cell kinase deficiency sustains chimeric antigen receptor T cell therapy against tumor cells." J Clin Invest **135**(4).

Gagnon, J., A. J. Litterman, J. A. Hall, D. Polyak, S. Zhou, S. Joshi, O. Takacsi-Nagy, H. Pope, L. Silva, B. K. Singh, J. M. Granja, D. DeTomaso, E. Aristillepe, M. Tan, B. Galvin, G. X. Zheng, S. Santoro, A. Cooper, N. Bezman and W. N. Haining (2022). "Multiplexed shRNAs targeting FAS and PTPN2 enhance CAR T persistence and anti-tumor efficacy." Cancer Research **82**(12).

Garner, E., E. Kelly, S. Namburi, C. Colgan, T. Fowler, D. Mutha, A. Aviles, M. Stanaway, R. Guo, Z. L. An, E. Degagne, G. Kwong, L. Edwards, E. Jakes, M. Shaw, B. Schilling, J. Huynh, R. Luu, M. Sidorov, R. Mousali, M. Otsmaa, J. Skoble and S. Kanner (2023). "CB-012, an allogeneic anti-CLL-1 CAR-T cell therapy engineered with next-generation CRISPR technology to resist both the immunosuppressive tumor microenvironment and immune cell-mediated rejection, for patients with relapsed or refractory acute myeloid leukemia." Cancer Research **83**(7).

Georgiadis, C., L. Nickolay, F. Syed, H. Zhan, S. A. Gkazi, A. Etuk, U. Abramowski-Mock, R. Preece, P. Cuber, S. Adams, G. Ottaviano and W. Qasim (2024). "Umbilical cord blood T cells can be isolated and enriched by CD62L selection for use in 'off the shelf' chimeric antigen receptor T-cell therapies to widen transplant options." Haematologica **109**(12): 3941-3951.

Georgiadis, C., R. Preece, L. Nickolay, A. Etuk, A. Petrova, D. Ladon, A. Danyi, N. Humphryes-Kirilov, A. Ajetunmobi, D. Kim, J. S. Kim and W. Qasim (2018). "Long Terminal Repeat CRISPR-CAR-Coupled "Universal" T Cells Mediate Potent Anti-leukemic Effects." Mol Ther **26**(5): 1215-1227.

Georgiadis, C., J. Rasaiyaah, S. A. Gkazi, R. Preece, A. Etuk, A. Christi and W. Qasim (2020). "Multiplexed Cytidine Deamination Enables Generation of Fratricide-Resistant ‘Universal T v T’ Chimeric Antigen Receptor Cell Therapy." Molecular Therapy **28**(4): 212-212.

Giuffrida, L., K. Sek, M. A. Henderson, J. Lai, A. X. Y. Chen, D. Meyran, K. L. Todd, E. V. Petley, S. Mardiana, C. Mølck, G. D. Stewart, B. J. Solomon, I. A. Parish, P. J. Neeson, S. J. Harrison, L. M. Kats, I. G. House, P. K. Darcy and P. A. Beavis (2021). "CRISPR/Cas9 mediated deletion of the adenosine A2A receptor enhances CAR T cell efficacy." Nat Commun **12**(1): 3236.

Goala, P., M. Davila, Y. Zhang, N. Betty, S. McSain, S. Cooper, M. J. Tariq and S. Hamid (2025). "Modulation of Th1/Th17 axis through IFNγ-blockade alleviates Anti-CD19 CAR-T associated toxicities in a pre-clinical immune competent model." Cancer Research **85**(8).

Gomes-Silva, D., E. Atilla, P. A. Atilla, F. Mo, H. Tashiro, M. Srinivasan, P. Lulla, R. H. Rouce, J. M. S. Cabral, C. A. Ramos, M. K. Brenner and M. Mamonkin (2019). "CD7 CAR T Cells for the Therapy of Acute Myeloid Leukemia." Mol Ther **27**(1): 272-280.

Gong, W. J., Y. Qiu, M. H. Li, L. Y. Chen, Y. Y. Li, J. Q. Yu, L. Q. Kang, A. N. Sun, D. P. Wu, L. Yu and S. L. Xue (2022). "Investigation of the risk factors to predict cytokine release syndrome in relapsed or refractory B-cell acute lymphoblastic leukemia patients receiving IL-6 knocking down anti-CD19 chimeric antigen receptor T-cell therapy." Front Immunol **13**: 922212.

Goto, A., M. Ishii, S. Kinoshita, Y. Furukawa, N. Izumi, J. Ando, H. Nakauchi and M. Ando (2024). iPSC-Derived Chimeric Antigen Receptor T Cells That Originally Lack CD5 Expression Exhibits Robust Cytotoxicity Against T-Cell Malignancies. **144:** 2029.

Gouble, A., R. Galetto, R. Mathur, S. Filipe, I. Chion-Sotinel, J. Yang, J. He, R. Orlowski, S. Neelapu and J. Smith (2018). "Universal CAR T-cells targeting CS1 (UCARTCS1) for the treatment of multiple myeloma." Molecular Therapy **26**(5): 63.

Gouble, A., B. Philip, L. Poirot, C. Schiffer-Mannioui, R. Galetto, S. Derniame, G. W. K. Cheung, S. Arnould, C. Desseaux, M. Pule and J. Smith (2014). "In vivo proof of concept of activity and safety of UCART19, an allogeneic “Off-the-Shelf” adoptive T-Cell immunotherapy against CD19+ B-Cell leukemias." Blood **124**(21).

Gough, O. J., C. Georgiadis, R. Preece and W. Qasim (2025). "Humanised comparison of 'universal' lentiviral-integrated and CRISPR-mediated, virus-free CAR T cells." Human Gene Therapy **36**(3-4): E453-E453.

Graham, C. E., A. Jozwik, R. Quartey-Papafio, N. Ioannou, A. M. Metelo, C. Scala, G. Dickson, O. Stewart, M. Almena-Carrasco, E. Peranzoni, A. G. Ramsay, P. E. M. Patten, T. Pertel, F. Farzaneh, S. Dupouy, A. Pepper and R. Benjamin (2021). "Gene-edited healthy donor CAR T cells show superior anti-tumour activity compared to CAR T cells derived from patients with lymphoma in an in vivo model of high-grade lymphoma." Leukemia **35**(12): 3581-3584.

Grauwet, K., T. Berger, M. C. Kann, H. Silva, R. Larson, M. B. Leick, S. R. Bailey, A. A. Bouffard, D. Millar, K. Gallagher, C. J. Turtle, M. J. Frigault and M. V. Maus (2024). "Stealth transgenes enable CAR-T cells to evade host immune responses." J Immunother Cancer **12**(5).

Gregor, L., J. Dörr, S. Lacher, A. Aner, S. Lesch, S. Michaelides, L. Majed, L. Fertig, E. Carlini, D. Andreu Sanz, D. Briukhovetska, S. Stock, A. Gottschlich, Y. Sun, R. W. Jenkins, I. Piseddu, J. P. Böttcher and S. Kobold (2025). "PROSTANOID-INSENSITIVE CHIMERIC ANTIGEN RECEPTOR MODIFIED T CELLS MEDIATE THERAPEUTIC EFFICACY IN SOLID CANCER MODELS." Journal for ImmunoTherapy of Cancer **13**: A13-A14.

Guo, X., H. Jiang, B. Shi, M. Zhou, H. Zhang, Z. Shi, G. Du, H. Luo, X. Wu, Y. Wang, R. Sun and Z. Li (2018). "Disruption of PD-1 Enhanced the Anti-tumor Activity of Chimeric Antigen Receptor T Cells Against Hepatocellular Carcinoma." Front Pharmacol **9**: 1118.

Guo, Y., C. Tong, L. Su, W. Zhang, H. Jia, Y. Liu, Q. Yang, Z. Wu, Y. Wang and W. Han (2022). "CRISPR/Cas9 genome-edited universal CAR T cells in patients with relapsed and refractory lymphoma." Blood Adv.

Guo, Y., C. Tong, L. Su, W. Zhang, H. Jia, Y. Liu, Q. Yang, Z. Wu, Y. Wang and W. Han (2022). "CRISPR/Cas9 genome-edited universal CAR T cells in patients with relapsed/refractory lymphoma." Blood Adv **6**(8): 2695-2699.

Guruprasad, P., A. Carturan, Y. Zhang, J. H. Cho, K. G. Kumashie, R. P. Patel, K. H. Kim, J. S. Lee, Y. Lee, J. H. Kim, J. Chung, A. Joshi, I. Cohen, M. Shestov, G. Ghilardi, J. Harris, R. Pajarillo, M. Angelos, Y. G. Lee, S. Liu, J. Rodriguez, M. Wang, H. J. Ballard, A. Gupta, O. H. Ugwuanyi, S. J. A. Hong, A. C. Bochi-Layec, C. T. Sauter, L. Chen, L. Paruzzo, S. Kammerman, O. Shestova, D. Liu, L. A. Vella, S. J. Schuster, J. Svoboda, P. Porazzi and M. Ruella (2024). "The BTLA-HVEM axis restricts CAR T cell efficacy in cancer." Nat Immunol **25**(6): 1020-1032.

Guzman, M. L., M. Sugita, H. Zong, N. Ewing-Crystal, V. Trujillo-Alonso, N. Mencia-Trinchant, L. Lam, N. M. Cruz, R. Galetto, A. Gouble, D. C. Hassane, J. Smith and G. J. Roboz (2016). "Allogeneic tcrα/β deficient CAR T-cells targeting CD123 prolong overall survival of AML patient-derived xenografts." Blood **128**(22).

Harcha, J. L., D. Hart, K. Chant, A. Hammad, F. Davi, S. Chung, M. Bryan, J. Skoble, E. Garner, T. Nesheiwat, S. Portella, S. Kanner, S. Rizvi and J. H. Essel (2022). "A CRISPR-edited Allogeneic Anti-CD19 CAR-T Cell Therapy with PD-1 Knockout Induces Prolonged Complete Response in Relapsed/Refractory Follicular Lymphoma Patient: Case Report from CB-010 ANTLER Study." American Journal of Hematology **97**: S4-S5.

Ho, L. Y., S. R. Yu, J. H. Jeong, H. J. Lee, H. J. Cho and H. C. Kim (2023). "Mitigating the CD5 CAR-CD5 interaction enhances the functionality of CD5 CAR-T cells by alleviating the T-cell fratricide." Cancer Research **83**(7).

Hooijmans, C. R., M. M. Rovers, R. B. M. de Vries, M. Leenaars, M. Ritskes-Hoitinga and M. W. Langendam (2014). "SYRCLE’s risk of bias tool for animal studies." BMC Medical Research Methodology **14**(1): 43.

Hooper, K., K. Havens, A. R. Krostag, M. S. Magee, U. Martin, A. Gupta, Y. Smurnyy, L. Pechilis, A. Rode, A. Chavkin, S. Grande, R. A. Morgan, J. Jarjour and A. Astrakhan (2018). "Knockout of CBLB Greatly Enhances Anti-Tumor Activity of CAR T Cells." Blood **132**.

Hu, B., L. J. Nastoupil, H. Holmes, A. Hamdan, A. Kanate, U. Farooq, M. Cherry, E. Brem, L. C. Pinter-Brown, D. A. Ermann, M. Husnain, K. Micklethwaite, S. Rizvi, A. Hammad, B. Thompson, E. Zudaire, S. Portella, M. Hamadani, J. Essell and S. M. O'Brien (2024). "A CRISPR-edited allogeneic anti-CD19 CAR-T cell therapy with a PD-1 knockout (CB-010) in patients with relapsed/refractory B cell non-Hodgkin lymphoma (r/r B-NHL): Updated phase 1 results from the ANTLER trial." Journal of Clinical Oncology **42**(16).

Hu, B., Y. Yi, X. Wang and G. Tao (2020). "Automatic Neutralization of IL6 Storm and Blockade of IL1 Signaling During CART Therapy to Reduce Cytokine Toxicity and Minimize Neurotoxicity." Molecular Therapy **28**(4): 213-214.

Hu, B., Y. Zou, L. Zhang, J. Tang, G. Niedermann, E. Firat, X. Huang and X. Zhu (2019). "Nucleofection with Plasmid DNA for CRISPR/Cas9-Mediated Inactivation of Programmed Cell Death Protein 1 in CD133-Specific CAR T Cells." Hum Gene Ther **30**(4): 446-458.

Hu, J. F., Z. W. Wang, C. Y. Liao, Z. W. Chen, F. P. Kang, C. F. Lin, T. S. Lin, L. Huang, Y. F. Tian and S. Chen (2022). "Induced expression of CCL19 promotes the anti-tumor ability of CAR-T cells by increasing their infiltration ability." Front Immunol **13**: 958960.

Hu, L., L. Chen, Z. Xiao, X. Zheng, Y. Chen, N. Xian, C. Cho, L. Luo, G. Huang and L. Chen (2022). "Ablation of T cell-associated PD-1H enhances functionality and promotes adoptive immunotherapy." JCI Insight **7**(2).

Hu, W., Z. Zi, Y. Jin, G. Li, K. Shao, Q. Cai, X. Ma and F. Wei (2019). "CRISPR/Cas9-mediated PD-1 disruption enhances human mesothelin-targeted CAR T cell effector functions." Cancer Immunol Immunother **68**(3): 365-377.

Hu, X., K. Manner, R. DeJesus, K. White, C. Gattis, P. Ngo, C. Bandoro, E. Tham, E. Y. Chu, C. Young, F. Wells, R. Basco, A. Friera, D. Kangeyan, P. Beauchesne, W. E. Dowdle, T. Deuse, T. J. Fry, A. E. Foster and S. Schrepfer (2023). "Hypoimmune anti-CD19 chimeric antigen receptor T cells provide lasting tumor control in fully immunocompetent allogeneic humanized mice." Nat Commun **14**(1): 2020.

Hu, X., A. McAlister, J. Kinder, A. Johnson, K. White, C. B. Caruso, C. Wang, R. Basco, C. Gattis, A. Friera, T. Deuse and S. Schrepfer (2025). "Hypoimmune FK binding protein 12-knockout CD19 CAR T cells achieve deep tissue B-cell depletion and control Nalm6 tumors in immunosuppressed humanized mice." Cytotherapy.

Hu, Y., Y. Zhou, M. Zhang, W. Ge, Y. Li, L. Yang, G. Wei, L. Han, H. Wang, S. Yu, Y. Chen, Y. Wang, X. He, X. Zhang, M. Gao, J. Yang, X. Li, J. Ren and H. Huang (2021). "CRISPR/Cas9-Engineered Universal CD19/CD22 Dual-Targeted CAR-T Cell Therapy for Relapsed/Refractory B-cell Acute Lymphoblastic Leukemia." Clin Cancer Res **27**(10): 2764-2772.

Hu, Y., C. Zu, M. Zhang, G. Wei, W. Li, S. Fu, R. Hong, L. Zhou, W. Wu, J. Cui, D. Wang, B. Du, M. Liu, J. Zhang and H. Huang (2023). "Safety and efficacy of CRISPR-based non-viral PD1 locus specifically integrated anti-CD19 CAR-T cells in patients with relapsed or refractory Non-Hodgkin's lymphoma: a first-in-human phase I study." EClinicalMedicine **60**: 102010.

Iyer, S. P., R. A. Sica, P. J. Ho, A. Prica, J. Zain, F. M. Foss, B. Hu, A. Beitinjaneh, W. K. Weng, Y. H. Kim, M. S. Khodadoust, A. O. Huen, L. M. Williams, A. Ma, E. Huang, A. Ganpule, S. D. Nagar, P. Sripakdeevong, E. L. Cullingford, S. Karnik, M. L. Dequeant, J. N. Patel, X. S. He, Z. Li, Q. A. He, J. H. Mendonez, A. Keegan and S. M. Horwitz (2025). "Safety and activity of CTX130, a CD70-targeted allogeneic CRISPR-Cas9-engineered CAR T-cell therapy, in patients with relapsed or refractory T-cell malignancies (COBALT-LYM): a single-arm, open-label, phase 1, dose-escalation study." Lancet Oncol **26**(1): 110-122.

Jain, N., P. Chevallier, H. Liu, G. J. Schiller, J. B. Méar, D. J. DeAngelo, K. J. Curran, S. Grupp, A. Baruchel, M. Balsat, A. LaCroce, C. Roudet, A. B. Korngold, K. J. Newhall, E. Laille, D. J. Lee, M. G. Frattini, R. A. Larson and N. Boissel (2023). "Updated Results of the Phase I BALLI-01 Trial of UCART22 Process 2 (P2), an Anti-CD22 Allogeneic CAR-T Cell Product Manufactured By Cellectis Biologics, in Patients with Relapsed or Refractory (R/R) CD22+ B-Cell Acute Lymphoblastic Leukemia (B-ALL)." Blood **142**: 4847.

Jain, N., G. J. Roboz, M. Konopleva, H. Liu, G. J. Schiller, E. J. Jabbour, D. Whitfield, A. Haider, O. Zernovak, M. G. Frattini, C. Brownstein and R. A. Larson (2021). "Preliminary Results from the Flu/Cy/Alemtuzumab Arm of the Phase I BALLI-01 Trial of UCART22, an Anti-CD22 Allogeneic CAR-T Cell Product, in Adult Patients with Relapsed or Refractory (R/R) CD22+ B-Cell Acute Lymphoblastic Leukemia (B-ALL)." Blood **138**: 1746.

Jambon, S., J. Ruiz, A. Garcia-Maldonado, M. Chen, D. Mendoza, G. Arauz, N. DeMarco, M. Connerney, J. D. Eskew, R. Belani, S. A. Cranert, J. Coronella and D. J. Shedlock (2024). P-CD19CD20-ALLO1: Potent Fully Allogeneic CAR-T Therapy Targeting CD19 and CD20 with Superior Efficacy over Single-Target Products. **144:** 4805.

Jiang, J., J. Chen, C. Liao, Y. Duan, Y. Wang, K. Shang, Y. Huang, Y. Tang, X. Gao, Y. Gu and J. Sun (2023). "Inserting EF1α-driven CD7-specific CAR at CD7 locus reduces fratricide and enhances tumor rejection." Leukemia **37**(8): 1660-1670.

Jing, R., I. Scarfo, M. A. Najia, E. L. da Rocha, A. Han, M. Sanborn, T. Bingham, C. Kubaczka, D. K. Jha, M. Falchetti, T. M. Schlaeger, T. E. North, M. Maus and G. Q. Daley (2022). "EZH1 repression generates mature iPSC-derived CAR T cells with enhanced antitumor activity." Cell Stem Cell **29**(8): 1181-+.

Jo, S., S. Das, A. Williams, A. S. Chretien, T. Pagliardini, A. Le Roy, J. P. Fernandez, D. Le Clerre, B. Jahangiri, I. Chion-Sotinel, S. Rozlan, E. Dessez, A. Gouble, M. Dusséaux, R. Galetto, A. Duclert, E. Marcenaro, R. Devillier, D. Olive, P. Duchateau, L. Poirot and J. Valton (2022). "Endowing universal CAR T-cell with immune-evasive properties using TALEN-gene editing." Nat Commun **13**(1): 3453.

Jung, I. Y., R. L. Bartoszek, A. J. Rech, S. M. Collins, S. K. Ooi, E. F. Williams, C. R. Hopkins, V. Narayan, N. B. Haas, N. V. Frey, E. O. Hexner, D. L. Siegel, G. Plesa, D. L. Porter, A. Cantu, J. K. Everett, S. Guedan, S. L. Berger, F. D. Bushman, F. Herbst and J. A. Fraietta (2023). "Type I Interferon Signaling via the EGR2 Transcriptional Regulator Potentiates CAR T Cell-Intrinsic Dysfunction." Cancer Discov **13**(7): 1636-1655.

Jung, I. Y., Y. Y. Kim, H. S. Yu, M. Lee, S. Kim and J. Lee (2018). "CRISPR/Cas9-Mediated Knockout of DGK Improves Antitumor Activities of Human T Cells." Cancer Res **78**(16): 4692-4703.

Jung, I. Y., V. Narayan, S. McDonald, A. J. Rech, R. Bartoszek, G. Hong, M. M. Davis, J. Xu, A. C. Boesteanu, J. S. Barber-Rotenberg, G. Plesa, S. F. Lacey, J. K. Jadlowsky, D. L. Siegel, D. M. Hammill, P. F. Cho-Park, S. L. Berger, N. B. Haas and J. A. Fraietta (2022). "BLIMP1 and NR4A3 transcription factors reciprocally regulate antitumor CAR T cell stemness and exhaustion." Sci Transl Med **14**(670): eabn7336.

Kagoya, Y., T. Guo, B. Yeung, K. Saso, M. Anczurowski, C. H. Wang, K. Murata, K. Sugata, H. Saijo, Y. Matsunaga, Y. Ohashi, M. O. Butler and N. Hirano (2020). "Genetic Ablation of HLA Class I, Class II, and the T-cell Receptor Enables Allogeneic T Cells to Be Used for Adoptive T-cell Therapy." Cancer Immunol Res **8**(7): 926-936.

Kalaitzidis, D., D. Henderson, S. Karnik, K. Levitsky, B. McEwan, T. Nguyen, Z. Padalia, A. Porras, J. Carson, H. Dar, M. L. Dequeant, L. Kumar, C. Massilamany, P. Tetteh, H. Yu, X. H. Lu, B. Morse, J. Sagert, L. Serwer, S. Y. Tan, H. Waldner, R. Blanchard, J. Terrett and T. Ho (2019). "CRISPR/Cas9 Gene Editing to Produce Multiple Allogeneic CAR-T Cell Candidates Showing Consistently High Potency, Durability, Lack of Alloreactivity, and Ability to Overcome Immune Suppression." Molecular Therapy **27**(4): 388-388.

Kang, L., J. Ma, X. Lou, X. Tang, S. Xue, S. Chen, H. Qiu, M. Miao, Y. Han, Y. Wang, A. Sun, H. Dai, Y. Xu, L. Li, S. Fan, C. Qian, W. Gong, M. Xu, H. Zhou, Z. Yao, W. Wang, M. Li, N. Xu, Z. Yu, D. Wu and L. Yu (2023). "Efficacy and Safety of Interleukin-6-Knockdown CD19-Targeted CAR T Cells(ssCART-19) for Relapsed/Refractory B-ALL." Blood **142**: 889.

Kang, L. Q., X. W. Tang, N. Xu, M. H. Li, J. W. Tan, W. Qi, Z. Yu, X. Y. Lou, S. L. Xue, X. Li, J. Zhang, J. Chen, Z. K. Wang, C. X. Li, F. C. Cheng, D. P. Wu and L. Yu (2019). "shRNA-Interleukin-6 Modified CD19-Specific Chimeric Antigen Receptor T Cell Significantly Improves the Safety in Acute Lymphoblastic Leukemia." Blood **134**.

Kath, J., W. Du, C. Franke, V. Drosdek, V. Glaser, M. Stein, T. Zittel, M. Abou-El-Enein, M. Schmueck-Henneresse, H. D. Volk, P. Reinke and D. L. Wagner (2022). "CD247 (CD3ζ) gene editing as a novel platform technology for off-the-shelf Chimeric Antigen Receptor reprogrammed immune cells." Human Gene Therapy Methods **33**(23-24): A125-A126.

Kath, J., W. Du, A. Pruene, T. Braun, B. Thommandru, R. Turk, M. L. Sturgeon, G. L. Kurgan, L. Amini, M. Stein, T. Zittel, S. Martini, L. Ostendorf, A. Wilhelm, L. Akyüz, A. Rehm, U. E. Höpken, A. Pruß, A. Künkele, A. M. Jacobi, H. D. Volk, M. Schmueck-Henneresse, R. Stripecke, P. Reinke and D. L. Wagner (2022). "Pharmacological interventions enhance virus-free generation of TRAC-replaced CAR T cells." Mol Ther Methods Clin Dev **25**: 311-330.

Keerthi, V., H. Balke-Want, A. Retherford, C. Fowler, P. Xu, N. Gkitsas, K. Asano, S. Patel, Q. Jiang, X. Liu, S. Heitzeneder, R. Tunuguntla, E. Sotillo, J. Sage, C. Mackall and S. Feldman (2024). "B7-H3 CAR T-CELLS MANUFACTURED VIA CRISPR KNOCK-IN AT CLINICAL SCALE ARE EFFECTIVE AGAINST SMALL-CELL LUNG CANCER." Cytotherapy **26**(6): S175-S176.

Kelly, J. J., R. E. Sanchez-Pupo, N. Shalaby, Y. Xia, F. M. Martinez, S. W. McRae, Q. Qi, M. S. Fox, J. W. Hicks, J. D. Thiessen, T. J. Scholl and J. A. Ronald (2023). "Development of a Highly Efficient Modular and Multiplexed CRISPR Editing System for Multimodal Tracking of CAR-T Cells." Molecular Therapy **31**(4): 707-708.

Kienka, T., F. Korell, N. Knudsen, K. Yates, A. D. Cheng, D. Sen, M. Maus and R. Manguso (2024). "Improving CAR T cell efficacy in pancreatic cancer using an in vivo CRISPR Cas9 screen." Cancer Research **84**(6).

Kim, Y., M. Jeun, H. K. Lee, J. U. Choi, S. Park and C. H. Park (2025). "TET2 downregulation enhances the antitumor efficacy of CD19 CAR T cells in a preclinical model." Exp Hematol Oncol **14**(1): 23.

Kinder, J. M., C. Athena-Estrada, D. Malik, X. Hu, M. Lamba, C. Young, N. Van Hoeven, B. Granger, V. Chaturvedi, O. Liang, J. Moreno, A. Lampano, G. Zipp, A. Johnson, T. Fry, S. Schrepfer and A. Foster (2022). "BCMA-Targeted, Hypoimmune Allogeneic CAR T Cells Exhibit Potent Anti-Tumor Activity Together with the Ability to Evade Innate and Adaptive Immune Rejection in Pre-Clinical Tumor Models." Blood **140**: 7120-7121.

Koo, B. U., J. H. Jeong, N. Y. Go, K. I. Lee and J. Y. Lee (2025). "Targeted T cell expressed Diacylglycerol Kinase (DGK)s gene editing enhances Chimeric Antigen Receptor (CAR) T cell durability and efficacy against cancer." Human Gene Therapy Methods **36**(3-4): e385.

Korst, C., C. O'Neill, W. S. C. Bruins, M. Cosovic, I. Twickler, C. P. M. Verkleij, D. Le Clerre, M. Themeli, I. Chion-Sotinel, S. Zweegman, R. Galetto, T. Mutis and N. van de Donk (2024). "Preclinical activity of allogeneic SLAMF7-specific CAR T-cells (UCARTCS1) in multiple myeloma." J Immunother Cancer **12**(7).

Lam, V., J. Barragan, C. Cheung, J. Lu, D. Chian, R. Martinez, C. Sims, P. Sundar, H. Hiraragi, S. Potluri and R. Lynn (2022). "NR4A3 GENE EDITING AND C-JUN OVEREXPRESSION SYNERGIZE TO LIMIT EXHAUSTION AND ENHANCE FUNCTIONAL ACTIVITY OF ROR1 CAR T CELLS IN VITRO AND IN VIVO." Journal for ImmunoTherapy of Cancer **10**: A257-A258.

Lam, V. C., A. Li, M. G. Casas, J. Barragan, C. Cheung, J. Briones, E. Afreen, G. Vavra, J. Lu, P. Sundar, R. Martinez, C. Sims, C. Ta, E. Nigatu, S. Potluri, O. Ali, A. S. Cheung and R. C. Lynn (2023). "PRECLINICAL DEVELOPMENT OF LYL119, A ROR1- TARGETED CAR T-CELL PRODUCT INCORPORATING FOUR NOVEL T-CELL REPROGRAMMING TECHNOLOGIES TO OVERCOME BARRIERS TO EFFECTIVE CELL THERAPY FOR SOLID TUMORS." Journal for ImmunoTherapy of Cancer **11**: A318-A319.

Lau, E., G. Kwong, T. W. Fowler, B. C. Sun, P. D. Donohoue, R. T. Davis, M. Bryan, S. McCawley, S. C. Clarke, C. Williams, L. Banh, M. Irby, L. Edwards, M. Storlie, B. Kohrs, G. W. J. Lilley, S. C. Smith, S. Gradia, C. K. Fuller, J. Skoble, E. Garner, M. van Overbeek and S. B. Kanner (2023). "Allogeneic chimeric antigen receptor-T cells with CRISPR-disrupted programmed death-1 checkpoint exhibit enhanced functional fitness." Cytotherapy **25**(7): 750-762.

Lee, Y. H., H. J. Lee, H. C. Kim, Y. Lee, S. K. Nam, C. Hupperetz, J. S. Y. Ma, X. Wang, O. Singer, W. S. Kim, S. J. Kim, Y. Koh, I. Jung and C. H. Kim (2022). "PD-1 and TIGIT downregulation distinctly affect the effector and early memory phenotypes of CD19-targeting CAR T cells." Mol Ther **30**(2): 579-592.

Lei, K., J. Li, Z. Tu, C. Gong, J. Liu, M. Luo, W. Ai, L. Wu, Y. Li, Z. Zhou, Z. Chen, S. Lv, M. Ye, M. Wu, X. Long, X. Zhu and K. Huang (2024). "Endosome-microautophagy targeting chimera (eMIATAC) for targeted proteins degradation and enhance CAR-T cell anti-tumor therapy." Theranostics **14**(11): 4481-4498.

Lekakis, L. J., F. L. Locke, M. Tees, S. S. Neelapu, S. A. Malik, M. Hamadani, M. J. Frank, L. L. Popplewell, J. S. Abramson, S. de Vos, J. Munoz, C. R. Shin, A. Balakumaran, L. Loomis-Navale, L. Goyal, X. Zhou and D. B. Miklos (2021). "ALPHA2 Study: ALLO-501A Allogeneic CAR T in LBCL, Updated Results Continue to Show Encouraging Safety and Efficacy with Consolidation Dosing." Blood **138**: 649.

Li, L. L., Y. P. Xia, Q. Li, P. Wang, P. P. Sun, X. G. Wang and R. Zhang (2024). "Case report: CD7-targeted autologous CAR-T therapy for the treatment of T-cell acute lymphoblastic leukemia undergoing allogeneic peripheral blood stem cell transplantation in the long-term follow-up." Front Immunol **15**: 1469251.

Li, N., N. Tang, C. Cheng, T. Hu, X. Wei, W. Han and H. Wang (2020). "Improving the anti-solid tumor efficacy of CAR-T cells by inhibiting adenosine signaling pathway." Oncoimmunology **9**(1): 1824643.

Li, P., Q. Su, Y. Xu, J. He, L. Wang, Y. Wang, H. Li, K. Lan, H. Zheng, D. Zhu, L. Zhang, Y. Zhang, J. Chen and X. Mou (2024). "Efficient Genome Editing of Human T Cells with Innovative Transformer Base Editor (tBE) for Construction of Next Generation CAR-T Therapies." Molecular Therapy **32**(4): 626.

Li, W., X. Zhu, Y. Xu, J. Chen, H. Zhang, Z. Yang, Y. Qi, J. Hong, Y. Li, G. Wang, J. Shen and C. Qian (2022). "Simultaneous editing of TCR, HLA-I/II and HLA-E resulted in enhanced universal CAR-T resistance to allo-rejection." Front Immunol **13**: 1052717.

Lin, F., S. Yamada-Hunter, A. Croft, N. DeNamur, E. Tokuda, M. Libbrecht, N. Chartrand, Y. Chen, P. Xu, A. Seki, E. Li, J. Dean, K. Freitas, L. Thompson, B. Sather, C. Mackall and E. Sotillo (2025). "Combined silencing of MED12 and activation of IL2 by epigenetic editing enhances CAR T antitumor potency." Cancer Research **85**(8).

Lin, Y. G., H. B. Yin, C. Zhou, L. Zhou, Y. Y. Zeng and H. R. Yao (2024). "Phase I clinical trial of MUC1-targeted CAR-T cells with PD-1-knockout in the treatment of advanced breast cancer." Journal of Clinical Oncology **42**(16).

Liu, F., Y. Guo, W. Y. Yang, Y. Wang and X. F. Zhu (2023). "Gene-Edited Universal CD7 Chimeric Antigen Receptor T Cells for Pediatric T-Cell Acute Lymphoblastic Leukemia or Lymphoblastic Lymphoma." Blood **142**.

Liu, G., Q. Zhang, G. Liu, D. Li, L. Zhang, Z. Gu, H. Tian, Y. Zhang and X. Tian (2021). "Disruption of adenosine 2A receptor improves the anti-tumor function of anti-mesothelin CAR T cells both in vitro and in vivo." Exp Cell Res **409**(1): 112886.

Liu, X., Y. Zhang, C. Cheng, A. W. Cheng, X. Zhang, N. Li, C. Xia, X. Wei, X. Liu and H. Wang (2017). "CRISPR-Cas9-mediated multiplex gene editing in CAR-T cells." Cell Res **27**(1): 154-157.

Liu, Y., J. H. Shi, Q. M. Yang, C. M. Wang, F. F. Liang, Z. G. Wu, Q. Z. Lamao, J. X. Yang, Y. Y. Qiu, W. Tang, H. X. Zhang, Z. H. Yang, L. Yang, Y. J. Zhang, M. Jin, P. F. Yuan, W. S. Wei and W. D. Han (2024). "The interim analysis of a first-in-human phase 1 trial of ET-901, a CRISPR edited allogeneic immune-cloaked anti-CD19 CAR-T cell therapy in patients with r/r B-NHL." Journal of Clinical Oncology **42**(16).

Liu, Z. F., L. Y. Chen, J. Wang, L. Q. Kang, H. Tang, Y. Zhou, H. X. Zhou, A. N. Sun, D. P. Wu and S. L. Xue (2020). "Successful treatment of acute B lymphoblastic leukemia relapse in the skin and testicle by anti-CD19 CAR-T with IL-6 knocking down: a case report." Biomark Res **8**: 12.

Lonez, C., J. Bolsée, F. Huberty, T. Nguyen, C. Jacques-Hespel, S. Anguille, A. Flament and E. Breman (2025). "Clinical Proof-of-Concept of a Non-Gene Editing Technology Using miRNA-Based shRNA to Engineer Allogeneic CAR T-Cells." Int J Mol Sci **26**(4).

Lu, D., H. Y. Chu, S. Park, M. Landon, M. Tsuda, E. Avramis, C. Dege, T. Dailey, Y. Pan, S. K. Hanok, M. Denholtz, R. Abujarour, T. Lee, J. Goulding, M. Hosking, J. Goodridge, E. Peralta and B. Valamehr (2025). "A novel CD3ε fusion receptor allows T cell engager use in TCR-less allogeneic CAR T cells to improve activity and prevent antigen escape." Mol Ther.

Lu, J., V. C Lam, C. Cheung, L. Zhao, S. Siebert, K. Xiong, S. Cheemalamarri, C. Ta, R. Martinez, P. Sundar, R. C. Lynn and S. Potluri (2024). "MULTIOMIC PROFILING OF LYL119: A REPROGRAMMED ROR1 CAR-T PRODUCT GENERATES T CELLS WITH REDUCED EXHAUSTION AND ENHANCED MEMORY CHARACTERISTICS ASSOCIATED WITH INCREASED AP-1 AND REDUCED NR4A BINDINGS." Journal for ImmunoTherapy of Cancer **12**: A322.

Lu, Q., H. Li, Z. Wu, Z. Zhu, Z. Zhang, D. Yang and A. Tong (2024). "BCMA/CD47-directed universal CAR-T cells exhibit excellent antitumor activity in multiple myeloma." J Nanobiotechnology **22**(1): 279.

Lu, Q., D. Yang, H. Li, Z. Zhu, Z. Zhang, Y. Chen, N. Yang, J. Li, Z. Wang, T. Niu and A. Tong (2024). "Delivery of CD47-SIRPα checkpoint blocker by BCMA-directed UCAR-T cells enhances antitumor efficacy in multiple myeloma." Cancer Lett **585**: 216660.

Ma, Y. J., H. P. Dai, Q. Y. Cui, W. Cui, W. J. Zhu, C. J. Qu, L. Q. Kang, M. Q. Zhu, X. M. Zhu, D. D. Liu, Y. F. Feng, H. J. Shen, T. H. Liu, H. Y. Qiu, L. Yu, D. P. Wu and X. W. Tang (2022). "Successful application of PD-1 knockdown CLL-1 CAR-T therapy in two AML patients with post-transplant relapse and failure of anti-CD38 CAR-T cell treatment." Am J Cancer Res **12**(2): 615-621.

MacLeod, D. T., J. Antony, A. J. Martin, R. J. Moser, A. Hekele, K. J. Wetzel, A. E. Brown, M. A. Triggiano, J. A. Hux, C. D. Pham, V. V. Bartsevich, C. A. Turner, J. Lape, S. Kirkland, C. W. Beard, J. Smith, M. L. Hirsch, M. G. Nicholson, D. Jantz and B. McCreedy (2017). "Integration of a CD19 CAR into the TCR Alpha Chain Locus Streamlines Production of Allogeneic Gene-Edited CAR T Cells." Mol Ther **25**(4): 949-961.

Mai, D., T. Boyce, A. Mehta, J. Reff, J. Scholler, N. C. Sheppard and C. H. June (2024). "ZFP36 disruption is insufficient to enhance the function of mesothelin-targeting human CAR-T cells." Sci Rep **14**(1): 3113.

Mailankody, S., M. Liedtke, S. Sidana, J. V. Matous, S. Chhabra, O. O. Oluwole, S. A. Malik, S. Kumar, R. Nath, F. Anwer, J. C. Cruz, S. Jagannath, M. Htut, N. S. Raje, D. S. Siegel, E. E. Karski, W. Lovelace, A. Lourbakos, S. Ponnathapura Nandakumar, A. Balakumaran and P. Hari (2021). "Universal Updated Phase 1 Data Validates the Feasibility of Allogeneic Anti-BCMA ALLO-715 Therapy for Relapsed/Refractory Multiple Myeloma." Blood **138**: 651.

Maldini, C. R., A. C. Messana, P. B. Bendet, A. J. Camblin, F. M. Musenge, M. L. White, J. J. Rocha, L. J. Coholan, C. Karaca, F. Li, B. Yan, V. D. Vrbanac, E. Marte, D. T. Claiborne, C. L. Boutwell and T. M. Allen (2024). "Immunosuppressant therapy averts rejection of allogeneic FKBP1A-disrupted CAR-T cells." Mol Ther **32**(10): 3485-3503.

Maldonado-Pérez, N., M. Tristán-Manzano, P. Justicia-Lirio, E. Martínez-Planes, P. Muñoz, K. Pavlovic, M. Cortijo-Gutiérrez, C. Blanco-Benítez, M. Castella, M. Juan, M. Wenes, P. Romero, F. J. Molina-Estévez, C. Marañón, C. Herrera, K. Benabdellah and F. Martin (2022). "Efficacy and safety of universal (TCRKO) ARI-0001 CAR-T cells for the treatment of B-cell lymphoma." Front Immunol **13**: 1011858.

Mao, Y. and X. Xu (2021). "Cytokine Release Syndrome after Treatment of Anti-CD19 CAR-T Therapy with IL-6 Knocking Down in Patients with Central Nervous System B-cell Acute Lymphocytic Leukemia." Ann Clin Lab Sci **51**(6): 790-794.

Mathur, R., Z. Zhang, J. He, R. Galetto, A. Gouble, I. Chion-Sotinel, S. Filipe, A. Gariboldi, T. Veeramachaneni, E. Manasanch, S. Thomas, H. C. Lee, K. Patel, D. Weber, R. E. Davis, R. Orlowski, J. Smith, J. Yang and S. S. Neelapu (2018). "Targeting multiple myeloma with universal SLAMF7-specific CAR T-cells." Cancer Immunology Research **6**(9).

Mavuluri, J. (2022). "Genetically knock out of PSGL-1 increases CD19-CAR-T cells persistence in mouse preB-ALL disease model." Journal of Immunology **208**(1).

McEwan, B., Z. Padalia, A. Porras, J. Sagert, J. A. Terrett, T. Ho and D. Kalaitzidis (2019). "Allogeneic CRISPR/Cas9 gene-edited CAR-T cells targeting CD33 show potent preclinical activity against AML cells." Cancer Research **79**(13).

McGuirk, J. P., C. S. Tam, N. Kröger, P. A. Riedell, H. S. Murthy, P. J. Ho, J. E. Maakaron, E. K. Waller, F. T. Awan, P. J. Shaughnessy, A. Ghobadi, M. R. Bishop, A. Alfonso-Pierola, M. Dickinson, P. R. Geethakumari, A. Ross, W. Stevens, H. S. Xu, A. N. Ma, S. B. Cohen, R. T. Maziarz and C. Bachier (2022). "CTX110 Allogeneic CRISPR-Cas9-Engineered CAR T Cells in Patients (Pts) with Relapsed or Refractory (R/R) Large B-Cell Lymphoma (LBCL): Results from the Phase 1 Dose Escalation Carbon Study." Blood **140**: 10303-10306.

Menegatti, S., S. Lopez-Cobo, A. Sutra Del Galy, J. Fuentealba, L. Silva, L. Perrin, S. Heurtebise-Chrétien, V. Pottez-Jouatte, A. Darbois, N. Burgdorf, A. L. Privat, A. Simon, M. Laprie-Sentenac, M. Saitakis, B. Wick, B. R. Webber, B. S. Moriarity, O. Lantz, S. Amigorena and L. Menger (2024). "Ablation of FAS confers allogeneic CD3(-) CAR T cells with resistance to rejection by T cells and natural killer cells." Nat Biomed Eng **8**(12): 1651-1664.

Mise-Omata, S., M. Ando, T. Srirat, K. Nakagawara, T. Hayakawa, M. Iizuka-Koga, H. Nishimasu, O. Nureki, M. Ito and A. Yoshimura (2023). "SOCS3 deletion in effector T cells confers an anti-tumorigenic role of IL-6 to the pro-tumorigenic cytokine." Cell Rep **42**(8): 112940.

Nakagawara, K., M. Ando, T. Srirat, S. Mise-Omata, T. Hayakawa, M. Ito, K. Fukunaga and A. Yoshimura (2024). "NR4A ablation improves mitochondrial fitness for long persistence in human CAR-T cells against solid tumors." J Immunother Cancer **12**(8).

Nakashima, T., T. Ouchida, Y. Ishikawa, Y. Ito, T. Hayakawa, T. Yoshikawa, H. Zhang, H. Kasuya, Y. Li, T. Matsukawa, S. Inoue, S. Iida, H. Kiyoi and Y. Kagoya (2025). "JAK-STAT-activated, fratricide-resistant CAR-T cells targeting membrane-bound TNF effectively treat AML and solid tumors." J Immunother Cancer **13**(7).

Neelapu, S. S., R. Nath, J. Munoz, M. Tees, D. B. Miklos, M. J. Frank, S. A. Malik, D. Stevens, C. R. Shin, A. Balakumaran, L. Loomis-Navale, L. Goyal, A. Nguyen and F. L. Locke (2021). "ALPHA Study: ALLO-501 Produced Deep and Durable Responses in Patients with Relapsed/Refractory Non-Hodgkin's Lymphoma Comparable to Autologous CAR T." Blood **138**: 3878.

Ottaviano, G., C. Georgiadis, S. A. Gkazi, F. Syed, H. Zhan, A. Etuk, R. Preece, J. Chu, A. Kubat, S. Adams, P. Veys, A. Vora, K. Rao and W. Qasim (2022). "Phase 1 clinical trial of CRISPR-engineered CAR19 universal T cells for treatment of children with refractory B cell leukemia." Sci Transl Med **14**(668): eabq3010.

Oumzil, I., V. Guerrero, P. S. Keerthipati, J. Colangelo, B. Lawler, V. Singson, A. Khedkar, S. Singh, J. Terrett and D. Hostetter (2022). "CRISPR/CAS9 GENE-EDITED, ALLOGENEIC ANTI-CD83 CAR-T CELLS DEMONSTRATE POTENT ACTIVITY IN GVHD AND AML TUMOR MODELS." Journal for Immunotherapy of Cancer **10**: A386-A386.

Ouyang, W., S. W. Jin, N. Xu, W. Y. Liu, H. Zhao, L. Zhang, L. Kang, Y. Tao, Y. Liu, Y. Wang, J. Wang, F. Liu, L. Yu, Z. Liu and J. Q. Mi (2024). "PD-1 downregulation enhances CAR-T cell antitumor efficiency by preserving a cell memory phenotype and reducing exhaustion." J Immunother Cancer **12**(4).

Pal, S. K., B. Tran, J. Haanen, M. E. Hurwitz, A. Sacher, N. M. Tannir, L. E. Budde, S. J. Harrison, S. Klobuch, S. S. Patel, L. Meza, M. L. Dequeant, A. Ma, Q. A. He, L. M. Williams, A. Keegan, E. B. Gurary, H. Dar, S. Karnik, C. Guo, H. Heath, R. R. Yuen, P. K. Morrow, N. Agarwal and S. A. Srour (2024). "CD70-Targeted Allogeneic CAR T-Cell Therapy for Advanced Clear Cell Renal Cell Carcinoma." Cancer Discov **14**(7): 1176-1189.

Pan, J., Y. Tan, L. Shan, S. Seery, B. Deng, Z. Ling, J. Xu, J. Duan, Z. Wang, K. Wang, X. Yu, Q. Zheng, X. Xu, G. Hu, T. Tan, Y. Yuan, Z. Tian, F. Yan, Y. Han, J. Zhang and X. Feng (2025). "Allogeneic CD5-specific CAR-T therapy for relapsed/refractory T-ALL: a phase 1 trial." Nat Med **31**(1): 126-136.

Panowski, S. H., S. Srinivasan, N. Tan, S. K. Tacheva-Grigorova, B. Smith, Y. Mak, H. Ning, J. Villanueva, D. Wijewarnasuriya, S. Lang, Z. Melton, A. Ghosh, M. Dusseaux, R. Galetto, J. R. Heyen, T. Sai, T. J. Van Blarcom, J. Chaparro-Riggers and B. J. Sasu (2022). "Preclinical Development and Evaluation of Allogeneic CAR T Cells Targeting CD70 for the Treatment of Renal Cell Carcinoma." Cancer Res.

Park, A., I. Monroy, C. Cook, S. He, K. Karasiewicz, M. Shah, N. Withana, L. Chong, R. Hariri, Y. Fong and S. Priceman (2022). "CF33-CD19T ONCOLYTIC VIRUS (ONCARLYTICS) IN COMBINATION WITH OFF-THE-SHELF ALLOGENEIC CYCART-19 T CELLS TARGETING DE NOVO CD19+ SOLID TUMORS." Journal for ImmunoTherapy of Cancer **10**: A884.

Patel, R. P., G. Ghilardi, P. Porazzi, S. Yang, D. A. Qian, R. Pajarillo, M. Wang, Y. L. Zhang, S. J. Schuster, S. K. Barta, A. Nimmagadda, A. Snook, N. A. Siciliano and M. Ruella (2022). "Clinical Development of Senza5™ CART5: a Novel Dual Population CD5 CRISPR-Cas9 Knocked out Anti-CD5 Chimeric Antigen Receptor T Cell Product for Relapsed and Refractory CD5+Nodal T-Cell Lymphomas." Blood **140**: 1604-1605.

Patel, R. P., G. Ghilardi, Y. Zhang, Y. H. Chiang, W. Xie, P. Guruprasad, K. H. Kim, I. Chun, M. G. Angelos, R. Pajarillo, S. J. Hong, Y. G. Lee, O. Shestova, C. Shaw, I. Cohen, A. Gupta, T. Vu, D. Qian, S. Yang, A. Nimmagadda, A. E. Snook, N. Siciliano, A. Rotolo, A. Inamdar, J. Harris, O. Ugwuanyi, M. Wang, A. Carturan, L. Paruzzo, L. Chen, H. J. Ballard, T. Blanchard, C. Xu, M. Abdel-Mohsen, K. Gabunia, M. Wysocka, G. P. Linette, B. Carreno, D. M. Barrett, D. T. Teachey, A. D. Posey, D. J. Powell, Jr., C. T. Sauter, S. Pileri, V. Pillai, J. Scholler, A. H. Rook, S. J. Schuster, S. K. Barta, P. Porazzi and M. Ruella (2024). "CD5 deletion enhances the antitumor activity of adoptive T cell therapies." Sci Immunol **9**(97): eadn6509.

Peng, Z., Z. Yao, L. Yi, K. Kang, R. Luo, S. Liu, Y. Zhang, S. Liao, F. Na, L. Xiang, R. Tong and Y. Lu (2025). "Enhancing the persistence of universal CAR-T against DLL3 positive small cell lung cancer using the epigenetic regulation system mediated by CRISPR." Cancer Research **85**(8).

Petersen, C., M. Bell, H. Houke, Z. Z. Yi, S. Gottschalk and G. Krenciute (2019). "CRISPR/CAS9-MEDIATED SILENCING OF SHP-1 SIGNIFICANTLY ENHANCES THE ANTI-GLIOMA ACTIVITY OF IL-13RA2 CAR T CELLS." Neuro-Oncology **21**: 95-96.

Poirot, L., B. Philip, C. Schiffer-Mannioui, D. Le Clerre, I. Chion-Sotinel, S. Derniame, P. Potrel, C. Bas, L. Lemaire, R. Galetto, C. Lebuhotel, J. Eyquem, G. W. Cheung, A. Duclert, A. Gouble, S. Arnould, K. Peggs, M. Pule, A. M. Scharenberg and J. Smith (2015). "Multiplex Genome-Edited T-cell Manufacturing Platform for "Off-the-Shelf" Adoptive T-cell Immunotherapies." Cancer Res **75**(18): 3853-3864.

Preece, R., O. Gough, R. Kadirkamanathan, E. Cudworth, N. Pina, D. Kallon, C. Georgiadis and W. Qasim (2025). "Base-edited CAR38 T cells evade fratricide and immune rejection while delivering potent anti-leukemic affects." Human Gene Therapy Methods **36**(3-4): e138-e139.

Prinzing, B., C. C. Zebley, C. T. Petersen, Y. Fan, A. A. Anido, Z. Yi, P. Nguyen, H. Houke, M. Bell, D. Haydar, C. Brown, S. K. Boi, S. Alli, J. C. Crawford, J. M. Riberdy, J. J. Park, S. Zhou, M. P. Velasquez, C. DeRenzo, C. R. Lazzarotto, S. Q. Tsai, P. Vogel, S. M. Pruett-Miller, D. M. Langfitt, S. Gottschalk, B. Youngblood and G. Krenciute (2021). "Deleting DNMT3A in CAR T cells prevents exhaustion and enhances antitumor activity." Sci Transl Med **13**(620): eabh0272.

Qasim, W., H. Zhan, S. Samarasinghe, S. Adams, P. Amrolia, S. Stafford, K. Butler, C. Rivat, G. Wright, K. Somana, S. Ghorashian, D. Pinner, G. Ahsan, K. Gilmour, G. Lucchini, S. Inglott, W. Mifsud, R. Chiesa, K. S. Peggs, L. Chan, F. Farzeneh, A. J. Thrasher, A. Vora, M. Pule and P. Veys (2017). "Molecular remission of infant B-ALL after infusion of universal TALEN gene-edited CAR T cells." Sci Transl Med **9**(374).

Qiao, Y., J. Chen, X. Wang, S. Yan, J. Tan, B. Xia, Y. Chen, K. Lin, F. Zou, B. Liu, X. He, Y. Zhang, X. Zhang, H. Zhang, X. Wu and L. Lu (2023). "Enhancement of CAR-T cell activity against cholangiocarcinoma by simultaneous knockdown of six inhibitory membrane proteins." Cancer Commun (Lond) **43**(7): 788-807.

Rasaiyaah, J., C. Georgiadis, R. Preece, U. Mock and W. Qasim (2018). "TCRαβ/CD3 disruption enables CD3-specific antileukemic T cell immunotherapy." JCI Insight **3**(13).

Ren, J., X. Liao, J. M. Lewis, J. Chang, R. Qu, K. R. Carlson, F. Foss and M. Girardi (2024). "Generation and optimization of off-the-shelf immunotherapeutics targeting TCR-Vβ2+ T cell malignancy." Nat Commun **15**(1): 519.

Ren, J., X. Liu, C. Fang, S. Jiang, C. H. June and Y. Zhao (2017). "Multiplex Genome Editing to Generate Universal CAR T Cells Resistant to PD1 Inhibition." Clin Cancer Res **23**(9): 2255-2266.

Richter, M., S. Cranert, Y. Tan, M. Tong, C. Domingo, E. Argus, S. Ibitokou, J. Smith, C. Martin, X. Wang, B. Barnett, E. Ostertag, J. Coronella and D. Shedlock (2019). "P-BCMA-ALLO1 - a nonviral, allogeneic anti-BCMA CAR-T therapy with potent antitumor function for the treatment of multiple myeloma." Journal for ImmunoTherapy of Cancer **7**.

Roders, N., C. Nakid-Cordero, F. Raineri, M. Fayon, A. Abecassis, C. Choisy, E. Nelson, C. Maillard, D. Garrick, A. Talbot, J. P. Fermand, B. Arnulf and J. C. Bories (2024). "Dual Chimeric Antigen Receptor T Cells Targeting CD38 and SLAMF7 with Independent Signaling Demonstrate Preclinical Efficacy and Safety in Multiple Myeloma." Cancer Immunol Res **12**(4): 478-490.

Rupp, L. J., K. Schumann, K. T. Roybal, R. E. Gate, C. J. Ye, W. A. Lim and A. Marson (2017). "CRISPR/Cas9-mediated PD-1 disruption enhances anti-tumor efficacy of human chimeric antigen receptor T cells." Sci Rep **7**(1): 737.

Salim, S. K., V. Shaikh, W. T. Maich, A. Anand, B. Brakel, J. Wei, M. K. Subapanditha, Z. Alizada, Y. Suk, M. Singh, V. Dimitrov, Z. Tabunshchyk, K. Chen, P. Vora, C. Venugopal, J. Moffat and S. K. Singh (2023). "AUTOLOGOUS PATIENT-DERIVED CAR-TS SHOW FUNCTIONAL DEFICITS AGAINST HUMAN GLIOBLASTOMA." Neuro-Oncology Advances **5**: ii6.

Sallman, D. A., D. J. DeAngelo, N. Pemmaraju, S. Dinner, S. Gill, R. L. Olin, E. S. Wang, M. Konopleva, E. Stark, A. Korngold, A. Haider, K. Backhouse, C. Figliola, D. J. Lee, M. G. Frattini, C. Brownstein and G. J. Roboz (2022). "Ameli-01: A Phase I Trial of UCART123v1.2, an Anti-CD123 Allogeneic CAR-T Cell Product, in Adult Patients with Relapsed or Refractory (R/R) CD123+ Acute Myeloid Leukemia (AML)." Blood **140**: 2371-2373.

Sanber, K., Z. Nawas, V. Salsman, A. Gad, P. Matthew, C. Lee, S. Joseph, N. Ahmed and M. Hegde (2020). "Modulation of inhibitory signals in CAR T cells leads to improved activity against glioblastoma." Journal of Clinical Oncology **38**(15).

Schiffer-Mannioui, C., S. Leduc, I. Chion-Sotinel, D. Le Clerre, V. Guyot, M. Rotondi, R. Galetto and A. Gouble (2021). "Mesothelin (MSLN) targeting allogeneic car T cells engineered to overcome tumor immunosuppressive microenvironment." Journal for ImmunoTherapy of Cancer **9**(SUPPL 2): A151.

Senz, A. M., P. Metzger, R. K. Rubens, B. Cadilha, M. Kirmaier, S. Lesch, M. R. Benmebarek, S. Theurich, P. Murray, S. Endres, S. Kobold, L. M. König, P. Duewell and M. Schnurr (2020). "Ido1-deleted car T cells show improved therapeutic efficacy in murine pancreatic cancer models." Journal for ImmunoTherapy of Cancer **8**(SUPPL 2): A43.

Shang, K., D. Huang, J. Liu, Z. Yu, W. Bian, J. Chen, Y. Zhao, L. Liu, J. Jiang, Y. Wang, Y. Duan, J. Ge, S. Zhang, C. Zhou, Y. Han, Y. Hu, W. Zheng, J. Sun, H. Huang, S. Pei, P. Qian and J. Sun (2025). "CD97-directed CAR-T cells with enhanced persistence eradicate acute myeloid leukemia in diverse xenograft models." Cell Rep Med **6**(6): 102148.

Shi, J., Z. Zhang, H. Y. Chen, Y. Yao, S. Ke, K. Yu, J. Shi, X. Xiao, C. He, B. Xiang, Y. Sun, M. Gao, X. Xing, H. Yu, X. Wang, W. C. Yuan, B. R. Budiarto, S. Y. Chen, T. Zhang, Y. R. Lee, H. Zhu and J. Zhang (2025). "Targeting the TRIM21-PD-1 axis potentiates immune checkpoint blockade and CAR-T cell therapy." Mol Ther **33**(3): 1073-1090.

Shirinbak, S., J. Grant, W. I. Yeh, A. Gentile, B. Shrestha, B. H. Yang, M. Pribadi, S. Sikaroodi, A. Mehta, A. D. Yzaguirre, Y. Pan, C. W. Chang, T. Lee, L. Smith, J. Wu, B. Walcheck, M. Hosking and B. Valamehr (2022). "Multi-Antigen Targeting By Novel Combination of CAR-T Cells and hnCD16 Transgene, Yields in Complete Tumor Clearance Via Antibody Dependent Cellular Cytotoxicity." Blood **140**: 10253-10254.

Sodji, Q. H., A. Shea, D. Cappabianca, M. H. Forsberg, J. C. Eickhoff, M. B. Idrissou, A. S. Ollendorff, O. Kwon, I. M. Ong, R. Hernandez, J. Weichert, B. P. Bednarz, K. Saha, P. M. Sondel, C. M. Capitini and Z. S. Morris (2025). "Low-dose radiation by radiopharmaceutical therapy enhances GD2 TRAC-CAR T cell efficacy in localized neuroblastoma." Sci Adv **11**(23): eadu4417.

Soltantoyeh, T., B. Akbari, Z. Shahosseini, H. R. Mirzaei and J. Hadjati (2024). "Simultaneous targeting of Tim3 and A2a receptors modulates MSLN-CAR T cell antitumor function in a human cervical tumor xenograft model." Front Immunol **15**: 1362904.

Sommer, C., B. Boldajipour, T. C. Kuo, T. Bentley, J. Sutton, A. Chen, T. Geng, H. Dong, R. Galetto, J. Valton, T. Pertel, A. Juillerat, A. Gariboldi, E. Pascua, C. Brown, S. M. Chin, T. Sai, Y. Ni, P. Duchateau, J. Smith, A. Rajpal, T. Van Blarcom, J. Chaparro-Riggers and B. J. Sasu (2019). "Preclinical Evaluation of Allogeneic CAR T Cells Targeting BCMA for the Treatment of Multiple Myeloma." Mol Ther **27**(6): 1126-1138.

Stenger, D., T. A. Stief, T. Kaeuferle, S. Willier, F. Rataj, K. Schober, B. Vick, R. Lotfi, B. Wagner, T. G. P. Grünewald, S. Kobold, D. H. Busch, I. Jeremias, F. Blaeschke and T. Feuchtinger (2020). "Endogenous TCR promotes in vivo persistence of CD19-CAR-T cells compared to a CRISPR/Cas9-mediated TCR knockout CAR." Blood **136**(12): 1407-1418.

Stepanova, V. M., D. V. Volkov, D. S. Osipova, W. Wang, Y. Hou, D. E. Pershin, M. S. Fadeeva, E. A. Malakhova, E. A. Kulakovskaya, L. Cuicui, Z. Mingfeng, H. Zhang, J. Xie, D. Zhang, I. Z. Mamedov, A. S. Chernov, G. B. Telegin, Y. P. Rubtsov, A. G. Gabibov, P. Wu, M. A. Maschan and A. V. Stepanov (2024). "Targeting CD45 by gene-edited CAR T cells for leukemia eradication and hematopoietic stem cell transplantation preconditioning." Mol Ther Oncol **32**(3): 200843.

Sterner, R. M., R. Sakemura, M. J. Cox, N. Yang, R. H. Khadka, C. L. Forsman, M. J. Hansen, F. Jin, K. Ayasoufi, M. Hefazi, K. J. Schick, D. K. Walters, O. Ahmed, D. Chappell, T. Sahmoud, C. Durrant, W. K. Nevala, M. M. Patnaik, L. R. Pease, K. E. Hedin, N. E. Kay, A. J. Johnson and S. S. Kenderian (2019). "GM-CSF inhibition reduces cytokine release syndrome and neuroinflammation but enhances CAR-T cell function in xenografts." Blood **133**(7): 697-709.

Stewart, C. M., E. L. Siegler, R. L. Sakemura, M. J. Cox, T. Huynh, B. Kimball, L. Mai, I. Can, C. Manriquez Roman, K. Yun, O. Sirpilla, J. H. Girsch, E. Ogbodo, W. Mohammed Ismail, A. Gaspar-Maia, J. Budka, J. Kim, N. Scholler, M. Mattie, S. Filosto and S. S. Kenderian (2024). "IL-4 drives exhaustion of CD8(+) CART cells." Nat Commun **15**(1): 7921.

Su, Q., J. Yao, M. A. Farooq, I. Ajmal, Y. Duan, C. He, X. Hu and W. Jiang (2024). "Modulating Cholesterol Metabolism via ACAT1 Knockdown Enhances Anti-B-Cell Lymphoma Activities of CD19-Specific Chimeric Antigen Receptor T Cells by Improving the Cell Activation and Proliferation." Cells **13**(6).

Sugita, M., R. Galetto, H. Zong, N. Ewing-Crystal, V. Trujillo-Alonso, N. Mencia-Trinchant, W. Yip, S. Filipe, C. Lebuhotel, A. Gouble, D. C. Hassane, J. Smith, G. J. Roboz and M. L. Guzman (2022). "Allogeneic TCRαβ deficient CAR T-cells targeting CD123 in acute myeloid leukemia." Nat Commun **13**(1): 2227.

Tang, N., C. Cheng, X. Zhang, M. Qiao, N. Li, W. Mu, X. F. Wei, W. Han and H. Wang (2020). "TGF-β inhibition via CRISPR promotes the long-term efficacy of CAR T cells against solid tumors." JCI Insight **5**(4).

Tang, Y., W. Liu, S. Kadu, O. Johnson, Z. S. Hasanali, A. Kelly, A. Shestov, R. Pajarillo, E. Greenblatt, M. Holmes, L. P. Wang, N. Shih, R. S. O'Connor, M. Ruella, A. L. Garfall, D. Allman, D. T. Vogl, A. Cohen, C. H. June and N. C. Sheppard (2024). "Exploiting the CD200-CD200R immune checkpoint axis in multiple myeloma to enhance CAR T-cell therapy." Blood **143**(2): 139-151.

Tipanee, J., E. Samara-Kuko, T. Gevaert, M. K. Chuah and T. VandenDriessche (2022). "Universal allogeneic CAR T cells engineered with Sleeping Beauty transposons and CRISPR-CAS9 for cancer immunotherapy." Mol Ther **30**(10): 3155-3175.

Trefny, M. P., N. Kirchhammer, P. Auf der Maur, M. Natoli, D. Schmid, M. Germann, L. Fernandez Rodriguez, P. Herzig, J. Lötscher, M. Akrami, J. C. Stinchcombe, M. A. Stanczak, A. Zingg, M. Buchi, J. Roux, R. Marone, L. Don, D. Lardinois, M. Wiese, L. T. Jeker, M. Bentires-Alj, J. Rossy, D. S. Thommen, G. M. Griffiths, H. Läubli, C. Hess and A. Zippelius (2023). "Deletion of SNX9 alleviates CD8 T cell exhaustion for effective cellular cancer immunotherapy." Nat Commun **14**(1): 86.

Vale, P. D. S. and N. Gascoigne (2025). "DISRUPTED KINASE (DISK) CAR-T CELLS FOR ALLOGENEIC THERAPY." Human Gene Therapy Methods **36**(5-6): e666.

Wang, D., B. C. Prager, R. C. Gimple, B. Aguilar, D. Alizadeh, H. Tang, D. Lv, R. Starr, A. Brito, Q. Wu, L. J. Y. Kim, Z. Qiu, P. Lin, M. H. Lorenzini, B. Badie, S. J. Forman, Q. Xie, C. E. Brown and J. N. Rich (2021). "CRISPR Screening of CAR T Cells and Cancer Stem Cells Reveals Critical Dependencies for Cell-Based Therapies." Cancer Discov **11**(5): 1192-1211.

Wang, H., Z. Wu, D. Cui, L. Bian, Z. Zheng, J. Zhu, H. Geng, Z. Sun, Y. Pan, Y. Shi, Q. Yi, Z. Song, Y. Li, K. Shen, Y. Li, W. Shen, H. Yan, R. Hao, M. Sun, S. Zhang, C. Zhang, H. Jin and B. Zhai (2025). "Triple knockdown of CD11a, CD49d, and PSGL1 in T cells reduces CAR-T cell toxicity but preserves activity against solid tumors in mice." Sci Transl Med **17**(782): eadl6432.

Wang, Z., N. Li, K. Feng, M. Chen, Y. Zhang, Y. Liu, Q. Yang, J. Nie, N. Tang, X. Zhang, C. Cheng, L. Shen, J. He, X. Ye, W. Cao, H. Wang and W. Han (2021). "Phase I study of CAR-T cells with PD-1 and TCR disruption in mesothelin-positive solid tumors." Cell Mol Immunol **18**(9): 2188-2198.

Wang, Z. G., M. X. Chen, Y. Zhang, Y. Liu, Q. M. Yang, J. Nie, L. J. Shen, P. F. Jiang, J. P. He, X. Ye, W. Cao, H. Y. Wang and W. D. Han (2020). "Phase I study of CRISPR-engineered CAR-T cells with PD-1 inactivation in treating mesothelin-positive solid tumors." Journal of Clinical Oncology **38**(15).

Ward, M., L. Senaoui, B. Youngblood, M. Roussel and G. Krenciute (2024). "MODIFYING CAR T CELL EPIGENETIC PROGRAMS TO IMPROVE THEIR PERSISTENCE AGAINST GROUP 3 MEDULLOBLASTOMAS." Neuro-Oncology **26**: viii236.

Ward, M. B., A. O. Adeshakin, J. Ibanez, H. Sheppard, A. Zhou, J. J. Park, S. Gottschalk, H. Chi and G. Krenciute (2024). "REGNASE-1 DELETION IN CAR T CELLS ELICITS EXCELLENT TUMOR CLEARANCE BUT NEUROTOXICITY IN IMMUNODEFICIENT AND IMMUNOCOMPETENT BRAIN TUMOR MODELS." Journal for ImmunoTherapy of Cancer **12**: A532.

Wei, J., C. Luo, Y. Wang, Y. Guo, H. Dai, C. Tong, D. Ti, Z. Wu and W. Han (2019). "PD-1 silencing impairs the anti-tumor function of chimeric antigen receptor modified T cells by inhibiting proliferation activity." J Immunother Cancer **7**(1): 209.

Wellhausen, N., R. P. O'Connell, S. Lesch, N. W. Engel, A. K. Rennels, D. Gonzales, F. Herbst, R. M. Young, K. C. Garcia, D. Weiner, C. H. June and S. I. Gill (2023). "Epitope base editing CD45 in hematopoietic cells enables universal blood cancer immune therapy." Sci Transl Med **15**(714): eadi1145.

Wen, H., G. Huo, T. Hou, Z. Qu, J. Sun, Z. Yu, L. Kang, M. Wang, X. Lou, L. Yu and Y. Huo (2021). "Preclinical efficacy and safety evaluation of interleukin-6-knockdown CAR-T cells targeting at CD19." Ann Transl Med **9**(23): 1713.

Wiebking, V., C. M. Lee, N. Mostrel, P. Lahiri, R. Bak, G. Bao, M. G. Roncarolo, A. Bertaina and M. H. Porteus (2021). "Genome editing of donor-derived T-cells to generate allogenic chimeric antigen receptor-modified T cells: Optimizing αβ T cell-depleted haploidentical hematopoietic stem cell transplantation." Haematologica **106**(3): 847-858.

Williams, A., R. Mbofung, D. Morales-Mantilla, B. Groff, A. O'Connor, B. Li, R. Janani, B. Rezner, M. Jelcic, Y. Pan, T. Lee, K. J. Malmberg, M. Mamonkin, J. Goulding, J. Goodridge and B. Valamehr (2024). Alloimmune Defense Receptor Combined with Genetic Ablation of Adhesion Ligand CD58 Is a Comprehensive Approach to Promote Functional Persistence of Allogeneic Cell Therapies without Conditioning Chemotherapy. **144:** 503.

Wu, M. H., E. L. Giddings, F. Valenca-Pereira, C. Pham-Danis, F. Cendali, M. C. Yarnell, A. J. Novak, J. Henao-Mejia, R. A. Flavell, A. D'Alessandro, M. E. Kohler and M. Rincon (2024). "Increasing mitochondrial respiration by silencing MCJ/DnaJC15 enhances CD8 CAR-T cell therapy efficacy." Cancer Research **84**(6).

Xia, H., L. Peng, B. Engels, X. Xie, S. Sharma, N. Guo, A. K. Sohoni, C. Guimaraes, J. Brogdon, G. Dranoff and Y. Yang (2023). "Removal of Endogenous T Cell Receptor (TCR) May Inadvertently Compromise Allogeneic CAR T Cell Function." Molecular Therapy **31**(4): 146.

Xue, S. L., X. W. Tang, M. J. Liu, C. S. Qian, S. N. Chen, H. Y. Qiu, L. Q. Kang, H. P. Dai, W. J. Gong, L. Y. Chen, Z. Yao, M. Z. Xu, H. X. Zhou, X. F. Yang, Q. Wu, X. Q. Dou, J. Zhang, Y. Liu, Q. Y. Cui, Z. Li, Y. Xu, M. Qiao, T. T. Zhang, J. W. Tan, N. Xu, M. H. Li, Z. Yu, X. Y. Lou, W. Wang, H. J. Zhu, L. Qin, L. Yu and D. P. Wu (2025). IL-6 Knockdown Anti-CD19 CAR-T Cells (ssCART-19) for Patients with Relapsed or Refractory Acute Lymphoblastic Leukaemia: Phase 1 Trial.

Yang, J., J. Li, X. Zhang, L. Qiu and P. Lu (2022). "A NOVEL AND SUCCESSFUL CD7 GENE KNOCKOUT CAR-T CELL THERAPY FOR RELAPSED OR REFRACTORY T-CELL HEMATOLOGIC MALIGNANCIES." HemaSphere **6**: 23-24.

Yang, Z., L. Li, A. Turkoz, P. Chen, R. Harari-Steinfeld, M. Bobbin, O. Stefanson, H. Choi, V. Pietrobon, B. Alphson, A. Goswami, V. Balan, A. Kearney, D. Patel, J. Yang, D. Inel, V. Vinod, A. Cesano, B. Wang, K. H. Roh, L. S. Qi and F. M. Marincola (2021). "Contextual reprogramming of CAR-T cells for treatment of HER2(+) cancers." J Transl Med **19**(1): 459.

Yoshikawa, T., Z. Wu, S. Inoue, H. Kasuya, H. Matsushita, Y. Takahashi, H. Kuroda, W. Hosoda, S. Suzuki and Y. Kagoya (2021). "Genetic ablation of PRDM1 in antitumor T cells enhances therapeutic efficacy of adoptive immunotherapy." Blood.

Yoshikawa, T., Z. W. Wu, S. Inoue, H. Kasuya, H. Matsushita, Y. Takahashi, H. Kuroda, W. Hosoda, S. Suzuki and Y. Kagoya (2022). "Genetic ablation of PRDM1 in antitumor T cells enhances therapeutic efficacy of adoptive immunotherapy." Blood **139**(14): 2156-2172.

Zhang, H., J. Yao, I. Ajmal, M. A. Farooq and W. Jiang (2024). "shRNA-mediated gene silencing of HDAC11 empowers CAR-T cells against prostate cancer." Front Immunol **15**: 1369406.

Zhang, P., P. Ying, H. Li, N. Zhao, R. Liu, S. Li, W. Xu, Y. Tang and Y. Tang (2024). "A novel safer CD19CAR with shRNA interference of IFN-γ can reduce multiple cytokine levels without significantly compromising its killing efficacy." Apoptosis **29**(3-4): 556-567.

Zhang, Q., Y. H. Wu, J. Y. Yang, E. Manoharan, T. Wentz, A. Yu, K. Zhou, M. Hresko, L. Su, D. Ryan, C. Garcia and M. Milone (2024). "Engineered orthoIL2-CAR T Cells Generated Using Viral Vector-Free, Three-in-One Nonviral CRISPR-Based Technology Demonstrate Enhanced Antitumor Activity in a Murine Model of Leukemia." Molecular Therapy **32**(4): 831-832.

Zhang, X., C. Zhang, M. Qiao, C. Cheng, N. Tang, S. Lu, W. Sun, B. Xu, Y. Cao, X. Wei, Y. Wang, W. Han and H. Wang (2022). "Depletion of BATF in CAR-T cells enhances antitumor activity by inducing resistance against exhaustion and formation of central memory cells." Cancer Cell **40**(11): 1407-1422.e1407.

Zhang, Y., W. Luo, C. Li, M. Du, F. Zhou, L. Tang, J. Wu, H. Jiang, Q. Wei, C. Lu, H. Kou, D. Wu, A. H. Chang, Y. Hu and H. Mei (2022). "CD7 CHIMERIC ANTIGEN RECEPTOR T CELLS FOR ADULT PATIENTS WITH REFRACTORY AND RELAPSED T CELL MALIGNANCIES." HemaSphere **6**: 2571-2572.

Zhang, Y., X. Zhang, C. Cheng, W. Mu, X. Liu, N. Li, X. Wei, X. Liu, C. Xia and H. Wang (2017). "CRISPR-Cas9 mediated LAG-3 disruption in CAR-T cells." Front Med **11**(4): 554-562.

Zheng, B. A., Y. X. Hu, J. Q. Zhang, M. M. Zhang, W. Li, W. J. Wu, J. Z. Cui, G. Q. Wei, B. Du, M. Y. Liu and H. Huang (2024). "Long term follow-up results of BRL-201 phase I study, a CRISPR-based non-viral PD-1 locus specific integrated anti-CD19 CAR-T cells in treating relapsed or refractory non-Hodgkin's lymphoma." Journal of Clinical Oncology **42**(16).

Zhou, J. E., J. Yu, Y. Wang, H. Wang, J. Wang, Y. Wang, L. Yu and Z. Yan (2021). "ShRNA-mediated silencing of PD-1 augments the efficacy of chimeric antigen receptor T cells on subcutaneous prostate and leukemia xenograft." Biomed Pharmacother **137**: 111339.

Zhu, H., Y. You, Z. Shen and L. Shi (2020). "EGFRvIII-CAR-T Cells with PD-1 Knockout Have Improved Anti-Glioma Activity." Pathol Oncol Res **26**(4): 2135-2141.

Zhu, Z., H. Li, Q. Lu, Z. Zhang, J. Li, Z. Wang, N. Yang, Z. Yu, C. Yang, Y. Chen, H. Lu, W. Wang, T. Niu, C. Nie and A. Tong (2024). "mRNA-Engineered CD5-CAR-γδT(CD5-) Cells for the Immunotherapy of T-Cell Acute Lymphoblastic Leukemia." Adv Sci (Weinh) **11**(35): e2400024.

Zou, Y., B. Liu, L. Li, Q. Yin, J. Tang, Z. Jing, X. Huang, X. Zhu and T. Chi (2022). "IKZF3 deficiency potentiates chimeric antigen receptor T cells targeting solid tumors." Cancer Lett **524**: 121-130.
